# Design and Synthesis of Novel α-Methylchalcone Derivatives, Anti-Cervical Cancer Activity, and Reversal of Drug Resistance in HeLa/DDP Cells

**DOI:** 10.3390/molecules28237697

**Published:** 2023-11-21

**Authors:** Zheng Yang, Zhengye Liu, Mourboul Ablise, Aikebaier Maimaiti, Aizitiaili Aihaiti, Yusupuwajimu Alimujiang

**Affiliations:** The Xinjiang Key Laboratory of Natural Medicine Active Components and Drug Release Technology, College of Pharmacy, Xinjiang Medical University, Urumqi 830011, China; qhyzcr@163.com (Z.Y.); lzy17599832619@163.com (Z.L.); akpar15@163.com (A.M.); m17590823808@163.com (A.A.); 13209922075@163.com (Y.A.)

**Keywords:** α-methyl chalcone, cervical cancer, cisplatin resistance, tubulin, P-gp

## Abstract

In this study, a collection of newly developed α-methylchalcone derivatives were synthesized and assessed for their inhibitory potential against human cervical cancer cell lines (HeLa, SiHa, and C33A) as well as normal human cervical epithelial cells (H8). Notably, compound **3k** exhibited substantial inhibitory effects on both HeLa and HeLa/DDP cells while demonstrating lower toxicity toward H8 cells. Furthermore, the compound **3k** was found to induce apoptosis in both HeLa and HeLa/DDP cells while also inhibiting the G2/M phase, resulting in a decrease in the invasion and migration capabilities of these cells. When administered alongside cisplatin, **3k** demonstrated a significant reduction in the resistance of HeLa/DDP cells to cisplatin, as evidenced by a decrease in the resistance index (RI) value from 7.90 to 2.10. Initial investigations into the underlying mechanism revealed that **3k** did not impact the expression of P-gp but instead facilitated the accumulation of rhodamine 123 in HeLa/DDP cells. The results obtained from CADD docking analysis demonstrated that **3k** exhibits stable binding to microtubule proteins and P-gp targets, forming hydrogen bonding interaction forces. Immunofluorescence analysis further revealed that **3k** effectively decreased the fluorescence intensity of α and β microtubules in HeLa and HeLa/DDP cells, resulting in disruptions in cell morphology, reduction in cell numbers, nucleus coagulation, and cell rupture. Additionally, Western blot analysis indicated that **3k** significantly reduced the levels of polymerized α and β microtubule proteins in both HeLa and HeLa/DDP cell lines while concurrently increasing the expression of dissociated α and β microtubule proteins. The aforementioned findings indicate a potential correlation between the inhibitory effects of **3k** on HeLa and HeLa/DDP cells and its ability to inhibit tubulin and P-gp.

## 1. Introduction

Cervical cancer, which in 2020 accounted for 3.4% of all cancer deaths among women, with 342,000 fatalities and 604,000 new cases worldwide, ranks as the fourth leading cause of cancer deaths [1]. The primary treatment options for cervical cancer include surgery, radiotherapy, and chemotherapy, with cisplatin serving as the first-line chemotherapy drug. Nevertheless, several factors, such as limited selectivity, serious side effects, tumor multidrug resistance, and easy recurrence, can render these treatments ineffective [2].

Multidrug resistance (MDR) of tumors is the primary reason for the failure of chemotherapy in patients with cervical cancer. MDR is characterized by the development of cross-resistance to antineoplastic drugs with varying structural types and mechanisms, which occurs as a result of prolonged chemotherapy use [3]. The human body contains 48 ABC transporters, with ABCB1, ABCC1, and ABCG2 being closely associated with tumor MDR [4]. Moreover, studies have shown that ABCB1, commonly referred to as P-glycoprotein (P-gp), is responsible for the efflux of cisplatin from tumor cells, leading to drug resistance in cervical cancer [5]. The upregulation of P-gp has been extensively established as the primary cause of multidrug resistance in cervical cancer, with high expression of P-gp in tumor cells inducing resistance to cisplatin [6].

Microtubule targeting agents (MTAs) have been extensively employed as clinical anticancer drugs for several decades. Nevertheless, their use is significantly limited due to the development of acquired drug resistance [7]. In recent times, the identification of anti-tumor drug resistance regulators that target the tubulin colchicine binding site has emerged as a novel and promising area of research. The majority of drugs that interact with the colchicine site exhibit non-substrate characteristics toward P-gp and possess the ability to circumvent the P-gp-mediated mechanism of multidrug resistance (MDR) in antitumor treatment. Furthermore, these drugs primarily consist of small molecules [8]. It is widely acknowledged that chalcone, due to its structural similarities to the anticancer medication combretastatinA-4 (CA-4), may target tubulin. Recent research has provided evidence that chalcone can arrest the cell cycle in the G2/M phase and induce apoptosis in cancer cells by acting on the colchicine site of tubulin [9].

**Figure 1:**
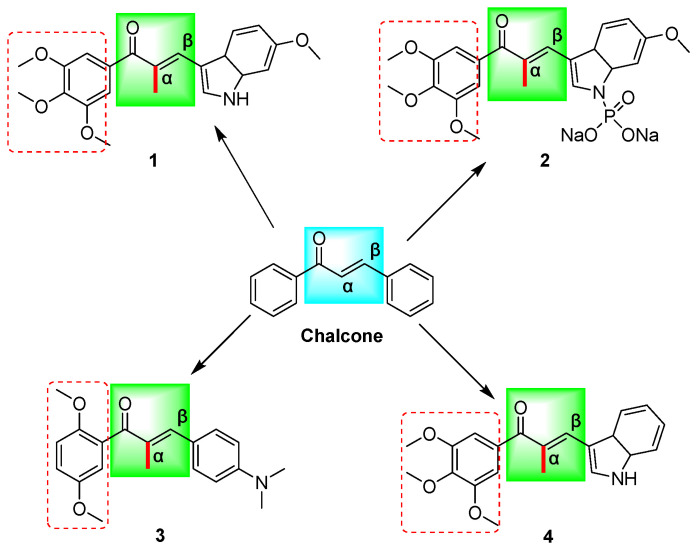
The chemical structures of chalcone and α-methyl chalcone derivatives (**1**–**4**) reported in the above literature are schematically illustrated; (Chalcone: *(E)-chalcone*; **1**: *(E)-3-(6-methoxy-3a,7a-dihydro-1H-indol-3-yl)-2-methyl-1-(3,4,5-trimethoxyphenyl)prop-2-en-1-one*; **2**: *sodium (E)-(6-methoxy-3-(2-methyl-3-oxo-3-(3,4,5-trimethoxyphenyl)prop-1-en-1-yl)-3a,7a-dihydro-1H-indol-1-yl)phosphonate*; **3**: *(E)-1-(2,5-dimethoxyphenyl)-3-(4-(dimethylamino)phenyl)-2-methylprop-2-en-1-one*; **4**: *(E)-3-(3a,7a-dihydro-1H-indol-3-yl)-2-methyl-1-(3,4,5-trimethoxyphenyl)prop-2-en-1-one)*. Red boxes represent methoxy substitution.

Chalcones (Figure 1) are substances containing an α,β-unsaturated carbonyl system (C=CCO) with the parent compound 1,3-diphenyl-1,2-propen-1-one being a bioactive naturally occurring constituent of medicinal plants such as licorice and safflower [10,11]. This chalcone demonstrates distinctive biological characteristics, encompassing anticancer [12], antibacterial [13], anti-inflammatory [14], antiviral [15], antitubercular [16], antidepressant [17], and antimalarial [18] properties, among numerous other pharmacological effects. The chalcone mother nucleus facilitates diverse biological activities via the incorporation of multiple functional groups, including aryl, halogen, hydroxyl, carboxyl, and phenyl, among others [19]. Representative mechanisms of chalcones’ anticancer effects include MDM2/p53, tubulin, NF-kB, Wnt/β-catenin, VEGF/VEGFR-2, HIF-1, MMP-2/9, and P-gp/MRP1/BCRP [12]. These mechanisms have been shown to induce apoptosis in cancer cells [20], arrest the cell cycle [21], inhibit viability and proliferation, prevent invasion and migration [22], and promote autophagy [12] in response to the development of multidrug resistance in tumors.

Chalcone has been documented to function as a Michael acceptor, effectively alkylating numerous essential proteins within the microtubulin–microtubule system. Consequently, chalcone is regarded as a promising framework for the conceptualization and advancement of innovative anticancer therapeutics [23]. Previous research has demonstrated that compounds **1**–**4** (Figure 1) exhibit noteworthy efficacy against multidrug-resistant tumor cells, with microtubule proteins being identified as potential targets for their mechanism of action [9,24,25]. Structural analysis has revealed that the structures of these compounds contain α-methyl, methoxy, and indole rings in addition to the chalcone parent nucleus skeleton. Further investigation has indicated that the cytotoxicity of these compounds may be significantly influenced by the number and position of the methoxy substituents on the aromatic ring [12] (Figure 1). In our prior investigations, we design and synthesize a range of chalcone derivatives that were substituted by α-methyl, halogen, p-dimethylamine, methoxy, and hydroxyl groups. Subsequently, we assessed their effectiveness in inhibiting the proliferation of cervical cancer cell lines. The results indicated that these derivatives exhibited noteworthy anti-cervical cancer properties while exhibiting minimal toxicity toward normal cells [26]. The methoxylation of chalcone was discovered to have a significant impact on the inhibitory potency and cytotoxicity of selective inhibitors targeting drug-resistant proteins in breast cancer [27]. The presence of methoxy substituents in the backbone is considered a significant determinant of the cytotoxicity of chalcone against multidrug-resistant cancer cells, with chalcone containing two methoxyl groups displaying superior activity compared to chalcone with a solitary methoxyl group [28].

**Figure 2:**
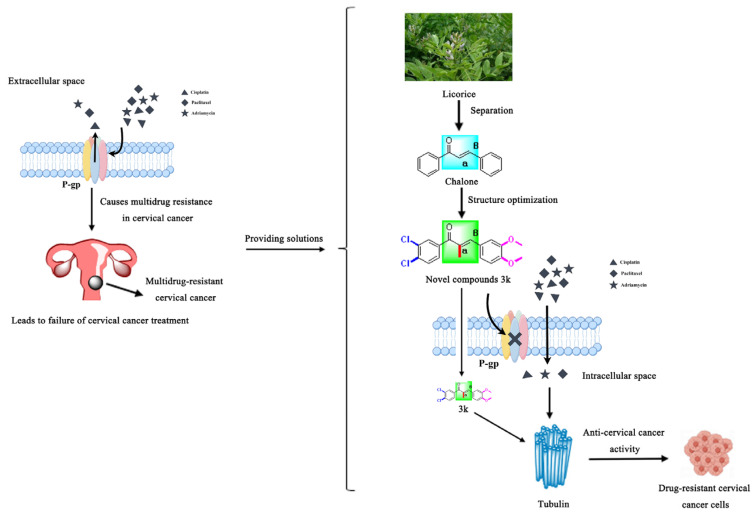

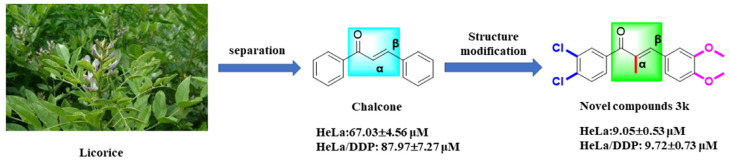
Schematic diagram of the rationale for the present study.

Drawing on prior research, we synthesized a collection of innovative chalcone derivatives by employing chalcone, a naturally occurring bioactive constituent found in licorice, as a foundational compound. We conducted an assessment of their effectiveness against cervical cancer and cisplatin-resistant cervical cancer. The current investigation furnishes an empirical foundation for the novel molecularly targeted α-methylchalcone derivatives, which are predicated on tubulin/P-gp targets, in terms of their efficacy against multidrug-resistant cervical cancer (Figure 2).

## 2. Results and Discussion

### 2.1. Chemistry

**Scheme 1:**
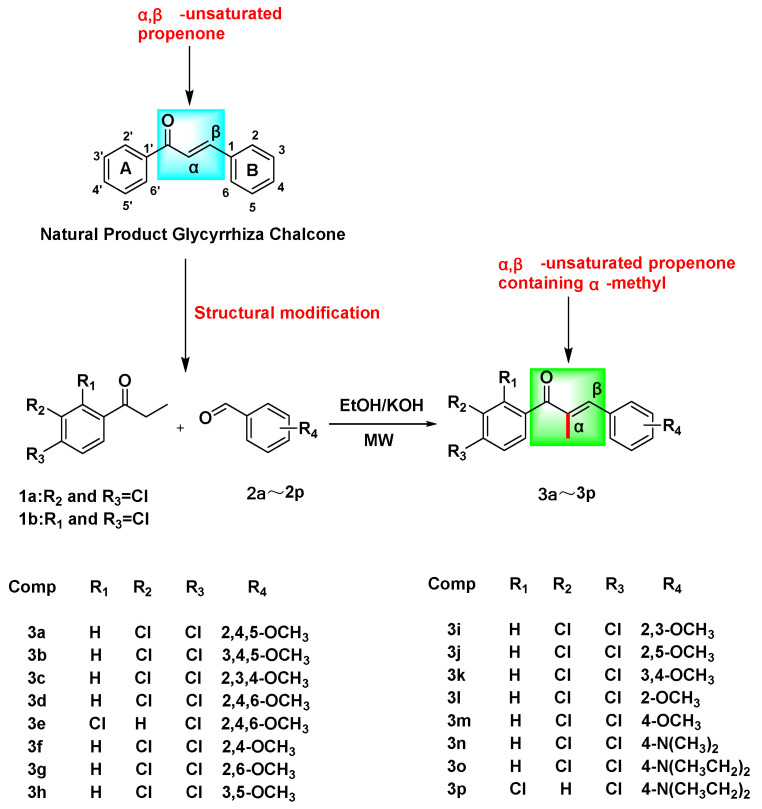
The synthetic route to target compounds **3a**–**3p**.

In this investigation, phenylacetone and benzaldehyde derivatives were introduced into a three-necked flask containing anhydrous ethanol, with a 20% KOH solution serving as the catalyst. The reaction was conducted utilizing microwave heating conditions, resulting in the synthesis of sixteen novel α-methyl chalcone derivatives, designated as **3a**–**3p** (Scheme 1).

In the initial phase of the experiment, the research team employed piperidine as a catalyst for the synthesis of α-methyl chalcone derivatives, necessitating an 18 h reaction time [26]. Piperidine belongs to the controlled class of readily toxic reagents and, as a catalyst, requires a long reaction time and is not readily available. In this investigation, the team sought to substitute piperidine with a 20% KOH solution as the catalyst, conducting the reaction under ambient temperature and heating conditions ranging from 45 to 100 °C. However, only the synthesis of compounds **3a**–**3e** was achieved, albeit with a reaction time of 24 h, incomplete reaction, and complex purification and separation processes. Consequently, we employed the microwave liquid-phase reaction technique, which enabled the successful synthesis of all desired compounds within a reaction time range of 2.5–7 min and under the conditions of 100 W. In comparison to reactions conducted at room temperature or with heating, the microwave liquid-phase reaction method offers several advantages, including shorter reaction times, minimal formation of by-products, high yield, and simplified purification and separation procedures.

### 2.2. In Vitro Activity Assays

#### 2.2.1. Anti-Cervical Cancer Activity Assay

**Scheme 2:**
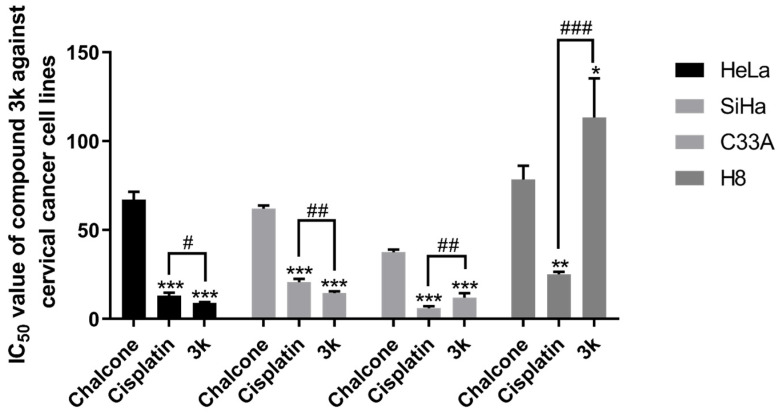
IC_50_ values of the lead compound chalcone, the positive drug cisplatin, and compound **3k** on cervical cancer cell lines. All the above results were expressed as the mean ± SD of three independent experiments: * *p* < 0.05, ** *p* < 0.01, *** *p* < 0.001 compared with the chalcone group; # *p* < 0.05, ## *p* < 0.01, ### *p* < 0.001 compared with the cisplatin group.

The results of the MTT assay demonstrated that compounds **3a**–**3p** displayed a certain degree of inhibitory activity against the proliferation of human cervical cancer cell lines HeLa, SiHa, and C33A. Additionally, these compounds exhibited minimal toxicity toward human cervical epithelial cells H8. Compound **3k** exhibited the most notable anti-cervical cancer activity among the compounds tested. Its IC_50_ value against HeLa cells was 9.05 ± 0.53 μM, significantly lower than that of the lead compound chalcone (*p* < 0.001) and the positive drug cisplatin group (*p* < 0.05). Moreover, these compounds exhibited minimal toxicity toward human cervical epithelial cells H8; its IC_50_ value against SiHa cells was 14.54 ± 0.98 μM, significantly lower than that of chalcone (*p* < 0.001) and the cisplatin group (*p* < 0.01). Furthermore, the IC_50_ value for C33A cells was 12.11 ± 2.44 μM, significantly lower than that of the chalcone group (*p* < 0.001). The IC_50_ value for H8 cells was found to be greater than 100 μM, a significantly higher value compared to both the chalcone group (*p* < 0.05) and the cisplatin group (*p* < 0.001) (Table 1, Scheme 2).

Furthermore, the compound **3k** exhibited a notably greater capacity for inhibiting proliferation in HeLa, SiHa, and C33A cells compared to other compounds. This enhanced activity may be attributed to the incorporation of α-methyl, halogen, and methoxy moieties into the structure of **3k**, which is in line with the findings of a prior investigation [26]. Subsequent investigations will delve into the impact of **3k** on pro-apoptotic mechanisms, cell cycle arrest, and invasive migration in HeLa cells. The results of the structural analysis indicate that the substitution of the A ring of chalcone with 3,4-Cl and the B ring with 3,4-OCH_3_ resulted in the compound **3k** exhibiting greater inhibitory activity against HeLa, SiHa, and C33A cells while demonstrating lower toxicity against H8 cells. These findings suggest that 3,4-Cl and 3,4-OCH_3_ may be the essential active groups, and their substitution positions should be maintained in future compound designs.

#### 2.2.2. Structure–Effect Relationship Analysis

The compounds **3a**–**3p** had the following effects on HeLa, SiHa, C33A, and H8 cells for 48 h:

(1) Upon substitution of the A-ring of the chalcone parent nucleus with 3,4-dichloro and the B-ring with 3,4,5-OCH_3_, 2,4,6-OCH_3_, 2,6-OCH_3_, 3,4-OCH_3_, and 4-OCH_3_, the resulting compounds exhibited varying degrees of proliferation-inhibitory activity against HeLa cells, with the order of magnitude being as follows: 3,4-OCH_3_ > 4-OCH_3_ > 3,4,5-OCH_3_ > 2,6-OCH_3_ > 2,4,6-OCH_3_.

(2) Upon substitution of the A-ring of the chalcone parent nucleus with 3,4-dichloro and the B-ring with 2,4,5-OCH_3_, 3,4,5-OCH_3_, 2,3,4-OCH_3_, 3,4-OCH_3_, 2-OCH_3_, and 4-OCH_3_, the resulting compounds exhibited varying degrees of proliferation-inhibitory activity on SiHa cells was: 3,4-OCH_3_ > 2-OCH_3_ > 2,4,5-OCH_3_ > 3,4,5-OCH_3_ > 2,3,4-OCH_3_ > 4-OCH_3_.

(3) Upon substitution of the A-ring of the chalcone parent nucleus with 3,4-dichloro and the B-ring with 2,4-OCH_3_, 2,5-OCH_3_, 3,4-OCH_3_, 2-OCH_3_, and 4-N(CH_3_)_2_, the resulting compounds exhibited varying degrees of proliferation-inhibitory activity on C33A cells was: 3,4-OCH_3_ > 2-OCH_3_ > 4-N(CH_3_)_2_ > 2,4-OCH_3_ > 2,5-OCH_3_.

#### 2.2.3. Apoptosis of HeLa Cells

**Scheme 3:**
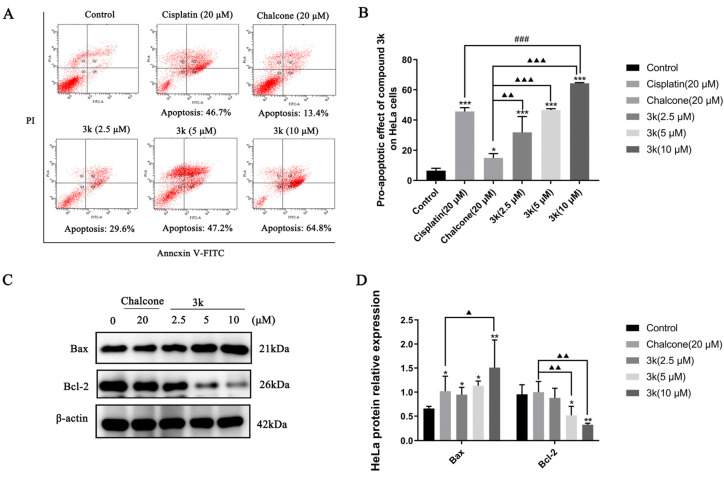
(**A**) Pro-apoptotic effect of compound **3k** on HeLa cells after 24 h of action. (**B**) Quantitative analysis of the pro-apoptotic effect. (**C**) Changes in the expression of pro-apoptotic protein Bax and anti-apoptotic protein Bcl-2 after 24 h of compound **3k** action on HeLa cells. (**D**) Quantitative analysis graph of the expression of pro-apoptotic protein Bax and anti-apoptotic protein Bcl-2. All the above results were expressed as the mean ± SD of three independent experiments: * *p* < 0.05, ** *p* < 0.01, *** *p* < 0.001 compared with the control group; ### *p* < 0.001 compared with the cisplatin group; ▲ *p* < 0.05, ▲▲ *p* < 0.01, ▲▲▲ *p* < 0.001 compared with the chalcone group.

The findings of the study demonstrated that compound **3k** exhibited a dose-dependent pro-apoptotic effect on HeLa cells. The pro-apoptotic rates of 29.6%, 47.2%, and 64.8% were observed at concentrations of 2.5, 5, and 10 μM, respectively. These rates were significantly higher compared to the control group (*p* < 0.001). Furthermore, the pro-apoptotic effect of compound **3k** was found to be significantly greater than that of the lead compound chalcone group, with statistically significant differences observed at *p* < 0.01, *p* < 0.001, and *p* < 0.001. Furthermore, the apoptotic impact of **3k** on HeLa cells was of a significantly greater magnitude compared to the positive drug cisplatin group at a concentration of 10 μM (*p* < 0.001) (Scheme 3A,B).

The Western blot analysis revealed a significant upregulation in Bax expression at concentrations of 2.5, 5, and 10 μM of **3k**, as compared to the control group, with statistically significant differences observed at *p* < 0.05, *p* < 0.05, and *p* < 0.01, respectively. Furthermore, the upregulation of Bax expression by **3k** at a concentration of 10 μM was significantly higher than that observed in the chalcone group (*p* < 0.05). The downregulation of Bcl-2 expression exhibited a significantly greater magnitude in comparison to the control group at concentrations of 5 and 10 μM of **3k**, with statistically significant differences observed at *p* < 0.05 and *p* < 0.01, respectively. Likewise, the downregulation of Bcl-2 expression was significantly higher than that of the chalcone group (*p* < 0.01) at concentrations of 5 and 10 μM of **3k**. (Scheme 3C,D).

The process of apoptosis requires the activation, expression, and regulation of a series of intracellular pathway proteins that ultimately result in programmed cell death, which is a vital component in the maintenance of tissue homeostasis [29]. An annexin V-FITC/propidium iodide (PI) double staining assay was conducted to elucidate the mechanism of action of **3k** on HeLa cells. Our findings indicate that compound **3k** elicits early and late apoptosis in HeLa cells in a concentration-dependent manner. Moreover, the Western blot analysis demonstrated that the expression of the pro-apoptotic protein Bax was elevated while the expression of the anti-apoptotic protein Bcl-2 was decreased in a concentration-dependent manner by compound **3k**. Therefore, it can be inferred that the apoptotic pathway is mediated by compound **3k**, leading to the downregulation of Bcl-2 and upregulation of Bax expression, ultimately resulting in the apoptosis of HeLa cells.

#### 2.2.4. Cell Cycle Experiment of HeLa Cells

**Scheme 4:**
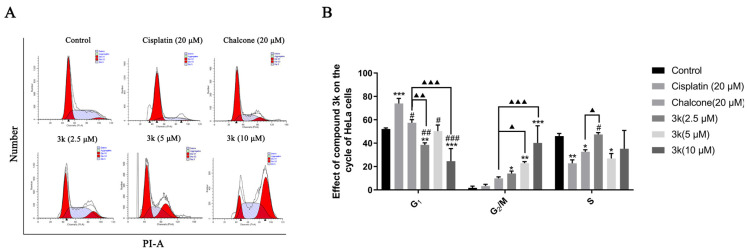
(**A**) Cell cycle effects of **3k** and cisplatin on HeLa. (**B**) Quantitative analysis of the effect of **3k** on the HeLa cell cycle in the bar graph. All the above results were expressed as the mean ± SD of three independent experiments: * *p* < 0.05, ** *p* < 0.01, *** *p* < 0.001 compared with the control group; # *p* < 0.05, ## *p* < 0.01, ### *p* < 0.001 compared with the cisplatin group; ▲ *p* < 0.05, ▲▲ *p* < 0.01, ▲▲▲ *p* < 0.001 compared with the chalcone group.

The findings of the study indicate that compound **3k**, when administered at concentrations of 2.5, 5, and 10 μM, primarily impeded the progression of HeLa cells at the G2/M phase. The blocking rates observed were 14.04%, 22.84%, and 40.18%, respectively. These rates were significantly greater than those observed in the control group, with statistically significant differences of *p* < 0.05, *p* < 0.01, and *p* < 0.001, respectively. Furthermore, at concentrations of 5 and 10 μM, compound **3k** exhibited significantly higher blocking rates on HeLa cells at the G2/M phase compared to the chalcone group, with statistical differences of *p* < 0.05 and *p* < 0.001, respectively. Furthermore, the inhibitory effect of cisplatin on HeLa cells in the G0/G1 phase exhibited a significant increase, rising from 52.1% to 73.93% when compared to the control group (*p* < 0.001) (Scheme 4A,B).

Chalcones have been identified as Michael acceptors and have been shown to alkylate numerous essential proteins of the microtubulin–microtubule system [23]. Investigations have established that chalcones that target colchicine sites on microtubulin can impede the cell cycle in the G2/M phase and trigger apoptosis [30]. In the present study, compound **3k** demonstrated a concentration-dependent inhibitory effect on HeLa cells in the G2/M phase, thereby disrupting the mitotic process and ultimately resulting in the inhibition of proliferation and induction of apoptosis in HeLa cells. Notably, the blocking effect of **3k** on HeLa cells in the G2/M phase was significantly augmented when compared to the lead compound chalcone. This suggests that the incorporation of α-methyl, 3,4-Cl, and 3,4-OCH_3_ groups into the parent nucleus of chalcone in the synthesized compound **3k** may enhance its inhibitory effect on the G2/M phase. The above results suggest that microtubulin may be the target of **3k**, and further validation is needed.

#### 2.2.5. Migration and Invasion of HeLa Cells

**Scheme 5:**
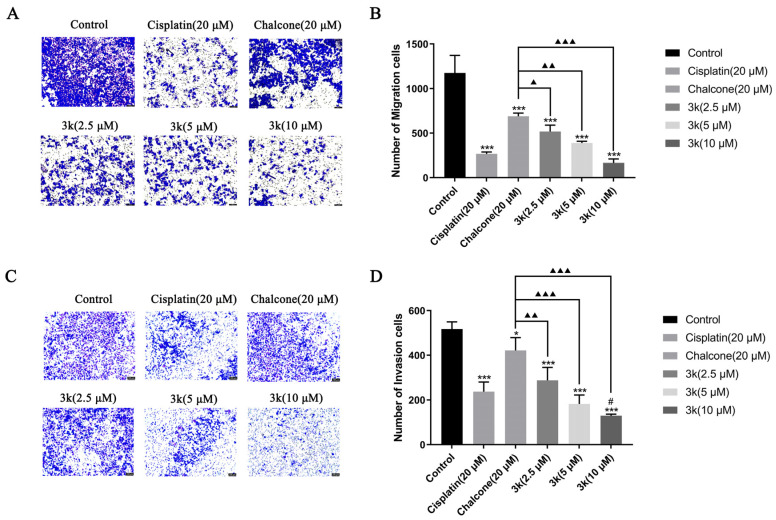
(**A**) Morphology of migrating cells after 24 h of compound **3k** on HeLa cells in a Transwell migration assay. (**B**) Quantitative analysis of the number of migrated cells. (**C**) Morphology of invaded cells after 24 h of compound **3k** action on HeLa cells in the Transwell invasion assay. (**D**) Quantitative analysis of the number of invaded cells; HeLa cell morphology was observed under an inverted microscope (magnification: ×100). All the above results were expressed as the mean ± SD of three independent experiments: * *p* < 0.05, *** *p* < 0.001 compared with the control group; # *p* < 0.05, compared with the cisplatin group; ▲ *p* < 0.05, ▲▲ *p* < 0.01, ▲▲▲ *p* < 0.001 compared with the chalcone group.

The findings of the study indicate that compound **3k** exhibited a significant inhibitory effect on the penetration of HeLa cells through the chamber membrane. The number of migrated cells decreased from 1176 in the control group to 518, 388, and 166 at concentrations of 2.5, 5, and 10 μM of **3k**, respectively. These values were significantly lower than those observed in the control group (*p* < 0.001). Furthermore, the number of migrated cells was also lower than that observed in the chalcone group (689) at concentrations of 2.5, 5, and 10 μM of **3k**, with statistical differences of *p* < 0.05, *p* < 0.01, and *p* < 0.001, respectively. (Scheme 5A,B). At concentrations of 2.5, 5, and 10 μM, the presence of **3k** resulted in a significant decrease in the number of cells undergoing invasion compared to the control group (*p* < 0.001). Apart from this, the number of cells undergoing invasion was significantly lower than that in the chalcone group (422) at the same concentrations of **3k**, with statistically significant differences of *p* < 0.01, *p* < 0.001, and *p* < 0.001. Additionally, when exposed to a concentration of 10 μM, the number of cells undergoing invasion at **3k** was significantly lower compared to the cisplatin group (237) (*p* < 0.05) (Scheme 5C,D).

The growth of tumors is facilitated by the migration, invasion, and metastasis of tumor cells, which involves the detachment of individual cells from the primary tumor and their infiltration into lymphatic vessels, blood, or other tissues [31]. Compound **3k** exhibited a dose-dependent inhibitory effect on HeLa cell invasion and migration, which was significantly more substantial than that of the positive drugs cisplatin and chalcone at 10 μM. These results suggest that **3k** may mitigate tumor cell invasion and migration. Despite advancements in chemotherapy, the toxicity of treatment and multidrug resistance in tumors remain the primary reasons for treatment failure in cervical cancer. The inhibitory activity of compound **3k** on HeLa cells, as well as its potential mechanism of action, have led us to explore its effectiveness against cervical cancer cell lines that are resistant to cisplatin.

#### 2.2.6. In Vitro Anti-HeLa/DDP Cell Activities

**Scheme 6:**
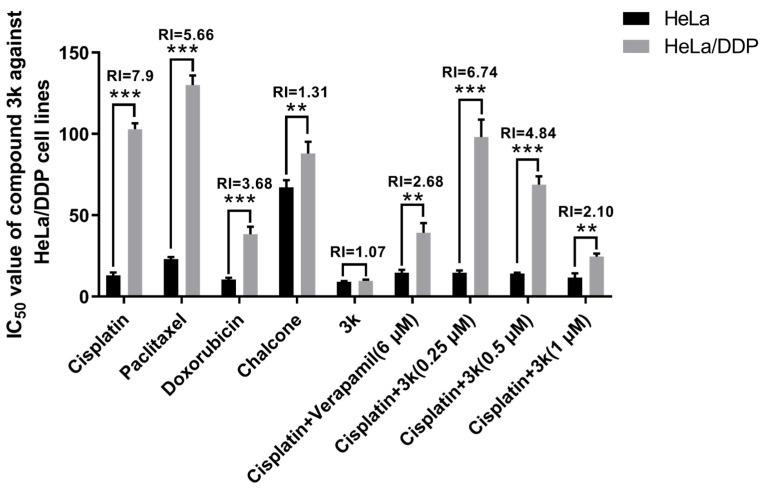
IC_50_ values of the lead compound chalcone, the positive drug cisplatin and compound **3k** on cervical cancer cell lines. All the above results were expressed as the mean ± SD of three independent experiments: ** *p* < 0.01, *** *p* < 0.001 compared with the HeLa/DDP group.

The findings indicated that the positive drugs cisplatin, paclitaxel, and adriamycin exhibited significantly elevated IC_50_ values against HeLa/DDP cells compared to the parental cells HeLa (*p* < 0.001), demonstrating a moderate level of resistance. The corresponding resistance indices (RI) for cisplatin, paclitaxel, and adriamycin were 7.90, 5.66, and 3.68, respectively. The IC_50_ values of compound **3k** against HeLa and HeLa/DDP cell lines were determined to be 9.05 ± 0.53 and 9.72 ± 0.73 μM (*p* > 0.05), respectively, with an RI < 1.2. Following a 48 h incubation of HeLa/DDP cells with a combination of **3k** (0.5 and 1 μM) and cisplatin, the IC_50_ values were determined to be 68.84 ± 5.10 and 24.54 ± 1.86 μM, respectively. These values were significantly lower than the IC_50_ values (102.91 ± 3.60 μM) observed when cisplatin alone was used (*p* < 0.001), resulting in reduced RI values of 4.84 and 2.10, respectively. When the P-gp inhibitor verapamil was administered simultaneously with cisplatin at a concentration of 6 μM, the IC_50_ value for HeLa/DDP cells was determined to be 39.13 ± 6.05 μM. This value was found to be significantly lower than that of the cisplatin group (*p* < 0.001), resulting in a decrease of the RI to 2.68 (Table 2, Scheme 6).

**Scheme 7:**
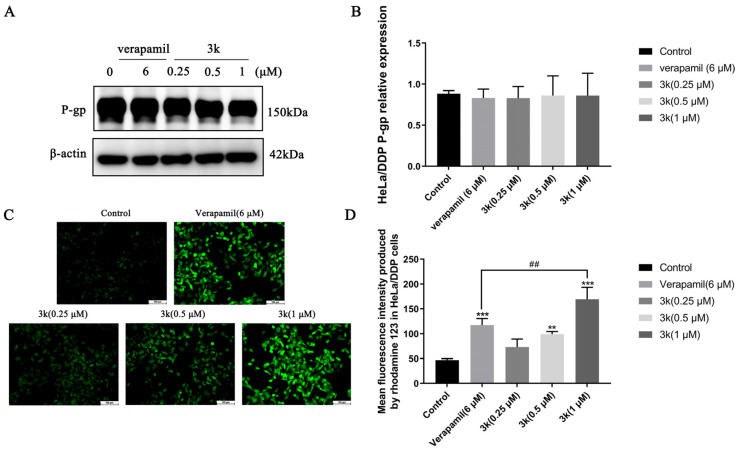
(**A**) P-gp protein expression after applying Compound **3k** to HeLa/DDP cells for 48 h. (**B**) Quantitative analysis of P-gp protein expression in HeLa/DDP cells. (**C**) Fluorescence intensity of rhodamine 123 after applying Compound **3k** to HeLa/DDP cells for 48 h. (**D**) Quantitative analysis of fluorescence intensity of rhodamine 123 in HeLa/DDP cells. All the above results were expressed as the mean ± SD of three independent experiments: ** *p* < 0.01, *** *p* < 0.001 compared with the control group; ## *p* < 0.01 compared with the verapamil group.

Western blotting analysis revealed that the expression of P-gp in HeLa/DDP cells remained unchanged when treated with compound **3k** at concentrations of 0.25, 0.5, or 1 μM, as indicated by the lack of statistical significance (*p* > 0.05) when compared to both the blank control and verapamil groups (Scheme 7A,B). The results of the Rhodamine 123 experiments indicate that the average fluorescence intensity emitted by rhodamine 123 in HeLa/DDP cells was notably greater than that of the control group at **3k** concentrations of 0.5 and 1 μM, with statistically significant differences observed at *p* < 0.01 and *p* < 0.001, respectively. Furthermore, the fluorescence intensity emitted by **3k** at a concentration of 1 μM was significantly higher than that emitted by verapamil (6 μM) in HeLa/DDP cells (*p* < 0.01) (Scheme 7C,D).

From a clinical perspective, the term MDR denotes the development of cross-resistance following prolonged administration of antineoplastic drugs and other structurally diverse drugs with varying mechanisms of action during chemotherapy [3]. The current investigation revealed that compound **3k** exhibited significantly superior inhibitory activity against HeLa/DDP cells compared to other compounds, with a resistance index (RI) of 1.07, indicating minimal drug re-resistance. Furthermore, compound **3k** demonstrated no discernible anti-proliferative activity within the concentration range of 0.25, 0.5, or 1 μM, with an inhibition rate of less than 10% against HeLa/DDP cell lines. The co-administration of **3k** and cisplatin within a specific concentration range resulted in a significant increase in the anti-proliferative activity of cisplatin against HeLa/DDP cells. Based on the initial findings from Western blot and rhodamine 123 efflux experiments, it can be inferred that the observed effect is potentially caused by the suppression of the P-gp protein transport mechanism on the membrane of HeLa/DDP cells by **3k**. Consequently, this inhibition results in an elevation of cisplatin accumulation within the cells, ultimately impeding the proliferation of HeLa/DDP cells.

#### 2.2.7. Apoptosis of HeLa/DDP Cells

**Scheme 8:**
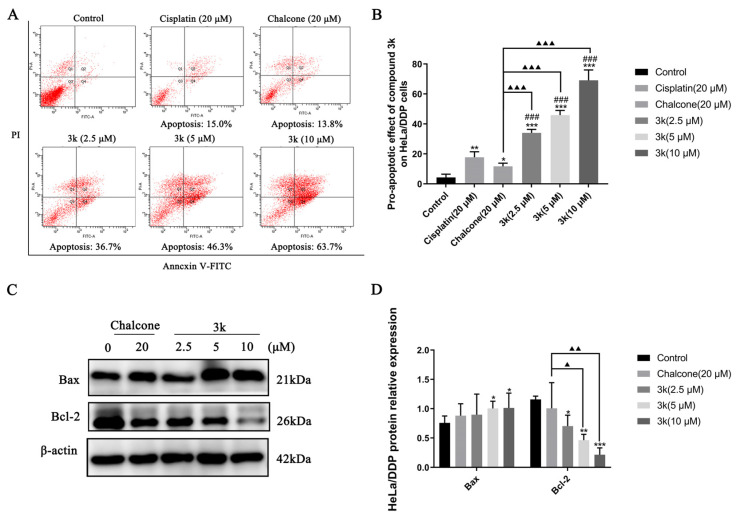
(**A**) Pro-apoptotic effect of compound **3k** on HeLa/DDP cells after 24 h of action. (**B**) Quantification of the pro-apoptotic effect on HeLa/DDP cells. (**C**) Changes in the expression of pro-apoptotic protein Bax and anti-apoptotic protein Bcl-2 in HeLa cells after 24 h of compound **3k** action. (**D**) Quantification of the expression of pro-apoptotic protein Bax and anti-apoptotic protein Bcl-2 in HeLa/DDP cells. All the above results were expressed as the mean ± SD of three independent experiments: * *p* < 0.05, ** *p* < 0.01, *** *p* < 0.001 compared with the control group; ### *p* < 0.001 compared with the cisplatin group; ▲ *p* < 0.05, ▲▲ *p* < 0.01, ▲▲▲ *p* < 0.001 compared with the chalcone group.

The findings of the study demonstrated that compound **3k** displayed concentration-dependent pro-apoptotic rates of 36.7%, 46.3%, and 63.7% at concentrations of 2.5, 5, and 10 μM, respectively, in HeLa/DDP cells. Furthermore, the pro-apoptotic rates of cisplatin and chalcone at a concentration of 20 μM were 15.0% and 13.8%, respectively, which were significantly lower than the pro-apoptotic effect of **3k** on HeLa/DDP cells (*p* < 0.001). Furthermore, the pro-apoptotic efficacy of cisplatin in HeLa/DDP cells was only 15.0% at 20 μM, which was significantly lower than its pro-apoptotic efficacy of 46.7% in HeLa cells (*p* < 0.001) (Scheme 8A,B).

Western blot analysis demonstrated that compound **3k** exhibited a significant increase in the expression of Bax in HeLa/DDP cells at concentrations of 5 and 10 μM compared to the control group (*p* < 0.05). What is more, **3k** was found to significantly downregulate the expression of Bcl-2 at concentrations of 2.5, 5, and 10 μM compared to the control group, with statistically significant differences observed at *p* < 0.05, *p* < 0.01, and *p* < 0.001, respectively. Furthermore, at concentrations of 5 and 10 μM, **3k** demonstrated a significant downregulation of Bcl-2 expression compared to the chalcone group, with statistical differences of *p* < 0.05 and *p* < 0.01, respectively (Scheme 8C,D).

The formation of multidrug resistance (MDR) is a complex process primarily associated with P-glycoprotein (P-gp) [32]. Extensive evidence has demonstrated that upregulation of P-gp is a major contributor to multidrug resistance in cervical cancer, while high expression of P-gp in tumor cells induces resistance to cisplatin in cervical cancer [6]. The findings presented demonstrate that the pro-apoptotic impact of compound **3k** on HeLa/DDP cells exhibited a concentration-dependent escalation and was significantly superior to that of the positive drug cisplatin across all concentration ranges. Furthermore, the pro-apoptotic rate of cisplatin on HeLa/DDP cells at 20 μM was notably lower than that of HeLa cells, potentially due to the elevated expression of P-gp in HeLa/DDP cells, which facilitated the extrusion of cisplatin from HeLa/DDP cells by P-gp, thereby diminishing the pro-apoptotic effect of cisplatin. Subsequent examination indicated that the impact of **3k** on promoting apoptosis in HeLa and HeLa/DDP cells was essentially equivalent, with no notable distinctions. This outcome may be attributed to the suppression of P-gp activity by **3k**, leading to the loss of P-gp’s capacity to transport compounds outside the cells. Western blot analysis showed that the pro-apoptotic effect of **3k** on HeLa/DDP cells was associated with upregulation of Bax and downregulation of Bcl-2. Chalcone analogs activate the expression of pro-apoptotic protein Bax and decrease the activity of anti-apoptotic protein Bcl-2, which in turn causes apoptosis [33].

#### 2.2.8. Cell Cycle Experiment of HeLa/DDP Cells

**Scheme 9:**
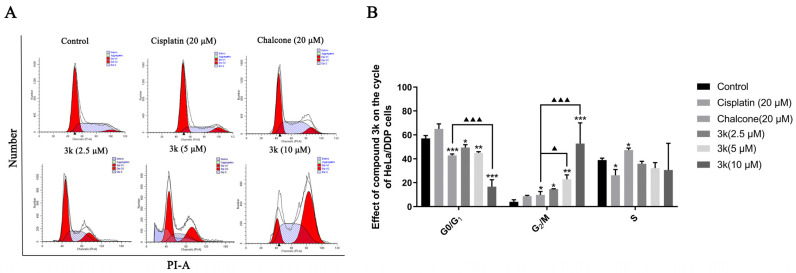
(**A**) Cell cycle effects of **3k** and cisplatin on HeLa/DDP. (**B**) Quantitative analysis of the effect of **3k** on the HeLa cell cycle in the bar graph. All the above results were expressed as the mean ± SD of three independent experiments: * *p* < 0.05, ** *p* < 0.01, *** *p* < 0.001 compared with the control group; ▲ *p* < 0.05, ▲▲▲ *p* < 0.001 compared with the chalcone group.

The findings of this study indicate that compound **3k** exhibited notable efficacy in inhibiting the progression of HeLa/DDP cells into the G2/M phase. Concentrations of 2.5, 5, and 10 μM of compound **3k** resulted in blocking rates of 14.71%, 22.85%, and 52.72%, respectively. These rates were significantly higher compared to the control group, which had a blocking rate of 4.05% (*p* < 0.05, *p* < 0.01, and *p* < 0.001, respectively). Furthermore, at concentrations of 5 and 10 μM, compound **3k** demonstrated significantly higher blocking rates at the G2/M phase of HeLa/DDP cells compared to the chalcone group (*p* < 0.05 and *p* < 0.001, respectively). Furthermore, the inhibitory effect of cisplatin on HeLa/DDP cells in the G0/G1 phase exhibited a significant increase, rising from 57.03% to 65.07%, when compared to the blank control group. However, this increase was notably lower than the inhibitory effect of cisplatin on HeLa cells in the G0/G1 phase, which saw a rise from 52.1% to 73.93% (*p* < 0.05) (Scheme 9A,B).

The aforementioned findings demonstrate that compound **3k** exhibited a concentration-dependent inhibition of HeLa/DDP cells in the G2/M phase, consistent with the outcomes observed in HeLa cells. The findings of this study indicate that the presence of P-gp protein may not have an impact on the effectiveness of **3k** in impeding the progression of HeLa and HeLa/DDP cells during the G2/M phase. Prior investigations have provided evidence that co-administration of cisplatin at concentrations of 0.25, 0.5, and 1 μM significantly enhances the inhibitory effects of **3k** on the proliferation of HeLa/DDP cells. In contrast, the Western blot experiments revealed no statistically significant variation in the expression of **3k** on P-gp within the aforementioned three concentration ranges when compared to the blank and verapamil groups. This suggests that **3k** may circumvent the exocytosis function of P-gp, potentially attributed to its inhibitory impact on the active effect of P-gp. In addition, the results demonstrate that the inhibition rates of cisplatin in HeLa and HeLa/DDP cells during the G0/G1 phase were 73.93% and 65.07%, respectively. This suggests that the capacity of cisplatin to block HeLa/DDP cells during the G0/G1 phase was reduced, potentially due to the emergence of cisplatin resistance in HeLa/DDP cells.

#### 2.2.9. Migration and Invasion of HeLa/DDP Cells

**Scheme 10:**
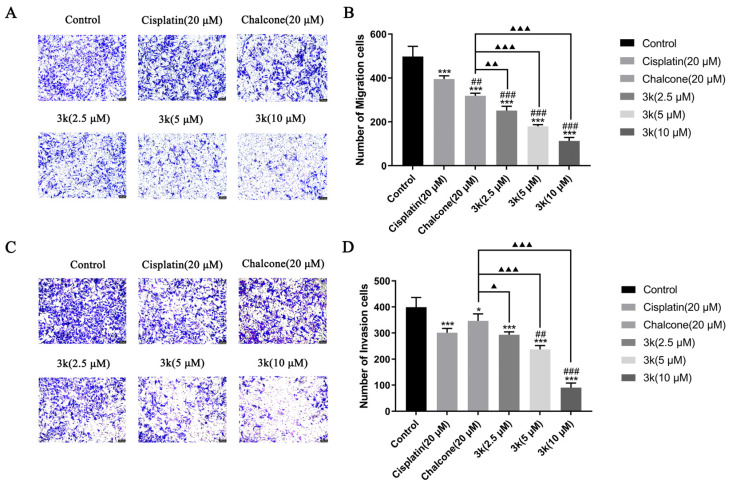
(**A**) Morphology of migrated cells 24 h after compound **3k** was applied to HeLa/DDP cells in the Transwell migration assay. (**B**) Quantitative analysis of the number of migrated cells. (**C**) Morphology of invaded cells 24 h after compound **3k** was applied to HeLa/DDP cells in the Transwell invasion assay. (**D**) Quantitative analysis of the number of invaded cells; HeLa cell morphology was observed under an inverted microscope (magnification: ×100). All the above results were expressed as the mean ± SD of three independent experiments: * *p* < 0.05, *** *p* < 0.001 compared with the control group; ## *p* < 0.01, ### *p* < 0.001 compared with the cisplatin group; ▲ *p* < 0.05, ▲▲ *p* < 0.01, ▲▲▲ *p* < 0.001 compared with the chalcone group.

The findings of the study indicate that compound **3k** exhibited a significant inhibitory effect on the penetration of HeLa/DDP cells through the chamber membrane. The number of migrated cells decreased from 497 in the control group to 251, 179, and 113 at concentrations of 2.5, 5, and 10 μM of **3k**, respectively. These values were significantly lower than those observed in the control group and cisplatin group (395) (*p* < 0.001). Furthermore, the number of migrated cells was also lower than that observed in the chalcone group (319) at concentrations of 2.5, 5, and 10 μM of **3k**, with statistical differences of p < 0.01, *p* < 0.001, and *p* < 0.001, respectively (Scheme 10A,B).

The number of invasion cells decreased from 399 in the control group to 293, 237, and 90 at concentrations of 2.5, 5, and 10 μM of **3k**, respectively. The presence of **3k** resulted in a significant decrease in the number of cells undergoing invasion compared to the control group (*p* < 0.001). Moreover, the number of cells undergoing invasion was significantly lower than that in the chalcone group (347) at the same concentrations of **3k**, with statistically significant differences of *p* < 0.05, *p* < 0.001, and *p* < 0.001). Additionally, when exposed to a concentration of 5 and 10 μM, the number of cells undergoing invasion at **3k** was significantly lower compared to the cisplatin group (301) (*p* < 0.01 and *p* < 0.001) (Scheme 10C,D).

The etiology of cervical cancer is multifaceted and intricate, with its pathogenesis primarily marked by regional dissemination, and prompt surgical intervention is often precluded by premature invasive metastasis [34]. Compound **3k** demonstrated a dose-dependent suppression of HeLa/DDP cell invasion and migration, surpassing the inhibitory effects of cisplatin and chalcone on HeLa/DDP cells within the concentrations of 2.5, 5, and 10 μM. These findings indicate that **3k** possesses the ability to mitigate the invasive and migratory properties of tumor cells. Furthermore, the inhibitory efficacy of cisplatin on the invasion and migration of HeLa/DDP cells was notably diminished compared to that of the parental HeLa cells. This disparity could potentially be attributed to the heightened expression of P-gp in HeLa/DDP cells, leading to the inadequate accumulation of cisplatin within these cells. Consequently, this diminished accumulation of cisplatin in HeLa/DDP cells subsequently diminishes its inhibitory potential toward the invasion and migration of these cells.

#### 2.2.10. Molecular Docking

The results of the molecular docking analysis revealed that the chalcone and compound **3k** exhibited binding energies of −6.4 and −7.1 kcal-mol^−1^, respectively, toward the colchicine site of microtubule proteins. Additionally, the co-crystalline ligand CN2 demonstrated a binding energy of −8.8 kcal-mol^−1^. Furthermore, it is noteworthy that the binding energies of chalcone and compound **3k** to the active pocket of P-gp were determined to be −8.5 and −7.4 kcal-mol^−1^, respectively, while the co-crystalline ligand, UIC2-Fab, exhibited a binding energy of −10.8 kcal-mol^−1^ (Table 3).

**Scheme 11:**
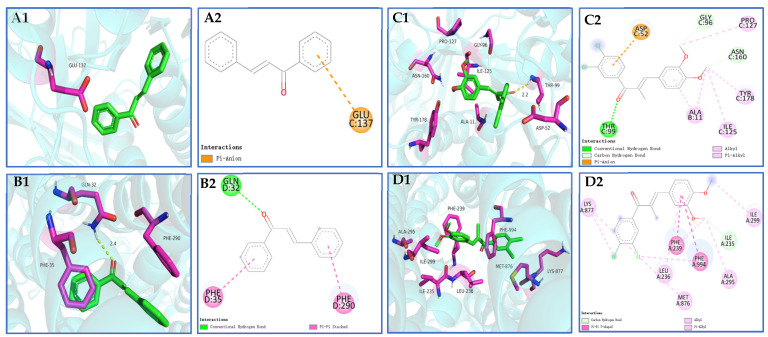
(**A1**,**A2**,**B1**,**B2**) 3D schematic of molecular docking of the pilot compound chalcone with tubulin and P-gp proteins; (**C1**,**C2**,**D1**,**D2**) 3D schematic of compound 3k docked with tubulin and P-gp protein molecules.

Compound **3k** exhibits the ability to engage in hydrogen bonding interactions with amino acid residue THR-99 (2.2 Å) at the colchicine binding site of tubulin. Additionally, it forms pi–anion bonds with ASP-52 and establishes hydrophobic solid interactions with GLY-96, PRO-127, ASN-160, TYR-178, ALA-11, and ILE-125 hydrophobic interactions (Scheme 11(C1,C2)). Compound **3k** exhibits alkyl and pi–pi T-type interactions with specific amino acid residues (LYS-877, LEU-236, MET-876, ALA-295, and ILE-299) at the binding site of P-gp. Additionally, it forms pi–pi T-type interactions with PHE-239 and PHE-994. These interactions contribute to the establishment of robust hydrophobic interactions, potentially enhancing the binding stability of compound **3k** to P-gp (Scheme 11(D1,D2)).

A widely used open-source program for molecular docking, AutoDock Vina, has a reputation for speed and efficiency [35]. The incorporation of 3,4-Cl and 3,4-OCH_3_ substituents into the original chalcone framework led to the formation of compound **3k**, which exhibited diverse interactions with numerous amino acid residues surrounding the tubulin, as well as hydrogen bonding interactions with THR-99 (2.2Å). This suggests that the incorporation of 3,4-Cl and 3,4-OCH_3_ substituents on the A and B rings of the chalcone parent nucleus greatly improved the binding affinity and stability of the compound to tubulin. In the docking experiments involving compound **3k** and P-gp, it was observed that the incorporation of 3,4-Cl and 3,4-OCH_3_ substituents facilitated the binding of **3k** to a greater number of amino acid residues located in the peripheral region of the P-gp target site. This interaction also resulted in the establishment of a robust hydrophobic force, potentially augmenting the stability of the binding.

#### 2.2.11. Immunofluorescence Assay

(1) HeLa cell immunofluorescence assay

**Scheme 12:**
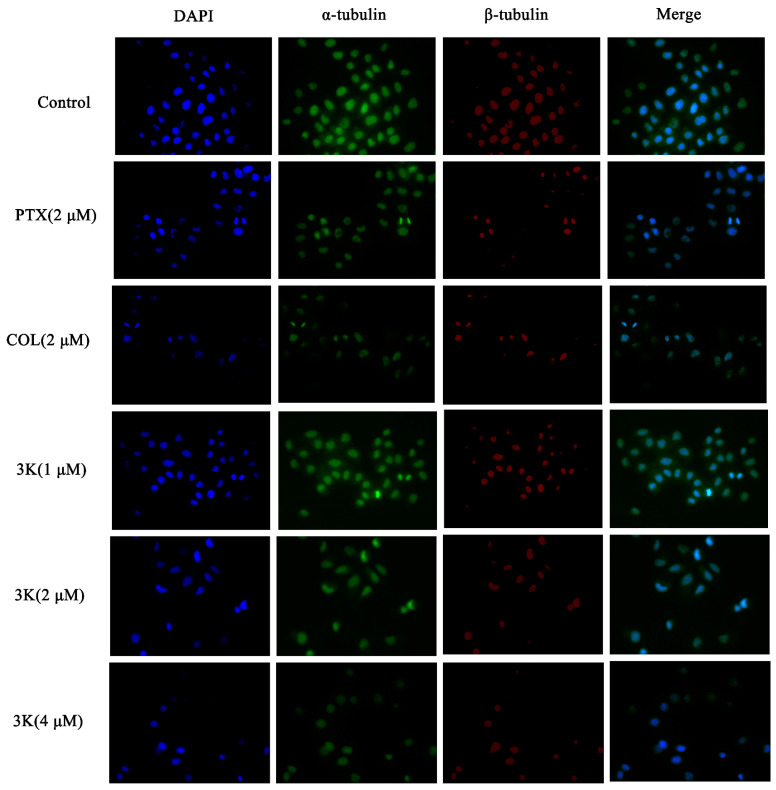
Effect of compound **3k** on microtubulin in HeLa cells (blue fluorescence for nucleus, green fluorescence for α-tubulin, red fluorescence for β-tubulin; ×400).

The findings indicated that within the control group, HeLa cells exhibited consistent size and regular morphology, characterized by distinct and intact borders with visible contours, while maintaining their original cellular structure. In addition, the nuclei were enveloped in robust microtubule fluorescence. Following exposure to compound **3k** at concentrations of 1, 2, and 4 μM, as well as colchicine (COL), a substantial decrease in cell count was observed, and the green and red fluorescence of the encapsulated cells weakened. Furthermore, there was a notable alteration in cell morphology, as cells appeared diminished in size and ruptured, while the nuclei exhibited coalescence and solidification. Following paclitaxel (PTX) treatment, there was a reduction in cell count; however, the cellular morphology remained relatively intact. Additionally, the fluorescence intensity of microtubules surrounding the cells decreased or vanished. Similarly, **3k**-treated cells exhibited alterations in microtubule morphology comparable to the COL-treated group, which differed from the PTX-treated group. Moreover, the extent of microtubule interference intensified with escalating drug concentration, leading to a decrease in cell count and a gradual decline in fluorescence intensity (Scheme 12).

**Scheme 13:**
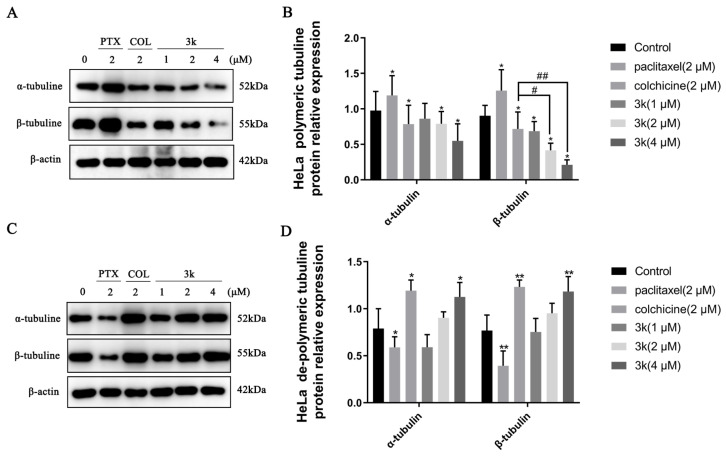
(**A**) Expression level of α-/β-tubulins in polymerized state in HeLa cells. (**B**) Histogram of microtubulin expression level (*n* = 3). (**C**) Expression level of α-/β-tubulins in dissociated state in HeLa cells. (**D**) Histogram of microtubulin expression level (*n* = 3). All the above results were expressed as the mean ± SD of three independent experiments: * *p* < 0.05, ** *p* < 0.01, compared with the control group; # *p* < 0.05, ## *p* < 0.01, compared with the colchicine group.

The Western blotting results indicated a significant decrease (*p* < 0.05) in the expression of α and β microtubule proteins in the polymerized state after HeLa cells were exposed for 24 h to **3k** at concentrations of 2 and 4 μM, compared to the control group. Furthermore, the positive drug colchicine also exhibited a decrease in the expression of α and β microtubule proteins in the polymerized state (*p* < 0.05). Conversely, the positive drug paclitaxel significantly enhanced the expression of polymerized state α and β microtubule proteins (*p* < 0.05). In addition, **3k** was able to significantly reduce the expression of polymerized β-microtubule proteins at 2 and 4 μM concentrations compared to the colchicine group (*p* < 0.05 and *p* < 0.01) (Scheme 13A,B). A significant increase in the expression of dissociated state α and β microtubule proteins was observed after treatment of HeLa cells with 4 μM of **3k** for 24 h (*p* < 0.05 and *p* < 0.01). In comparison to the blank control group, the expression of dissociated α and β microtubule proteins was significantly increased at a concentration of 2 μM (*p* < 0.05 and *p* < 0.001) by the positive drug colchicine. In contrast, paclitaxel significantly decreased the expression of dissociated state α and β microtubule proteins (*p* < 0.05 and *p* < 0.01) (Scheme 13C,D).

(2) HeLa/DDP cell immunofluorescence assay

**Scheme 14:**
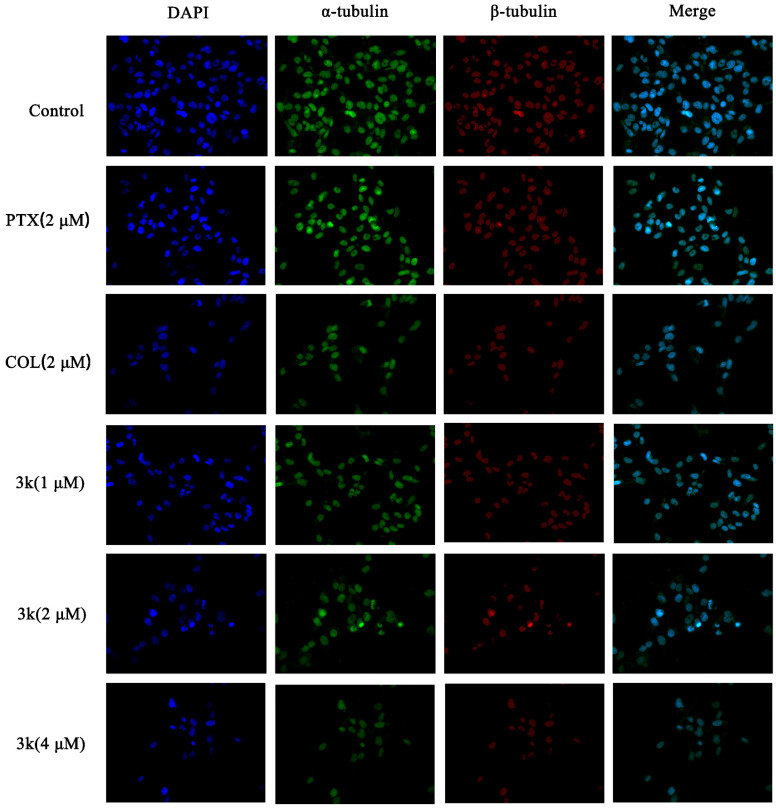
Effect of compound **3k** on microtubulin in HeLa/DDP cells (blue fluorescence for nucleus, green fluorescence for α-tubulin, red fluorescence for β-tubulin; ×400).

The immunofluorescence findings demonstrated a progressive decline in the quantity of HeLa/DDP cells and an attenuation of the green and red fluorescence of the enclosed cells following a 24 h exposure of HeLa/DDP cells to Compound **3k** at concentrations of 1, 2, and 4 μM, when compared to the control group. Furthermore, notable alterations in cellular morphology were observed, including reductions in cell size, cellular death and rupture, as well as the merging and solidification of cellular nuclei. Following treatment with paclitaxel, a discernible decrease in cell count was observed. Nevertheless, the cellular structure remained relatively unaltered, and there was no significant alteration in the fluorescence intensity of the microtubules surrounding the cells. Likewise, the alterations in microtubule morphology in the **3k**-treated cells resembled those in the colchicine-treated group, but differed from those in the paclitaxel-treated group (Scheme 14).

**Scheme 15:**
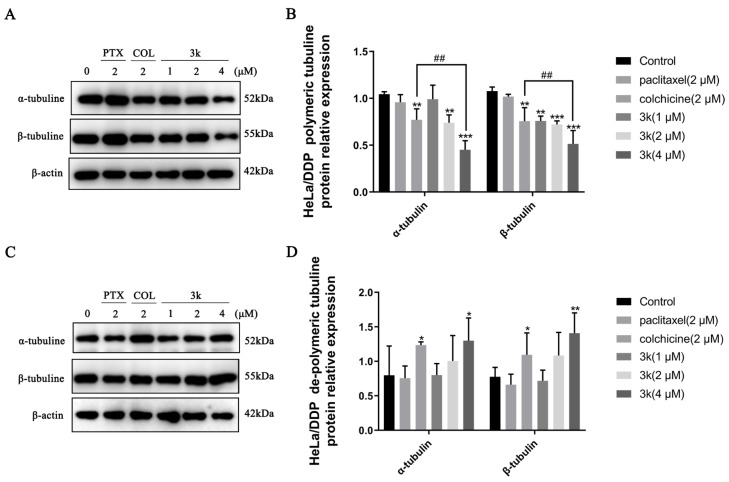
(**A**) Expression level of α-/β-tubulins in polymerized state in HeLa/DDP cells. (**B**) Histogram of microtubulin expression level (*n* = 3). (**C**) Expression level of α-/β-tubulins in dissociated state in HeLa/DDP cells. (**D**) Histogram of microtubulin expression level (*n* = 3). All the above results were expressed as the mean ± SD of three independent experiments: * *p* < 0.05, ** *p* < 0.01, *** *p* < 0.001 compared with the control group; ## *p* < 0.01 compared with the colchicine group.

The Western blot analysis demonstrated that the presence of compound **3k** at concentrations of 2 and 4 μM resulted in a significant decrease in the expression of polymerized α-microtubulin in HeLa/DDP cells after a 24 h treatment when compared to the control group (*p* < 0.01 and *p* < 0.001). Also, the expression of polymerized α-microtubulin at 4 μM was significantly lower than that observed in the colchicine group (*p* < 0.01). At concentrations of 1, 2, and 4 μM, the compound **3k** demonstrated a significant reduction in the expression of polymerized β microtubulin (*p* < 0.01, *p* < 0.001, and *p* < 0.001). Moreover, at a concentration of 4 μM, the efficacy of **3k** in reducing the expression of polymerized β microtubulin was notably superior to that of the colchicine group (*p* < 0.01). Furthermore, the group treated with colchicine exhibited a significant decrease in the expression of polymerized state α and β microtubule proteins when compared to the control group (*p* < 0.01). Conversely, the administration of paclitaxel did not yield a significant impact on the expression of α and β microtubule proteins in their polymerized state (*p* > 0.05) (Scheme 15A,B).

At a concentration of 4 μM, the administration of **3k** resulted in a significant increase in the expression of dissociated state α and β microtubule proteins in HeLa/DDP cells compared to the control group (*p* < 0.05 and *p* < 0.01). Similarly, the colchicine group exhibited an increase in the expression of dissociated state α and β microtubule proteins compared to the control group (*p* < 0.05). Conversely, the administration of paclitaxel had minimal impact on the expression of dissociated state α and β microtubule proteins (*p* > 0.05) (Scheme 15C,D).

Immunofluorescence experiments revealed that Compound **3k**, when administered at concentrations of 2 and 4 μM, exhibited a significant reduction in the intensity of green and red fluorescence in the periphery of HeLa and HeLa/DDP cells. Furthermore, this compound led to a decrease in cell size, cell death, rupture, nucleus merging, and coagulation. These effects were comparable to those observed in the colchicine-treated group, an inhibitor of microtubule protein polymerization, but differed from the paclitaxel-treated group, a promoter of microtubule protein polymerization. It is noteworthy that the impact of paclitaxel on HeLa/DDP cells was considerably less pronounced compared to its effect on HeLa cells. This disparity primarily encompassed alterations in cell morphology, the extent of cell number reduction, and modifications in the peripheral fluorescence intensity of the cells. This discrepancy was attributed to the heightened expression of P-gp in HeLa/DDP cells resulting in the exocytosis of paclitaxel by P-gp and consequently diminishing the efficacy of paclitaxel. The expression of polymerized α and β microtubule proteins and free α and β microtubule proteins in HeLa and HeLa/DDP cells was investigated further using Western blotting experiments. It was found that concentrations of 2 and 4 μM of **3k** significantly decreased the expression of polymerized α and β microtubule proteins and increased the expression of free α and β microtubule proteins. Conversely, paclitaxel did not show a significant difference in the expression of polymerized and free-state microtubule proteins in HeLa/DDP cells compared to the control group. This lack of difference may be attributed to the high expression of P-gp in HeLa/DDP cells, which leads to the exocytosis of paclitaxel by P-gp.

Microtubulin, a protein family comprising multiple members, serves as the fundamental structural component of microtubules. Among the identified isoforms, namely α, β, γ, δ, ε, ζ, and η, α and β microtubulin are the most prevalent [36]. The assembly of these two microtubulin variants into heterodimers occurs within a dynamic equilibrium of polymerization and dissociation [37]. The proper progression of mitosis relies on the intricate interplay between intracellular microtubule polymerization and depolymerization transitions [38,39]. In this experimental study, paclitaxel (PTX) and colchicine (COL) were employed as positive controls to investigate their respective mechanisms of action, which involve the promotion and inhibition of microtubule protein polymerization. Both PTX and COL target microtubule proteins, with PTX promoting aggregation and COL inhibiting aggregation. The selection of PTX and COL as positive and negative positive controls, respectively, enhances the elucidation of the mechanism of action of **3k**. The observed alterations in cell microtubules’ morphology following **3k** treatment exhibited similarities to the COL-treated group, while differing from the paclitaxel-treated group. Moreover, the extent of microtubule disruption escalated with higher drug concentrations, implying that the mechanism of action of **3k** on microtubules may resemble that of COL. Specifically, it involves the inhibition of microtubule aggregation, leading to the disruption of microtubule structure. A functional and undamaged microtubule architecture is imperative for the accurate replication and proliferation of cells. Disruptions to the dynamics of microtubules or a decrease in their polymerization state typically result in the halting of cellular progression at the G2/M phase [40]. Compound **3k** was observed to induce cell cycle arrest in HeLa and HeLa/DDP cells at the G2/M phase during cycling experiments, potentially attributable to its ability to impede microtubule protein polymerization.

## 3. Material and Methods

### 3.1. Chemistry

All solvents and reagents used in this study were acquired from commercial suppliers and were used without further purification, including anhydrous ethanol (Fuyu Fine Chemical Co., Ltd., Tianjin, China). Melting points were measured using a WRX-4 micromelting point meter. The microwave reactor model used was a WBFY-205 (YU HUA INSTRUMENT, Shanghai, China). Column chromatography silica gel particle sizes of 200 or 400 mesh were purchased from Qingdao Chemical Co. (Qingdao, China), and thin-layer chromatography (TLC) analysis was performed using silica gel 60 F254 analytical plates (Merck, Billerica, MA, USA). The reaction products were purified via crystallization or flash column chromatography using a mixture of petroleum ether and ethyl acetate as the eluent. ^1^H NMR and ^13^C NMR spectra were measured on a Bruker Avance III 400 HD spectrometer (Bruker Bioscience, Billerica, MA, USA), using tetramethylsilane (TMS) as an internal standard. Chemical shifts (δ) are reported in parts per million downfield relative to tetramethylsilane. A Thermo Scientific Q Exactive (Thermo Scientific, Waltham, MA, USA) mass spectrometer was also used. Using an Agilent 1220 high-performance liquid chromatograph (Agilent Technologies Co., Ltd., Santa Clara, CA, USA), the purity of all tested compounds was ≥95% with a detection wavelength of 372 nm, mobile phase (MeOH:H_2_O = 70:30), and flow rate of 1 mL/min.

### 3.2. Synthesis and Structural Characterization

The raw materials, consisting of 1.12 g (5.0 mmol) of reactive **1a** and 1.18 g (6.0 mmol) of **2a**, were introduced into a 100 mL round-bottom flask. Subsequently, 30 mL of anhydrous ethanol was added to the flask. The resulting solution was dissolved using ultrasound, followed by the gradual addition of 10 mL of 20% KOH with continuous stirring. To facilitate the reaction, a condensate tube was connected to a microwave reaction instrument, with the reaction power set at 100 W. The reaction progress was observed through the utilization of thin-layer chromatography (TLC). The solvent ratio employed for the TLC analysis was PE:EA = 2:1, resulting in an Rf value of 0.4. Additionally, the reaction was conducted under microwave irradiation for a duration of 5 min. The crude product was then isolated and purified via silica gel column chromatography, utilizing a petroleum ether-ethyl acetate mixture with a volume ratio of 5:1. Compound **3a** was obtained by collecting the yellow liquid fraction, concentrating it under reduced pressure, dissolving it in an appropriate quantity of anhydrous ethanol, and subsequently recrystallizing it. The synthesis of compounds **3b**–**3p** followed the identical procedure. The α-methyl chalcone derivatives **3a**–**3p** are all E isomers, the same isomers reported in the literature for α-methyl chalcone-containing derivatives [24,26]. The mass of the data given by the HMRS for chlorinated compounds is the mass of the ^35^Cl isotope.

#### 3.2.1. (*E*)-1-(3,4-Dichlorophenyl)-2-methyl-3-(2,4,5-trimethoxyphenyl)prop-2-en-1-one(**3a**)

Synthesized from 3,4-dichlorophenone and 2,4,5-trimethoxybenzaldehyde, the reaction time is about 3 min, yellow solid, yield 22%, m.p. 129.6~130.5 °C; ^1^H NMR (400 MHz, Chloroform-d) δ 7.85 (d, *J* = 2.3 Hz, 1H), 7.58 (dd, *J* = 8.6, 2.3 Hz, 1H), 7.52 (d, *J* = 8.3, 1H), 7.40 (q, *J* = 1.4 Hz, 1H), 7.01 (s, 1H), 6.52 (s, 1H), 3.90–3.80 (m, 9H), 2.21 (s, 3H).^13^C NMR (101 MHz, Chloroform-d) δ 196.75, 153.08, 151.11, 142.57, 139.30, 138.66, 135.69, 133.89, 132.46, 131.64, 130.20, 128.75, 115.73, 113.56, 96.64, 56.72, 56.31, 56.09, 14.20. HRMS (ESI) *m*/*z* calcd. for C_19_H_19_Cl_2_O_4_^+^ (M + H)^+^ 381.06549, found 381.06589.

#### 3.2.2. (*E*)-1-(3,4-Dichlorophenyl)-2-methyl-3-(3,4,5-trimethoxyphenyl)prop-2-en-1-One(**3b**)

Synthesized from 3,4-dichlorophenone and 3,4,5-trimethoxybenzaldehyde, the reaction time is about 3 min, yellow oil, yield 22%; ^1^H NMR (400 MHz, Chloroform-d) δ7.81 (d, *J* = 2.0 Hz, 1H), 7.65 (dd, *J* = 8.6, 2.2 Hz, 1H), 7.55 (d, *J* = 8.5 Hz, 1H), 7.10 (d, *J* = 1.7 Hz, 1H), 6.75 (s, 2H), 3.93 (s, 6H), 3.88 (s, 3H), 3.69 (s, 3H).^13^C NMR (101 MHz, Chloroform-d) δ 191.01, 153.12(2C), 138.92, 138.32, 135.94, 135.60, 134.83, 131.18, 130.72, 130.31, 128.50, 127.92, 106.67(2C), 61.34, 56.16, 56.03, 15.18. HRMS (ESI) *m*/*z* calcd for C_19_H_19_Cl_2_O_4_^+^ (M + H)^+^ 381.06549, found 381.06577.

#### 3.2.3. (*E*)-1-(3,4-Dichlorophenyl)-2-methyl-3-(2,3,4-trimethoxyphenyl)prop-2-en-1-one(**3c**)

Synthesized from 3,4-dichlorophenone and 2,3,4-trimethoxybenzaldehyde, the reaction time is about 2.5 min, yellow oil, yield 22%; m.p. 114.1~115.5 °C; ^1^H NMR (400 MHz, Chloroform-d) δ 7.85 (d, *J* = 2.0 Hz, 1H), 7.59 (dd, *J* = 8.6, 2.2 Hz, 1H), 7.54 (d, *J* = 8.2 Hz, 1H), 7.35 (s, 1H), 7.19 (d, *J* = 8.7 Hz, 1H), 6.74 (d, *J* = 8.7 Hz, 1H), 3.91 (s, 3H), 3.87 (s, 3H), 3.84 (s, 3H), 2.20 (s, 3H).^13^C NMR (101 MHz, Chloroform-d) δ 196.82, 154.77, 152.69, 142.22, 138.82, 138.44, 135.89, 135.03, 132.56, 131.51, 130.29, 128.66, 124.82, 122.26, 107.01, 61.37, 60.95, 56.08, 14.18. HRMS (ESI) *m*/*z* calcd for C_19_H_19_Cl_2_O_4_^+^ (M + H)^+^ 381.06549, found 381.06586.

#### 3.2.4. (*E*)-1-(3,4-Dichlorophenyl)-2-methyl-3-(2,4,6-trimethoxyphenyl)prop-2-en-1-one(**3d**)

Synthesized from 3,4-dichlorophenone and 2,4,6-trimethoxybenzaldehyde, the reaction time is about 6 min, yellow solid, yield 89%, m.p. 67.1~67.8 °C; ^1^H NMR (400 MHz, Chloroform-d) δ 7.92 (d, *J* = 2.0 Hz, 1H), 7.64 (dt, *J* = 8.3, 1.3 Hz, 1H), 7.51 (d, *J* = 8.3 Hz, 1H), 7.06–7.00 (m, 1H), 6.16 (s, 2H), 3.85(s, 3H), 3.82 (s, 6H), 1.87 (s, 3H).^13^C NMR (101 MHz, Chloroform-d) δ 196.79, 162.23, 158.43(2C), 138.74, 137.43, 137.37, 135.67, 132.26, 131.93, 130.16, 128.90, 106.09, 90.34(2C), 55.60(2C), 55.42, 15.14. HRMS (ESI) *m*/*z* calcd for C_19_H_19_Cl_2_O_4_^+^ (M + H)^+^ 381.06549, found 381.06580.

#### 3.2.5. (*E*)-1-(2,4-dichlorophenyl)-2-methyl-3-(2,4,6-trimethoxyphenyl)prop-2-en-1-one(**3e**)

Synthesized from 2,4-dichlorophenone and 2,4,6-trimethoxybenzaldehyde, the reaction time is about 6 min, yellow solid, yield 94%, m.p. 104.6~105.7 °C; ^1^H NMR (400 MHz, Chloroform-d) δ 7.43 (q, *J* = 1.6 Hz, 1H), 7.35–7.24 (m, 2H), 6.99 (d, *J* = 1.4 Hz, 1H), 6.12 (s, 2H), 3.83 (s, 3H), 3.77 (s, 6H), 1.86 (s, 3H).^13^C NMR (101 MHz, Chloroform-d) δ 196.88, 162.45, 158.57(2C), 139.90, 138.55, 138.31, 135.44, 132.35, 130.04, 129.70, 126.64, 106.19, 90.41, 55.62(2C), 55.39, 14.05. HRMS (ESI) *m*/*z* calcd for C_19_H_19_Cl_2_O_4_^+^ (M + H)^+^ 381.06549, found 381.06583.

#### 3.2.6. (*E*)-1-(3,4-dichlorophenyl)-3-(2,4-dimethoxyphenyl)-2-methylprop-2-en-1-one(**3f**)

Synthesized from 3,4-dichlorophenone and 2,4-dimethoxybenzaldehyde, the reaction time is about 6 min, yellow solid, yield 48%, m.p. 112.3~113.6 °C; ^1^H NMR (400 MHz, Chloroform-d) δ 7.85 (d, *J* = 1.4 Hz, 1H), 7.58 (dt, *J* = 8.2, 1.4 Hz, 1H), 7.51 (d, *J* = 8.3 Hz, 1H), 7.42–7.35 (m, 2H), 6.55 (dd, J = 8.7, 2.4 Hz, 1H), 6.46 (d, J = 2.4 Hz, 1H), 3.85 (s, 3H), 3.80 (s, 3H), 2.18 (s, 3H).^13^C NMR (101 MHz, Chloroform-d) δ 196.85, 161.95, 159.09, 139.40, 138.71, 135.65, 133.95, 132.44, 131.68, 130.94, 130.18, 128.77, 117.27, 104.37, 98.24, 55.56, 55.48, 14.15. HRMS (ESI) *m*/*z* calcd for C_18_H_17_Cl_2_O_3_^+^ (M + H)^+^ 351.05493, found 351.05518.

#### 3.2.7. (*E*)-1-(3,4-dichlorophenyl)-3-(2,6-dimethoxyphenyl)-2-methylprop-2-en-1-one(**3g**)

Synthesized from 3,4-dichlorophenone and 2,6-dimethoxybenzaldehyde, the reaction time is about 7 min, white solid, yield 69%, m.p. 134.4~135.6 °C; ^1^H NMR (400 MHz, Chloroform-d) δ 7.97 (d, *J* = 1.8 Hz, 1H), 7.67 (dt, *J* = 8.2, 1.8 Hz, 1H), 7.52 (dd, *J* = 8.2, 1.7 Hz, 1H), 7.29(t, J = 8.3 Hz, 1H), 7.04 (d, J = 1.7 Hz, 1H), 6.59 (d, J = 8.4, 2H), 3.84 (s, 6H), 1.87 (s, 3H).^13^C NMR (101 MHz, Chloroform-d) δ 196.67, 157.54(2C), 138.43, 138.25, 136.90, 135.92, 132.34, 132.05, 130.23(2C), 128.95, 113.09, 103.55(2C), 55.67(2C), 15.06. HRMS (ESI) *m*/*z* calcd for C_18_H_17_Cl_2_O_3_^+^ (M + H)^+^ 351.05493, found 351.05524.

#### 3.2.8. (*E*)-1-(3,4-Dichlorophenyl)-3-(3,5-dimethoxyphenyl)-2-methylprop-2-en-1-one(**3h**)

Synthesized from 3,4-dichlorophenone and 3, 5-dimethoxybenzaldehyde, the reaction time is about 6 min, white solid, yield 28%, m.p. 157.0~158.1 °C; ^1^H NMR (400 MHz, Chloroform-d) δ 8.12 (d, *J* = 2.1 Hz, 1H), 7.92 (dd, *J* = 8.4, 2.0 Hz, 1H), 7.62 (d, *J* = 8.4 Hz, 1H), 7.28 (dd, *J* = 2.0, 1.2 Hz, 1H), 6.34 (d, *J* = 2.2 Hz, 2H), 6.06 (t, *J* = 2.2 Hz, 1H), 3.71 (s, 6H), 1.03 (s, 3H).^13^C NMR (101 MHz, Chloroform-d) δ 200.59, 160.60(2C), 139.59, 137.88, 135.99(2C), 133.64, 130.99, 130.53, 127.50(2C), 107.50(2C), 98.64, 55.22(2C), 14.79. HRMS (ESI) *m*/*z* calcd for C_18_H_17_Cl_2_O_3_^+^ (M + H)^+^ 351.05493, found 351.05493.

#### 3.2.9. (*E*)-1-(3,4-Dichlorophenyl)-3-(2,3-dimethoxyphenyl)-2-methylprop-2-en-1-one(**3i**)

Synthesized from 3,4-dichlorophenone and 2, 3-dimethoxybenzaldehyde, the reaction time is about 5.5 min, yellow solid, yield 34%, m.p. 99.8~100.7 °C; ^1^H NMR (400 MHz, Chloroform-d) δ 7.90 (d, *J* = 2.0 Hz, 1H), 7.64 (dd, *J* = 8.2, 2.0 Hz, 1H), 7.54 (d, *J* = 8.2 Hz, 1H), 7.35 (s, 1H), 7.11 (t, *J* = 8.0 Hz, 1H), 7.04–6.98 (m, 1H), 6.95 (dd, *J* = 8.1, 1.6 Hz, 1H), 3.88 (s, 3H), 3.79 (s, 3H), 2.16 (s, 3H).^13^C NMR (101 MHz, Chloroform-d) δ 196.70, 152.81, 147.57, 138.65, 138.02, 136.78, 136.23, 132.66, 131.67, 130.35, 129.74, 128.78, 123.85, 121.62, 113.19, 60.97, 55.88, 14.26. HRMS (ESI) *m*/*z* calcd for C_18_H_17_Cl_2_O_3_^+^ (M + H)^+^ 351.05493, found 351.05490.

#### 3.2.10. (*E*)-1-(3,4-Dichlorophenyl)-3-(2,5-dimethoxyphenyl)-2-methylprop-2-en-1-one(**3j**)

Synthesized from 3,4-dichlorophenone and 2,5-dimethoxybenzaldehyde, the reaction time is about 5.5 min, yellow oil, yield 61%, ^1^H NMR (400 MHz, Chloroform-d) δ 7.90 (d, *J* = 2.0 Hz, 1H), 7.63 (dd, *J* = 8.3, 2.0 Hz, 1H), 7.54 (d, *J* = 8.3 Hz, 1H), 7.33 (s, 1H), 6.96 (d, *J* = 3.0 Hz, 1H), 6.89 (dd, *J* = 8.9, 3.0 Hz, 1H), 6.84 (d, *J* = 8.9 Hz, 1H), 3.80 (s, 3H), 3.79 (s, 3H), 2.18 (s, 3H).^13^C NMR (101 MHz, Chloroform-d) δ 196.74, 153.06, 151.90, 139.01, 138.17, 136.08, 135.96, 132.57, 131.82, 130.29, 128.83, 125.08, 115.89, 114.91, 111.41, 56.00, 55.86, 14.15. HRMS (ESI) *m*/*z* calcd for C_18_H_17_Cl_2_O_3_^+^ (M + H)^+^ 351.05493, found 351.05496.

#### 3.2.11. (*E*)-1-(3,4-Dichlorophenyl)-3-(3,4-dimethoxyphenyl)-2-methylprop-2-en-1-one(**3k**)

Synthesized from 3,4-dichlorophenone and 3,4-dimethoxybenzaldehyde, the reaction time is about 4.5 min, yellow oil, yield 42%, ^1^H NMR (400 MHz, Chloroform-d) δ 7.79 (d, *J* = 2.3 Hz, 1H), 7.62 (dd, *J* = 8.6, 2.2 Hz, 1H), 7.52 (q, *J* = 1.5 Hz, 1H), 7.40 (d, *J* = 8.5 Hz, 1H), 7.25 (dd, *J* = 8.8, 2.0 Hz, 1H), 7.11 (d, *J* = 1.8 Hz, 1H), 6.93 (d, *J* = 8.6 Hz, 1H), 3.92 (s, 3H), 3.90 (s, 3H), 2.26 (s, 3H).^13^C NMR (101 MHz, Chloroform-d) δ 196.88, 159.75, 158.50, 143.59, 138.74, 135.71, 134.13, 131.83, 131.22, 130.25, 129.29, 128.49, 127.75, 114.59, 114.36, 63.62(2C), 14.28. HRMS (ESI) *m*/*z* calcd for C_18_H_17_Cl_2_O_3_^+^ (M + H)^+^ 351.05493, found 351.05490.

#### 3.2.12. (*E*)-1-(3,4-Dichlorophenyl)-3-(2-methoxyphenyl)-2-methylprop-2-en-1-one(**3l**)

Synthesized from 3,4-dichlorophenone and 2-methoxybenzaldehyde, the reaction time is about 7 min, yellow solid, yield 64%, m.p. 92.8~93.6 °C; ^1^H NMR (400 MHz, Chloroform-d) δ 7.90 (d, *J* = 2.0 Hz, 1H), 7.63 (dd, *J* = 8.3, 2.0 Hz, 1H), 7.53 (d, *J* = 8.3 Hz, 1H), 7.43–7.30 (m, 3H), 7.01 (t, *J* = 7.5 Hz, 1H), 6.91 (d, *J* = 8.3 Hz, 1H), 3.83 (s, 3H), 2.16 (s, 3H).^13^C NMR (101 MHz, Chloroform-d) δ 196.79, 157.51, 139.34, 138.28, 136.01, 135.69, 132.54, 131.82, 130.43, 130.28, 129.95, 128.84, 124.38, 120.22, 110.52, 55.50, 14.11. HRMS (ESI) *m*/*z* calcd for C_17_H_15_Cl_2_O_2_^+^ (M + H)^+^ 321.04436, found 321.04437.

#### 3.2.13. (*E*)-1-(3,4-Dichlorophenyl)-3-(4-methoxyphenyl)-2-methylprop-2-en-1-one(**3m**)

Synthesized from 3,4-dichlorophenone and 4-methoxybenzaldehyde, the reaction time is about 7 min, yellow solid, yield 51%, m.p. 70.0~71.1 °C; ^1^H NMR (400 MHz, Chloroform-d) δ 7.78 (d, *J* = 1.2 Hz,1H), 7.52 (dd, *J* = 8.6, 2.2 Hz, 1H), 7.40 (d, *J* = 8.6 Hz, 1H), 7.16–7.12 (m, 3H), 7.00–6.85 (m, 2H), 3.84 (s, 3H), 2.26 (s, 3H).^13^C NMR (101 MHz, Chloroform-d) δ 196.87, 160.33, 143.46, 138.70, 135.75, 134.27, 132.75, 131.81(2C), 131.23, 130.26, 128.50, 127.93, 114.11(2C), 55.38, 14.28. HRMS (ESI) *m*/*z* calcd for C_17_H_15_Cl_2_O_2_^+^ (M + H)^+^ 321.04436, found 321.04437.

#### 3.2.14. (*E*)-1-(3,4-Dichlorophenyl)-3-(4-(dimethylamino)phenyl)-2-methylprop-2-en-1-one(**3n**)

Synthesized from 3,4-dichlorophenone and 4-dimethylaminobenzaldehyde, the reaction time is about 3 min, yellow solid, yield 97%, m.p. 184.0~184.8 °C; ^1^H NMR (400 MHz, Chloroform-d) δ 8.15 (d, *J* = 2.3 Hz, 1H), 7.95 (dd, *J* = 8.3, 2.3 Hz, 1H), 7.73 –7.61 (m, 3H), 6.75 (d, *J* = 8.2 Hz, 1H), 6.65–6.55 (m, 2H), 3.85 (s, 6H), 2.92 (s, 3H).^13^C NMR (101 MHz, Chloroform-d) δ 201.00, 149.62, 137.72, 136.15, 133.57, 131.03, 130.95, 130.57, 129.63(2C), 127.60(2C), 124.38, 112.20(2C), 40.42(2C), 14.63. HRMS (ESI) *m*/*z* calcd for C_18_H_18_Cl_2_NO^+^ (M + H)^+^ 334.07600, found 334.07617.

#### 3.2.15. (*E*)-1-(3,4-Dichlorophenyl)-3-(4-(diethylamino)phenyl)-2-methylprop-2-en-1-one(**3o**)

Synthesized from 3,4-dichlorophenone and 4-diethylaminobenzaldehyde, the reaction time is about 5 min, yellow solid, yield 28%, m.p. 186.7~190.8 °C; ^1^H NMR (400 MHz, Chloroform-d) δ 7.80 (d, *J* = 2.2 Hz, 1H), 7.65 (dd, *J* = 8.4, 2.1 Hz, 1H), 7.54 (d, *J* = 8.5 Hz, 1H), 7.27 (q, *J* = 1.4 Hz, 1H), 7.17–7.06 (m, 2H), 7.00–6.87 (m, 2H), 3.93 (q, *J* = 7.0 Hz, 4H), 2.29 (s, 3H), 1.27 (t, *J* = 7.3 Hz, 6H).^13^C NMR (101 MHz, Chloroform-d) δ 196.82, 149.97, 148.83, 143.58, 138.65, 135.80, 134.54, 132.79, 131.21, 131.09, 128.48, 128.18, 123.66, 113.00, 111.01, 55.96(2C), 22.24, 14.35(2C). HRMS (ESI) *m*/*z* calcd for C_20_H_22_Cl_2_NO^+^ (M + H)^+^ 362.10730, found 362.24139.

#### 3.2.16. (*E*)-1-(2,4-Dichlorophenyl)-3-(4-(diethylamino)phenyl)-2-methylprop-2-en-1-one(**3p**)

Synthesized from 2,4-dichlorophenone and 4-diethylaminobenzaldehyde, the reaction time is about 5 min, yellow oil, yield 23%; ^1^H NMR (400 MHz, Chloroform-d) δ 8.14 (d, *J* = 8.9 Hz, 1H), 7.94 (dt, *J* = 8.4, 2.3 Hz, 2H), 7.63 (dd, *J* = 8.4, 2.3 Hz, 2H), 6.71 (d, *J* = 8.1 Hz, 1H), 6.52 (d, *J* = 8.2 Hz, 2H), 3.90–3.78 (m, 2H), 3.69–3.58 (m, 2H), 3.40–3.25 (m, 3H), 1.14 (td, *J* = 7.1, 2.2 Hz, 3H), 1.12–0.98 (m, 3H).^13^C NMR (101 MHz, Chloroform-d) δ 201.12, 149.22, 147.03, 137.66, 136.23, 133.54, 130.96, 130.92, 130.57, 129.80(2C), 127.59, 123.19, 111.39(2C), 44.17, 43.34, 14.72, 12.66(2C). HRMS (ESI) *m*/*z* calcd for C_20_H_22_Cl_2_NO^+^ (M + H)^+^ 362.10730, found 362.10718.

### 3.3. Biological Assays

#### 3.3.1. Cell lines and Cell Culture

HeLa and SiHa cell lines were obtained from the Procell Life Science Technology Co., Ltd. (Wuhan, China); H8 cell line was obtained from the Shanghai HonSun Bio-logical Technology Co., Ltd. (Shanghai, China); C33A and HeLa/DDP cell line was obtained from the Guangzhou Jenniobio Biotechnology Co., Ltd. (Guangzhou, China). HeLa, SiHa, H8, and HeLa/DDP cells were cultured in DMEM medium (HyClone). All of the mediums were supplemented with 10% FBS, 100 µg/mL penicillin, and 100 µg/mL streptomycin. All of the cells were cultivated in a 5% CO_2_ incubator at 37 °C.

#### 3.3.2. Anticervical Cancer Activity

All cell types, at a volume of 200 µL per well, were inoculated in 96-well plates at a density of 2.5 × 10^4^ cells / mL and cultured for 24 h in an incubator with 5% CO_2_ at 37 °C. The old culture medium was replaced with 200 µL of fresh medium per well containing positive compounds, at concentrations of 1, 6.25, 12.5, 25, 50, and 100 μM. After 48 h of culture, 20 µL per well of 5 mg·mL^−1^ MTT solution was added. After 4 h of culture, the old solution was discarded, and 150 µL per well of DMSO solution was added. Using an enzyme labeler (Thermo Fisher Scientific, Multiskan GO, Waltham, MA, USA), the plates were shaken for 10 min, OD values were determined at 490 nm, and the inhibition rate was calculated. Non-linear regression fitting of the cell growth rate was carried out using SPSS 23.0, and the IC_50_ of the compound was calculated. The inhibition rate was calculated using the formula: [(OD_empty_ − OD_test_)/(OD_empty_ − OD_negative group_)] × 100%, (OD_empty_ blank group absorbance, OD_test_ absorbance of administration group, OD_negative group_ without cell culture medium). Each group contained six compound holes, and the experimental results averaged three trials. Complete medium containing only cells was used as the blank control; a complete medium without cells as was used as the negative control; and cisplatin, paclitaxel, and adriamycin were used as the positive controls. The concentration gradient was the same as that used for compounds **3a**–**3p**.

#### 3.3.3. Pro-Apoptosis Assay

Cell fluid (2 mL per well) at a concentration of 1.5 × 10^5^ cells / mL was added to a 6-well plate and cultured in a cell incubator with 5% CO_2_ at 37 °C for 24 h. The old solution was discarded; 2 mL of the medium containing compound **3k** was added to each well at a concentration gradient of 2.5, 5, and 10 μM; and the experiment was repeated three times at each concentration. Complete medium containing only cells was used as a blank control group, and 20 μM of cisplatin injection and chalcone was used as a positive control. Cells were harvested following a 24 h incubation period, adhering to the guidelines provided with the BD kit (BD Biosciences Pharmingen, 556547, 10975 Torreyana Road, San Diego, CA, USA). Subsequently, the cells were subjected to staining in a light-restricted environment at ambient temperature for a duration of 15 min, followed by filtration and analysis via flow cytometry.

#### 3.3.4. Western Blotting Analysis

HeLa cells were cultured in 60 mm Petri dishes at a density of 1.0 × 10^6^ cells per dish and treated with 0.1% DMSO (vehicle), positive control chalcone (20 μM), and different concentrations of compound 3k (2.5, 5, and 10 μM), respectively. Following a 24 and 48 h incubation period, the cells were harvested via centrifugation and subsequently washed twice with PBS that had been chilled to 0 °C. Next, the cells were homogenized using RIPA lysis buffer (Solarbio, Beijing, China) and supplemented with 1% PMSF (MP Biomedicals, 0754-25G, CAS:329-98-6, Santa Ana, CA, USA) and 1% mixed phosphatase inhibitor (Solarbio, Beijing, China). The resulting lysates were then incubated on ice for 30 min, intermittently vortexed every 5 min, and subsequently centrifuged at 12,000 rpm for 20 min to collect the supernatants.

Subsequently, the protein concentrations were determined utilizing a BCA Protein Assay Kit (Solarbio, China). An equivalent quantity of the proteins (12 μg) was fractionated via 8–12% sodium dodecyl sulfate–polyacrylamide gel electrophoresis (SDS-PAGE) and transferred onto nitrocellulose membranes (Amersham Biosciences, Amersham, UK). Following this, the membranes were obstructed with 5% nonfat dried milk in TBS containing 1% Tween-20 for a duration of 2 h at ambient temperature and were subjected to overnight incubation with specific primary antibodies (CST, Boston, MA, USA) at 4 °C. Following three washes in TBST, the membranes were subjected to incubation with the suitable HRP-conjugated secondary antibodies at ambient temperature for a duration of 2 h. The blots were subsequently visualized using enhanced chemiluminescence (Biosharp, Shanghai, China) and detected utilizing a PE 0723 imager (ProteinSimple, Silicon Valley, CA, USA). Each experiment was conducted in triplicate at minimum and analyzed employing Image J software (V1.8.0.112). β-actin(ab198991) rabbit mAb, goat anti-rabbit IgG (H + L) HRP—#S0001 (Affinity). Bax (ab289364), Bcl-2 (ab218123), MDR1/ABCB1 (E1Y7B) rabbit mAb #13342 (CST).

#### 3.3.5. Cell Cycle Experiments

A 2 mL volume of cell fluid per well at a concentration of 1 × 10^6^ cells / mL was inoculated into a 6-well plate and cultured for 24 h. After removing the old solution, 2 mL of the cell culture medium containing **3k** was added to each well, and each well was exposed for 24 h to a concentration gradient of 2.5, 5, and 10 μM. The experiment was repeated three times for each concentration. Complete culture medium containing only cells was used as the blank control group, and 20 μM cisplatin and chalcone injection were used as the positive control group. After 24 h of treatment, the cells were collected in a 15 mL centrifuge tube and fixed with 75% ethanol at 4 °C for 24 h. A 400 µL volume of PI/RNase staining buffer (BD Biosciences Pharmingen, 550825, 10975 Torreyana Road, San Diego, CA, USA) was uniformly dispersed and subjected to staining for 15 min at room temperature without light shielding. The sample was subsequently filtered and analyzed using up-flow cytometry (BD, LSRFortessa, New York, NY, USA).

#### 3.3.6. Invasion and Migration Experiments

HeLa (5 × 10^4^ cells) was suspended in 200 μL of FBS-free medium. The top chamber contained the vehicle and various concentrations of compound **3k** (2.5, 5, and 10 μM), cisplatin (20 μM) and chalcone (20 μM). The lower chamber was filled with 600 μL of medium containing 10% FBS. After incubating at 37 °C for 24 h, the cells on the top side of the Transwell membrane were removed with a cotton tip. Cells were fixed and stained with 4% paraformaldehyde (LEAGENE BIOTECHNOLOGY, Cat:DF0135, Lot:0823A23, Zhongguancun, Haidian District, Beijing, China) and 0.1% crystal violet solutions (Leagene Biotechnology, Cat:DZ0055, Lot:0508A23, Zhongguancun, Haidian District, Beijing, China) for 30 min to fix cells trapped on the perforated side of the membrane.

The Transwell (12 mm pore, Corning Incorporated, New York, NY, USA) was precoated using 50 μL Matrigel for 5 h at 37 °C to achieve solidification. HeLa cells was collected and suspended in a serum-free medium containing varying concentrations (0, 2.5, 5, and 10 μM) of compound **3k**, along with 20 μM cisplatin and chalcone. These cell suspensions were then added to the upper wells of a permeabilization chamber at a density of 5 × 10^5^ cells/mL. In the lower wells, 600 μL of DMEM containing 10% FBS was added, which had been prepared using 50 μL of Matrigel (diluted 1:8 with serum-free medium from Corning/BD Biosciences). After 24 h of incubation at 37 °C, the invasion cells were fixed using 4% paraformaldehyde and stained using 0.1% crystal violet for 30 min. Then, the chambers were washed using PBS and left to dry. Images were photographed using an inverted fluorescence microscope (Leica Instruments Co., Ltd., Microsystems CMS GmbH, Weizler, Hesse, Germany) and counted using Image J software (V1.8.0.112) for three independent fields randomly.

#### 3.3.7. Anti-Cisplatin-Resistant Cervical Cancer Activity

To mitigate the detrimental consequences arising from the fixation of resistance reversal agents, concentrations that yielded a tumor cell proliferation rate reduction of less than 10% were selected. Consequently, the inhibitory efficacy of compound **3k** against HeLa/DDP cells was assessed in conjunction with cisplatin at concentrations of 0.25, 0.5, and 1 μM. The experimental procedure employed was akin to that elucidated in Section 3.3.2. The impact of verapamil (6 μM) and **3k** (0.25, 0.5, and 1 μM) on P-gp expression was ascertained via Western blot experiments, employing the same methodology delineated in Section 3.3.4.

Rhodamine 123 (RL0073, Ex/Em:507/529 nm, CAS:62669-70-9, Changsha Hanchen Biotechnology Co., Ltd. (Changsha, China) a frequently employed cationic green fluorescent dye, possesses the ability to traverse cell membranes, leading to its accumulation within the mitochondria of viable cells, ultimately emitting a yellow-green fluorescence. This dye is extensively utilized as a fluorescent probe for the identification of mitochondrial membrane potential, while maintaining its non-toxic nature toward cells. To achieve a final concentration of 5 mM, Rhodamine 123 is dissolved in DMSO. The resulting solution is then dispensed and stored at a temperature of −20 °C, ensuring protection from light. The experimental procedure involved the addition of 1 drop of PBS to each well of a 12-well plate, followed by the placement of cell crawlers and removal of excess PBS. HeLa/DDP cells were then plated at a concentration of 1.5 × 10^5^ cells/mL, with 1 mL per well, and cultured for a duration of 24 h. A blank control group was established alongside a positive group treated with verapamil (6 μM) and an intervention group treated with **3k** (0.25, 0.5, and 1 μM) for a duration of 48 h. The drug-containing culture medium was subsequently washed with PBS three times, with each wash lasting 5 min. The Rh123 master mix was diluted with culture medium to create a 20 μM Rh123 buffer, which was subsequently incubated at a temperature of 37 °C for a duration of 1 h. Following this incubation period, the Rh123 buffer was removed, and the cells were subjected to three consecutive washes with PBS, each lasting for 10 min. Subsequently, a fixation process was conducted using 4% paraformaldehyde at a volume of 1 mL per well, with a duration of 20 min. This was followed by three additional washes with PBS, each lasting for 10 min. Finally, a drop of blocking agent was applied to the slide. A small amount of sealer was applied onto the slide, followed by the removal of the crawler. Subsequently, the side containing cells was affixed onto the liquid and made ready for measurement. The cells were then examined using a fluorescence microscope (Leica, Microsystems CMS GmbH, GER) equipped with a fluorescein filter, and the fluorescence intensity of each distinct group was assessed.

#### 3.3.8. Pro-Apoptosis Assay (HeLa/DDP)

The pro-apoptotic experiments of compound **3k**, cisplatin, and chalcone on HeLa/DDP were performed in the same way as in Section 3.3.3. Western Blotting Analysis in the same way as in Section 3.3.4.

#### 3.3.9. Cell Cycle Experiments (HeLa/DDP)

The experimental methods for the effect of compound **3k**, cisplatin, and chalcone on the HeLa/DDP cell cycle were the same as in Section 3.3.5.

#### 3.3.10. Invasion and Migration Experiments (HeLa/DDP)

The experimental methods for the effects of compound **3k**, cisplatin, and chalcone on HeLa/DDP cell invasion and migration were the same as in Section 3.3.6.

#### 3.3.11. Molecular Docking

In this research, the lead compounds chalcone and **3k** were subjected to molecular docking analyses with Tubulin and P-gp proteins. The protein crystal structures utilized in this study were obtained from the RCSB Protein Data Bank (PDB) database, accessible at http://www.rcsb.org/. The specific protein identification numbers assigned were Tubulin (1SA0) [41] and P-gp (7O9W) [42], respectively. To prepare the protein crystals for analysis, a “.pdb” file format was generated by eliminating water molecules, metals, and ligands from the original file using PymoL1.7.6 software. The file was saved in the format of a “.pdb” file, the protein underwent hydrogenation using AutodockTools1.5.6 software, and the Gasteiger charges were automatically computed and subsequently saved as a PDBQT file for the purpose of backup.

After using ChemDraw 18.0 to draw the small molecules, Chem3D 18.0 was utilized to convert them into a 3D format. The “MM-2” function within the “Calculation” feature was then employed to select “Minimize energy” in order to optimize the energy. The optimized structure was subsequently saved as a “.mol2” file. Autodock software was utilized to convert the file into PDBQT format. The Autodock software was then used to open the PDBQT files of both the receptor structure and the ligand structure. Under the “GridBox” option, the center sites of the docked proteins (Tubulin: x = 53, y = 55, z = −5; P-gp: x = 135, y = 134, z = 156) were set.

The docking program was executed using the default algorithm and parameters of Autodock Vina. The resulting conformations were saved as a “.pdb” file for subsequent analysis as prediction outcomes. The 2D composite conformation of ligand–protein interactions was saved using Discovery Studio 2021 Client software, while the 3D composite conformation was saved using Pymol software. The Pymol software was employed to modify the coloration of the protein chains and small molecules, to investigate the interaction forces between the small molecules and proteins, and to compute the interaction forces between small molecules and proteins using the “Wizard” tool. The Pymol software was utilized to modify the color of protein chains and small molecules, investigate the interaction force between small molecules and proteins, determine the interaction distance between small molecules and proteins using the “Wizard” tool, and generate a high-resolution image by means of the “ray” command.

#### 3.3.12. Immunofluorescence Assay

HeLa or HeLa/DDP cells were seeded at a density of 5 × 10^4^ cells/mL in 12-well plates with cell crawls, with 1 mL of cell suspension added to each well, and incubated for 24 h. Experimental groups were established, including a blank control group (0 μM), positive drug groups treated with paclitaxel (2.0 μM) and colchicine (2.0 μM), and intervention groups treated with **3k** at concentrations of 1, 2, and 4 μM, all for a duration of 24 h. The cells were then fixed by adding 400 μL of 4% paraformaldehyde to each well and incubating for 30 min. 0.1% Triton was added at a volume of 400 μL per well and allowed to incubate for 10 min. Following this, goat serum (10%) was added at 400 μL per well and incubated for 40 min. Primary antibodies (α-tubulin, β-tubulin) were then incubated overnight at a temperature of 4 °C. Subsequently, secondary antibodies (α-mouse, β-rabbit) were incubated for 1.5 h. DAPI staining was performed at 400 μL per well for 5 min, followed by a wash with PBS. Cell climbing tablets were removed and inverted onto slides with sealer. Photographs were captured using a fluorescent inverted microscope.

Polymerized and free microtubule proteins were extracted using the methods described in references [43], respectively. Following drug intervention, the cells were subjected to two washes with preheated microtubule protein buffer MTSB at 37 °C. Subsequently, 70 μL of MTSB was added, and the cells were lysed for 10 min at 37 °C in a constant temperature chamber shielded from light. The cells were then scraped with a spatula and centrifuged at 14,000 r/min for 10 min at room temperature. The resulting supernatant, which represented the free state microtubule protein suspension, was subsequently removed. The remaining precipitate was treated with RIPA lysis solution, followed by lysis on ice for a duration of 30 min. Subsequently, centrifugation was performed at a temperature of 4 °C and a speed of 12,000 revolutions per minute for a duration of 20 min. The resulting supernatant was collected as a suspension of polymerized microtubule protein. The Western blotting procedure was the same as in Section 3.3.4. α-Tubulin (DM1A) Mouse mAb#3873 (CST), β-Tubulin (D2N5G) Rabbit mAb #15115 (CST), CoraLite488-conjugated Goat Anti-Mouse lgG(H+L) (Proteintech); CoraLite488-conjugated Goat Anti-Rabbit lgG(H+L) (Proteintech); β-actin(ab198991) rabbit mAb, Goat Anti-Mouse IgG H&L (HRP) (ab6789); Goat anti-Rabbit IgG (H+L) HRP—#S0001 (Affinity).

#### 3.3.13. Statistical Analysis

Data were processed using SPSS 23.0. The measurement data are expressed as mean ± SD and analyzed using a one-way multi-sample ANOVA; the difference was statistically significant (*p* < 0.05, *p* < 0.01 and *p* < 0.001), unless a non-parametric test was used.

## 4. Conclusions

Compound **3k** demonstrated notable efficacy in inhibiting the proliferation of human cervical cancer cells, namely HeLa, SiHa, C33A and cisplatin-resistant HeLa/DDP cells, while exhibiting lower toxicity toward human cervical epithelial cells H8. Furthermore, the compound **3k** demonstrated a dose-dependent pro-apoptotic impact on both HeLa and HeLa/DDP cells, impeding their progression in the G2/M phase and substantially suppressing the invasive and migratory capabilities of cervical cancer cells. Subsequent investigations demonstrated that the co-administration of **3k** and cisplatin yielded a notable augmentation in the antiproliferative efficacy of cisplatin on HeLa/DDP cells. Furthermore, this combination had no discernible impact on the expression of P-gp proteins while concurrently promoting the accumulation of rhodamine 123 in HeLa/DDP cells. Consequently, this accumulation led to an elevation in fluorescence intensity, potentially attributable to the inhibition of P-gp’s transporter function by **3k**. In addition, molecular docking analysis showed that **3k** has some binding affinity for the active pockets of tubulin and P-gp. Subsequent investigations demonstrated that the compound **3k** exhibited the ability to diminish the fluorescence intensity of α and β microtubule proteins in both HeLa and HeLa/DDP cell lines. Furthermore, **3k** was found to reduce the quantities of polymerized α and β microtubulin while concurrently augmenting the expression of dissociated α and β microtubulin in the aforementioned cell lines, thereby indicating a more pronounced anti-microtubulin polymerization effect.

Hence, the potential anti-cervical activity of compound **3k** could be attributed to its ability to inhibit microtubulin and P-gp targets, consequently impeding the efflux function of P-gp and interfering with the mitotic process of tumor cells. As a result, it exhibits anti-cervical and anti-cisplatin-resistant cervical cancer effects. This investigation furnishes an empirical foundation for the development of innovative α-methyl chalcone compounds with anti-cervical cancer and cisplatin-resistant cervical cancer properties.

## Figures and Tables

**Table 1:** In vitro antitumor activities of the compounds against HeLa, SiHa, C33A, and H8 Cells.

Compound	IC50 48 h (μM) $\bar{x}$ ± SD			
	HeLa	SiHa	C33A	H8
**3a**	>100	23.47 ± 4.55 ***	69.92 ± 7.18	>100
**3b**	43.78 ± 3.68 **	28.41 ± 2.37 ***	56.98 ± 6.27	57.72 ± 4.70 ##
**3c**	>100	41.84 ± 5.10 **	33.85 ± 4.99	>100
**3d**	97.57 ± 7.61	>100	63.73 ± 2.59	>100
**3e**	>100	>100	>100	>100
**3f**	>100	>100	23.72 ± 4.07 **	>100
**3g**	56.05 ± 4.58 *	>100	44.79 ± 2.85	>100
**3h**	>100	>100	54.36 ± 11.52	>100
**3i**	>100	>100	30.67 ± 8.84	>100
**3j**	>100	>100	28.46 ± 3.78 *	>100
**3k**	9.05 ± 0.53 ***#	14.54 ± 0.98 ***##	12.11 ± 2.44 ***	>100
**3l**	>100	22.49 ± 1.36 ***	17.29 ± 2.24 ***	>100
**3m**	29.75 ± 0.98 ***	67.71 ± 5.60	35.12 ± 4.75	>100
**3n**	>100	>100	23.43 ± 3.42 **	>100
**3o**	>100	>100	34.97 ± 4.93	>100
**3p**	>100	>100	41.35 ± 5.42	>100
**Chalcone**	67.03 ± 4.56	62.03 ± 1.84	37.53 ± 1.50	78.45 ± 7.78
**Cisplatin**	13.03 ± 1.82	20.70 ± 1.85	6.05 ± 1.15	25.23 ± 1.29

Note: All the above results were expressed as the mean ± SD of three independent experiments: * *p* < 0.05, ** *p* < 0.01, *** *p* < 0.001 compared with the chalcone group. # *p* < 0.05, ## *p* < 0.01 compared with the cisplatin group.

**Table 2:** In vitro antitumor activities of the compounds against HeLa/DDP Cells.

Target Compound	$\mathrm{IC}50\;48\;h\;(\mu M)\;\bar{x}$ ± SD		
	HeLa	HeLa/DDP	RI
Cisplatin	13.03 ± 1.82	102.91 ± 3.60	7.90
Paclitaxel	22.98 ± 1.33	130.10 ± 5.84	5.66
Doxorubicin	10.43 ± 1.09	38.34 ± 4.57	3.68
Chalcone	67.03 ± 4.56	87.97 ± 7.27	1.31
**3k**	9.05 ± 0.53 #	9.72 ± 0.73 ###	1.07
Cisplatin + verapamil (6 μM)	14.59 ± 1.87	39.13 ± 6.05 ###	2.68
Cisplatin + 3k (0.25 μM)	14.54 ± 1.55	98.02 ± 10.83	6.74
Cisplatin + 3k (0.5 μM)	14.23 ± 0.45	68.84 ± 5.10 ###	4.84
Cisplatin + 3k (1 μM)	11.68 ± 2.57	24.54 ± 1.86 ###	2.10

Note: All the above results were expressed as the mean ± SD of three independent experiments: # *p* < 0.05, ### *p* < 0.001 compared with the cisplatin group.

**Table 3:** Molecular docking results of chalcone and candidate compound **3k** with tubulin and P-gp.

Ligand	Name of Protein	PDB ID	The Lowest Binding Energy (kcal/mol)	
			Compd	Hydrogen Bonds
Chalcone	Tubulin	1SA0	−6.4	-
	P-gp	7O9W	−8.5	1
Compound **3k**	Tubulin	1SA0	−7.1	1
	P-gp	7O9W	−7.4	-
CN2	Tubulin	1SA0	−8.8	10
UIC2-Fab	P-gp	7O9W	−10.8	2

## Data Availability

Data are contained within the article and Supplementary Materials.

## Supplementary Materials

The following supporting information can be downloaded at: https://www.mdpi.com/article/10.3390/molecules28237697/s1, ^1^H-NMR, ^13^C-NMR, and HRMS.
