# Supplementary material for: Design and Synthesis of Novel α-Methylchalcone Derivatives, Anti-Cervical Cancer Activity, and Reversal of Drug Resistance in HeLa/DDP Cells
Source: Molecules. 2023 Nov 21;28(23):7697. doi: 10.3390/molecules28237697 (PMC10707934; doi:10.3390/molecules28237697)
Supplement: Supplementary file 1 [file molecules-28-07697-s001.zip › molecules-2683281-supplementary.pdf]

Supplementary materials(LC-MS、^{1}H NMR and ^{13}C NMR)

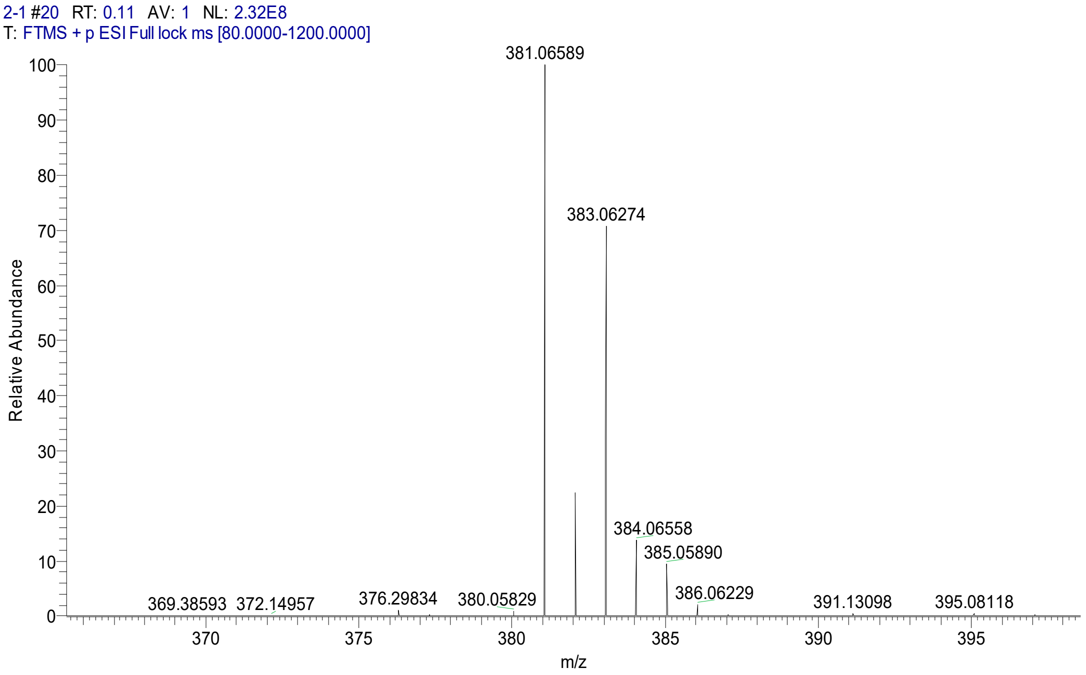

Figure. 1-1 Mass spectral data of compound 3a

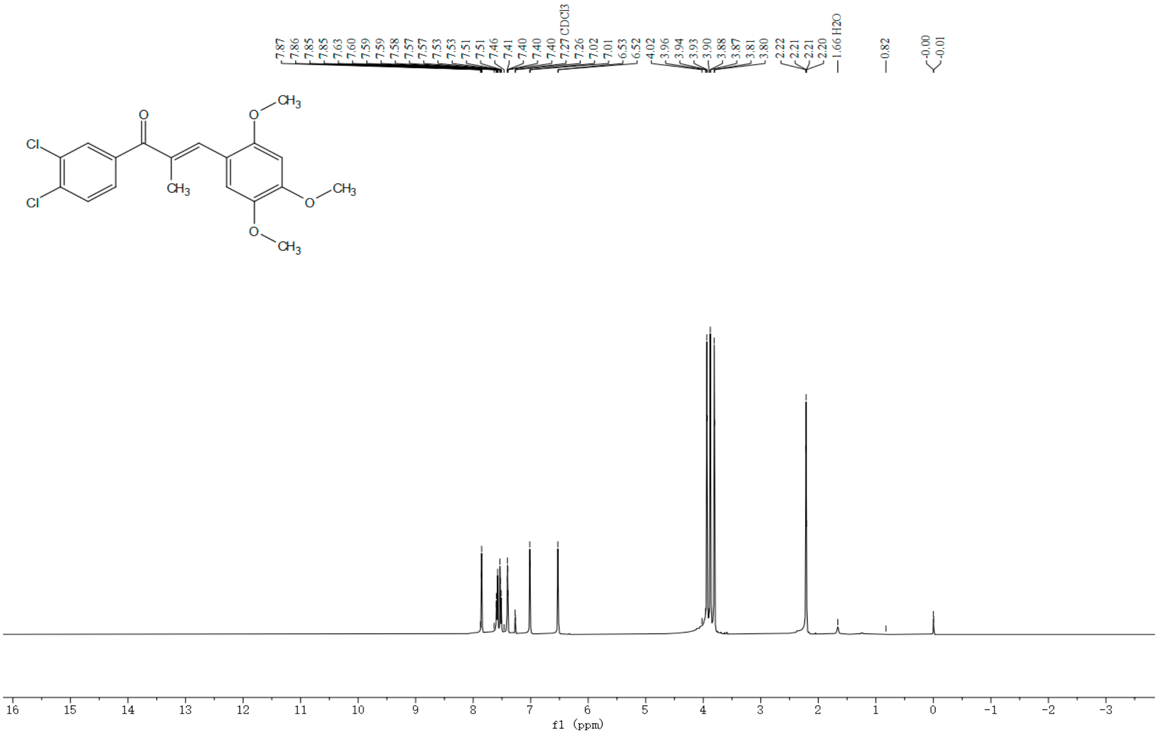

Figure. 1-2 ^{1}H NMR of compound 3a

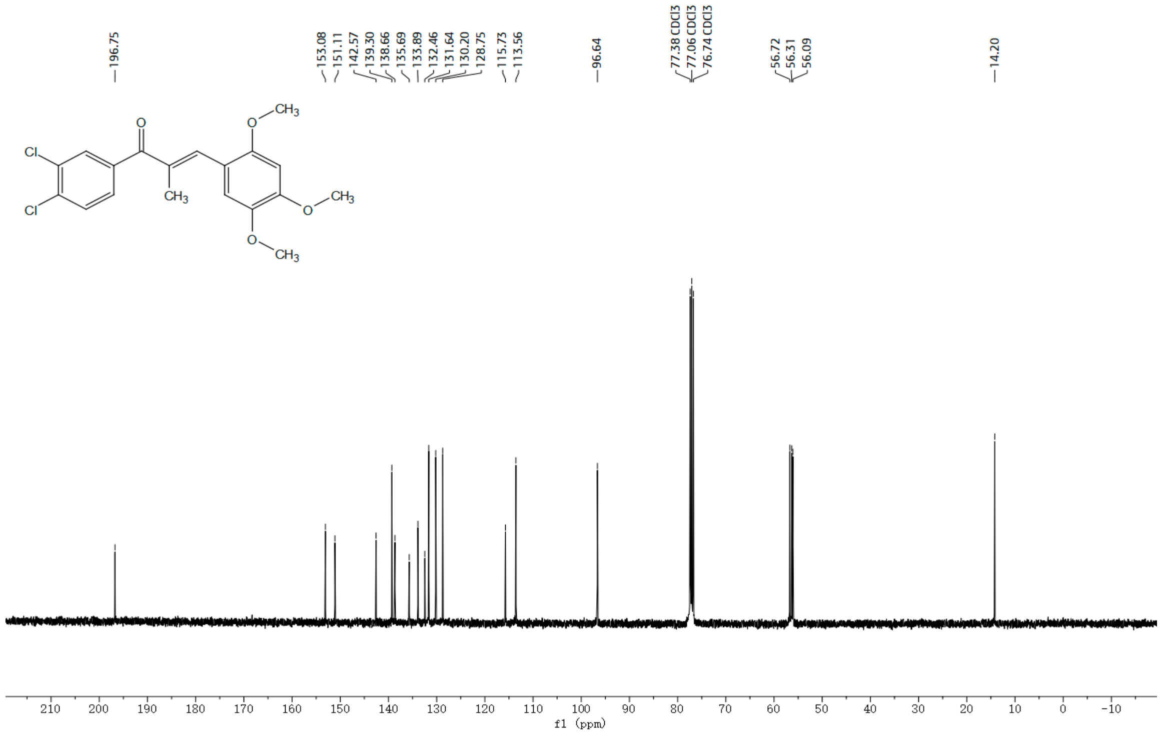

Figure. 1-3 ^{13}C NMR of compound 3a

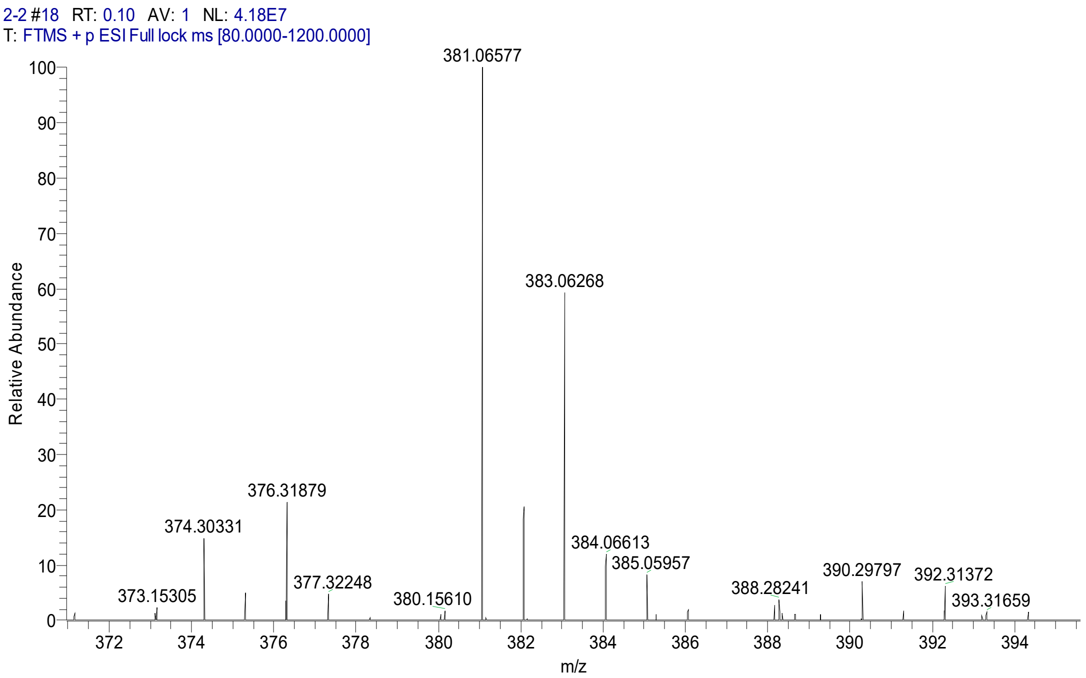

Figure. 2-1 Mass spectral data of compound 3b

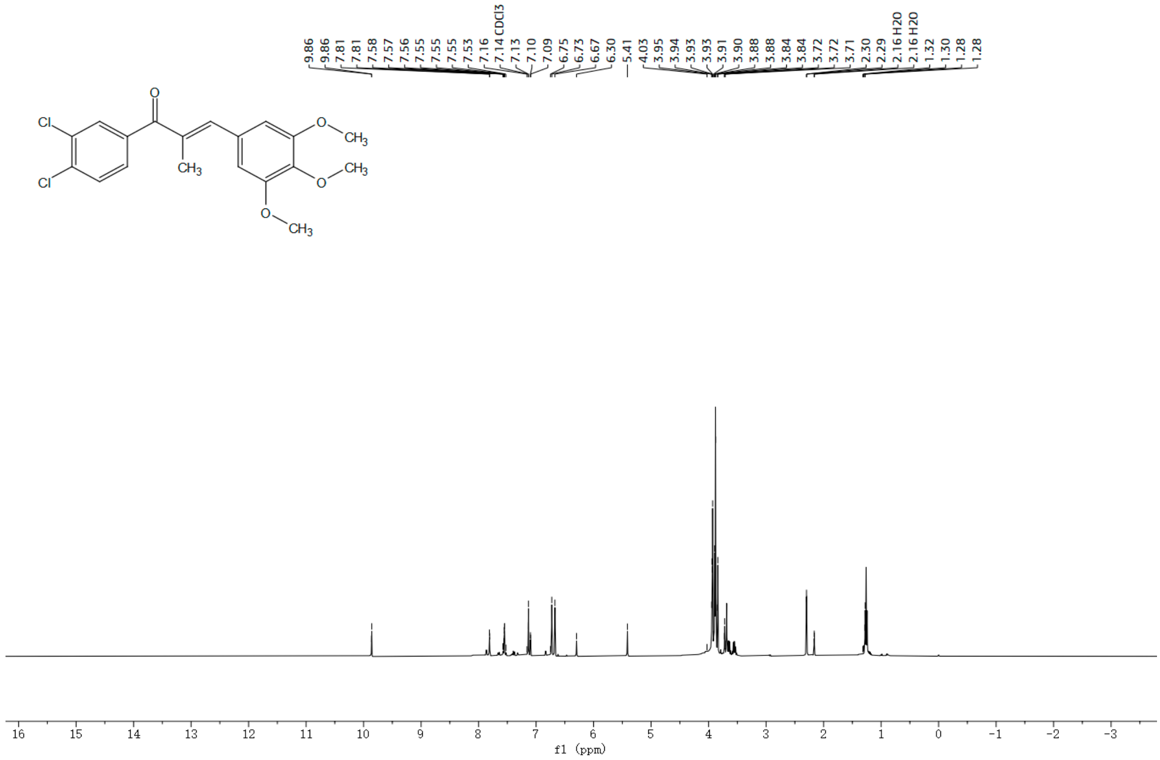

Figure. 2-2 ^{1}H NMR of compound 3b

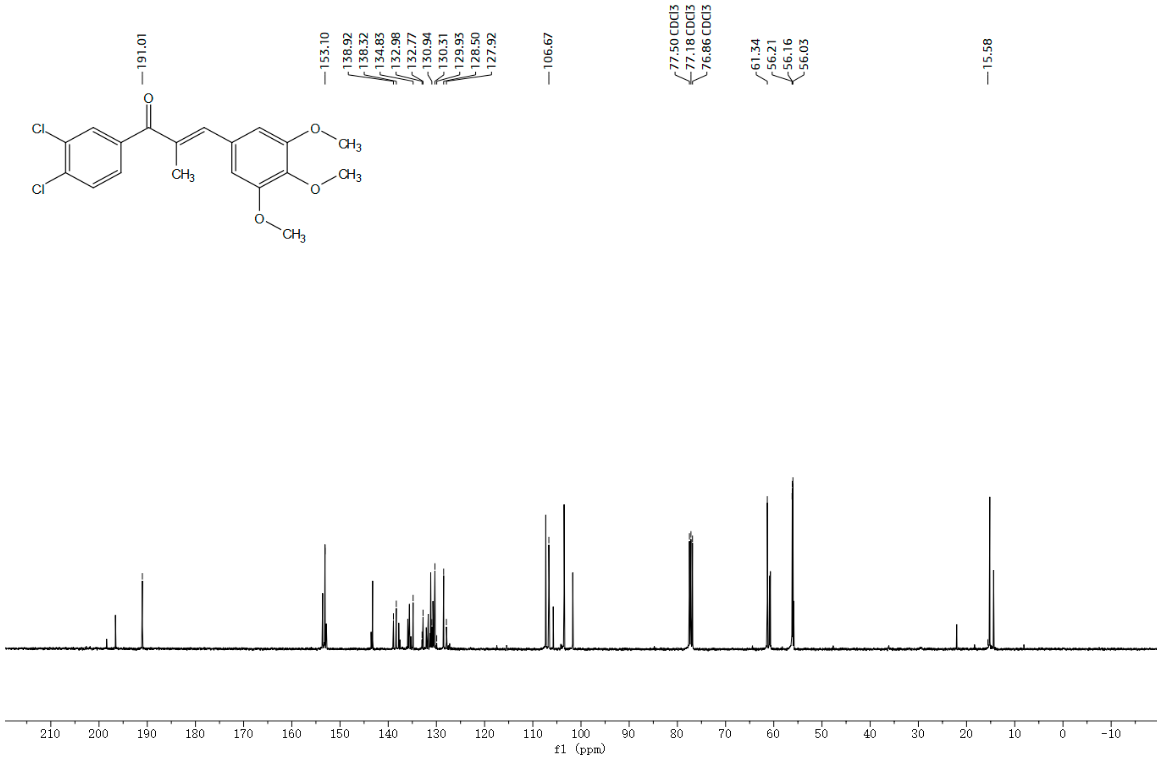

Figure. 2-3 ^{13}C NMR compound 3b

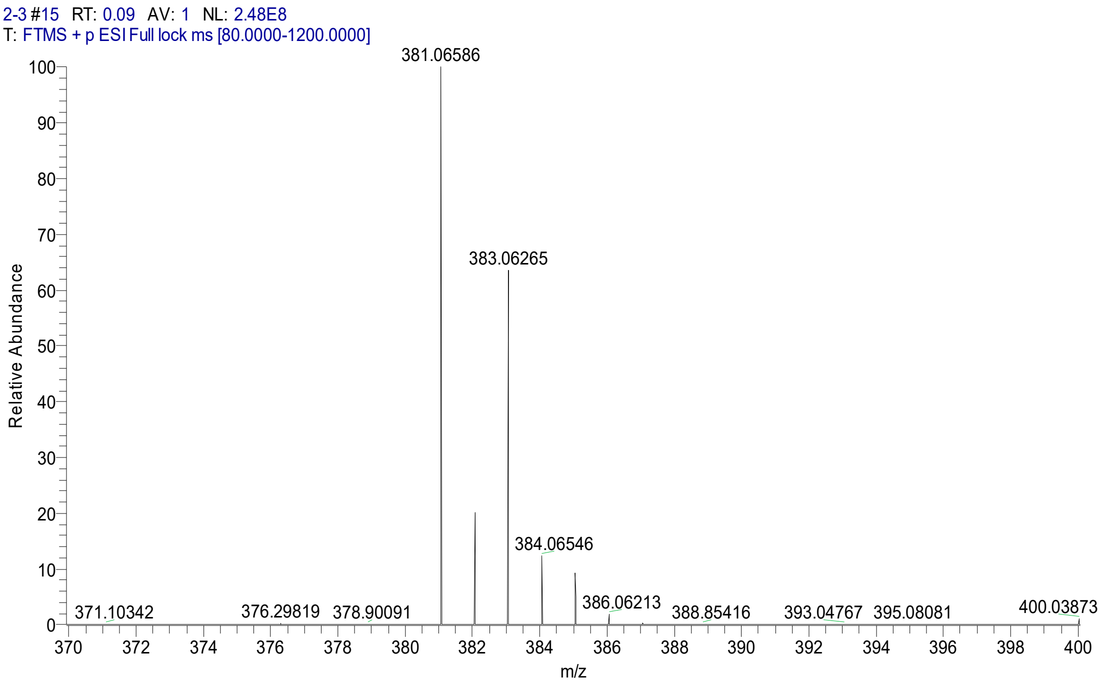

Figure. 3-1 Mass spectral data of compound 3c

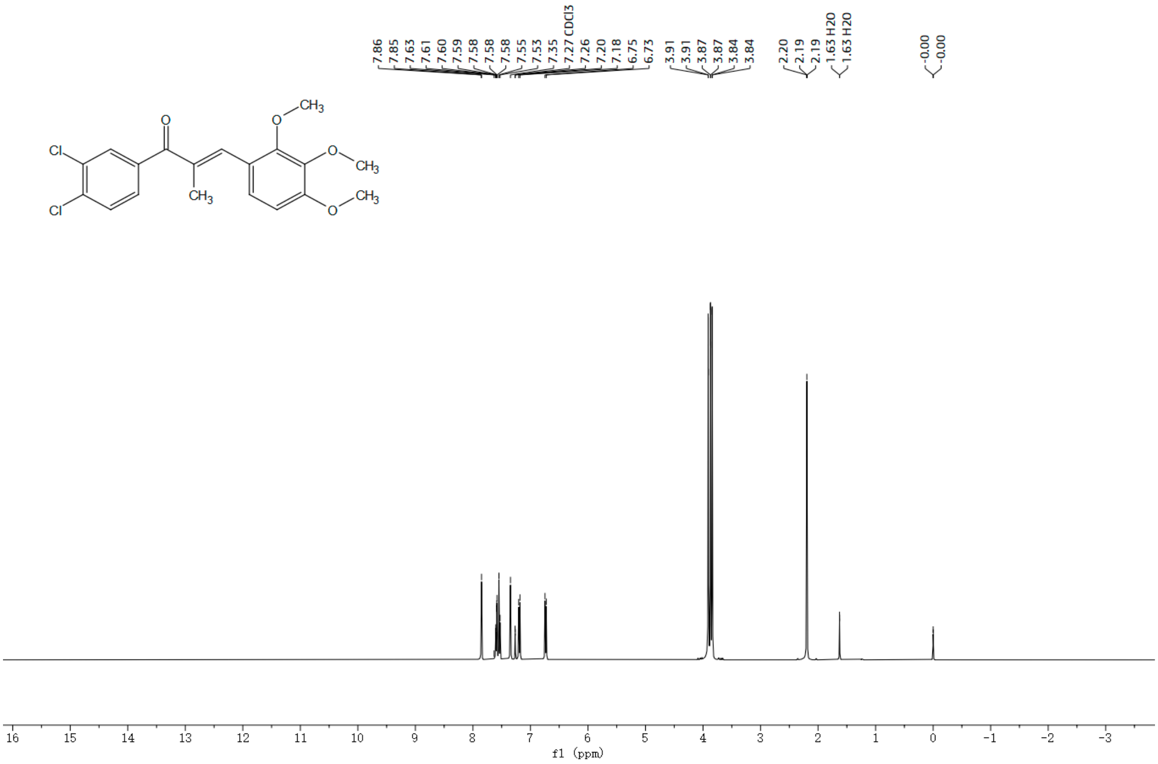

Figure. 3-2 ^{1}H NMR of compound 3c

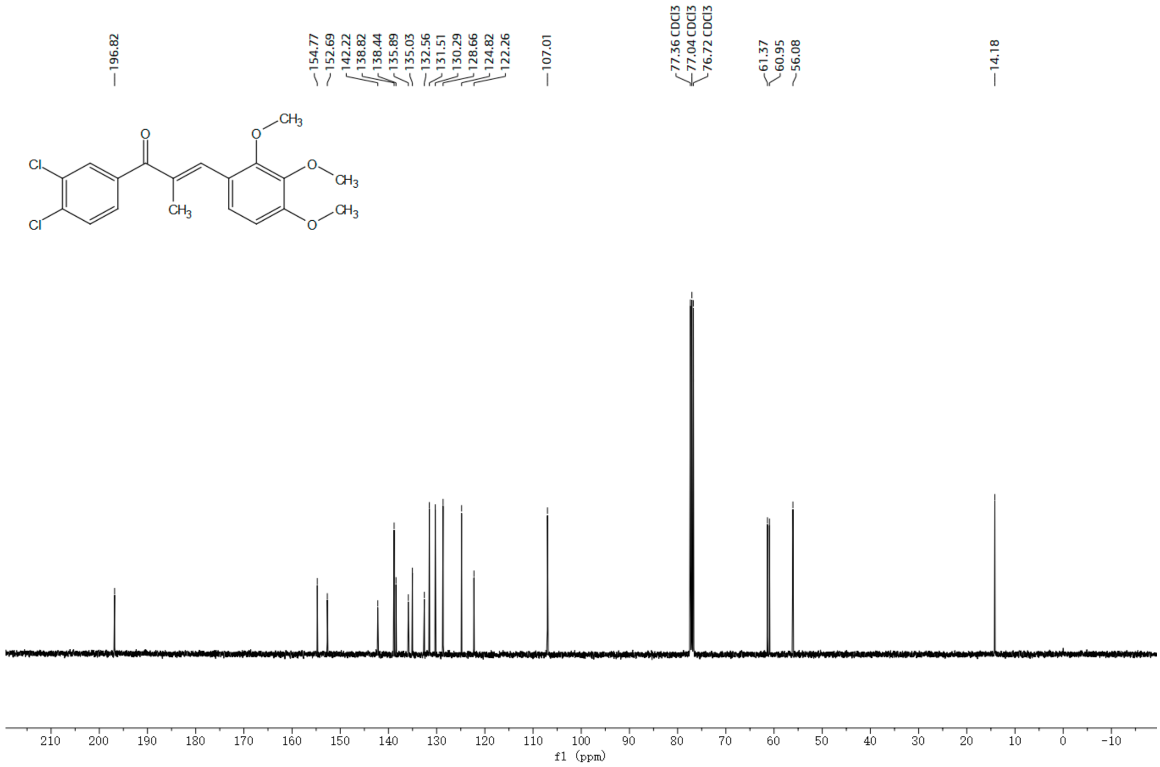

Figure. 3-3 ^{13}C NMR compound 3c

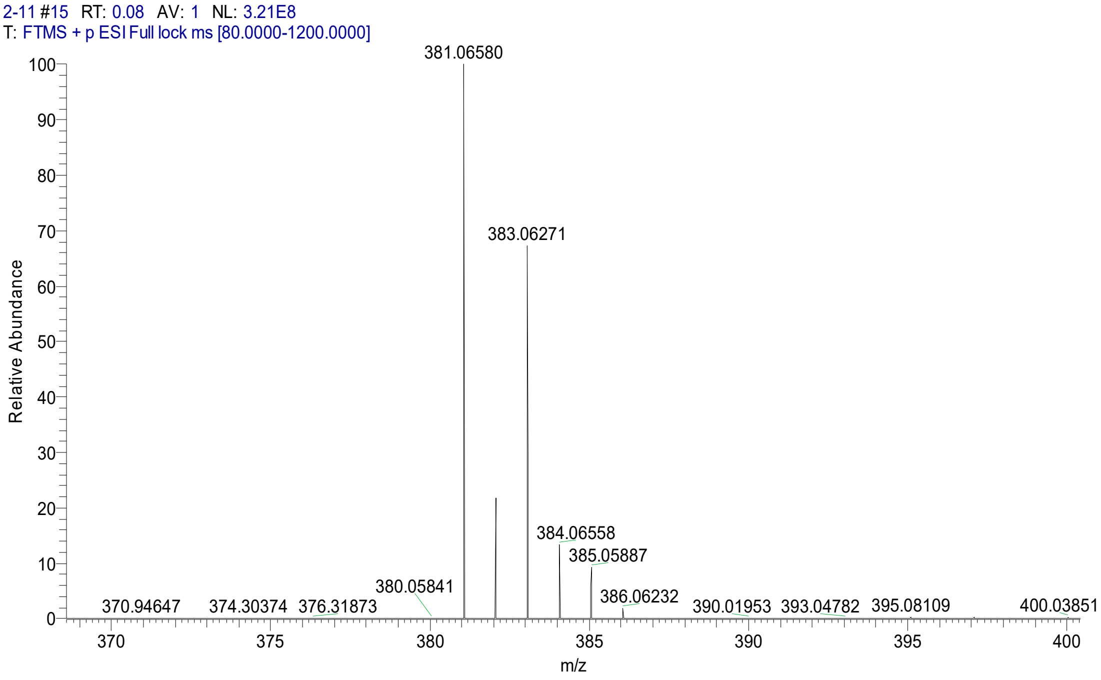

Figure. 4-1 Mass spectral data of compound 3d

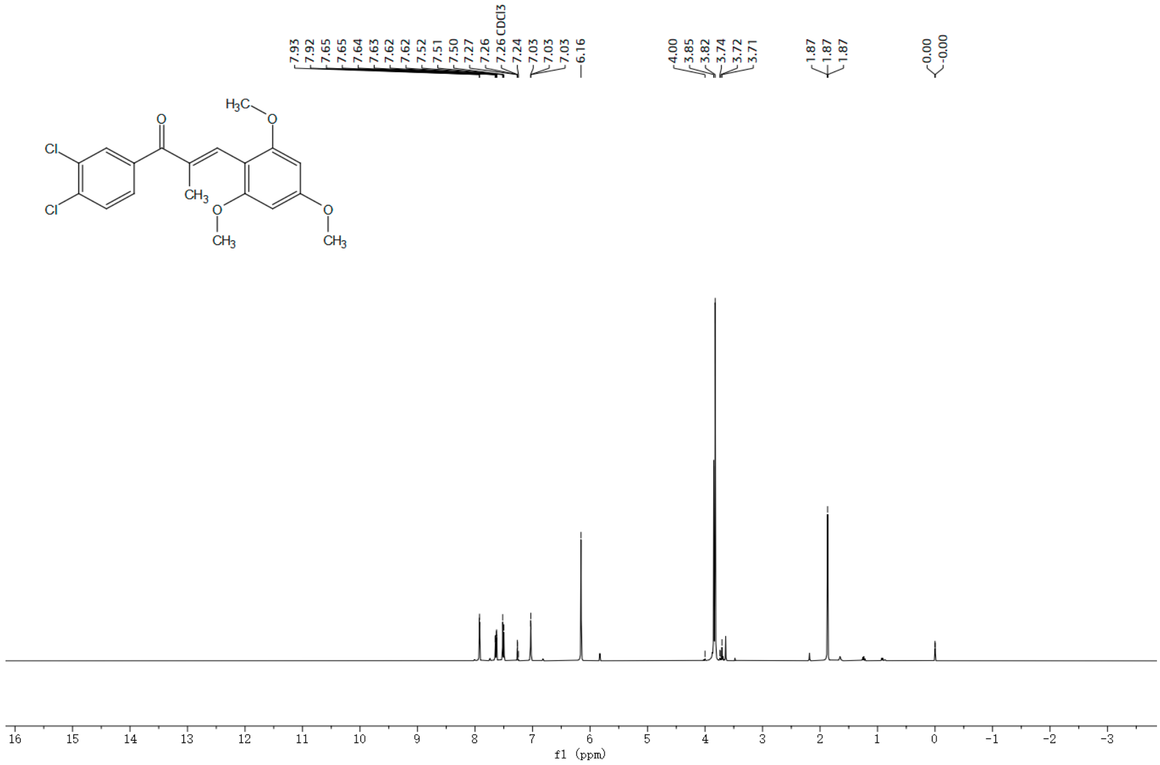

Figure. 4-2 ^{1}H NMR of compound 3d

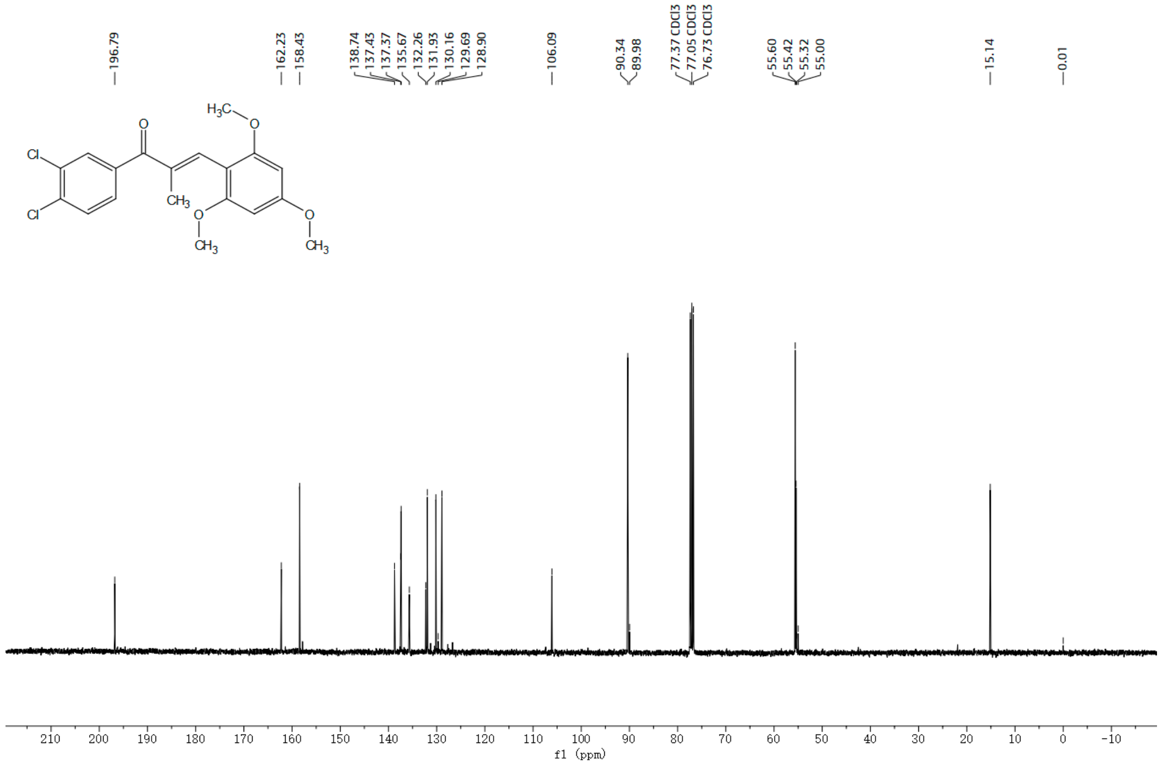

Figure. 4-3 ^{13}C NMR compound 3d

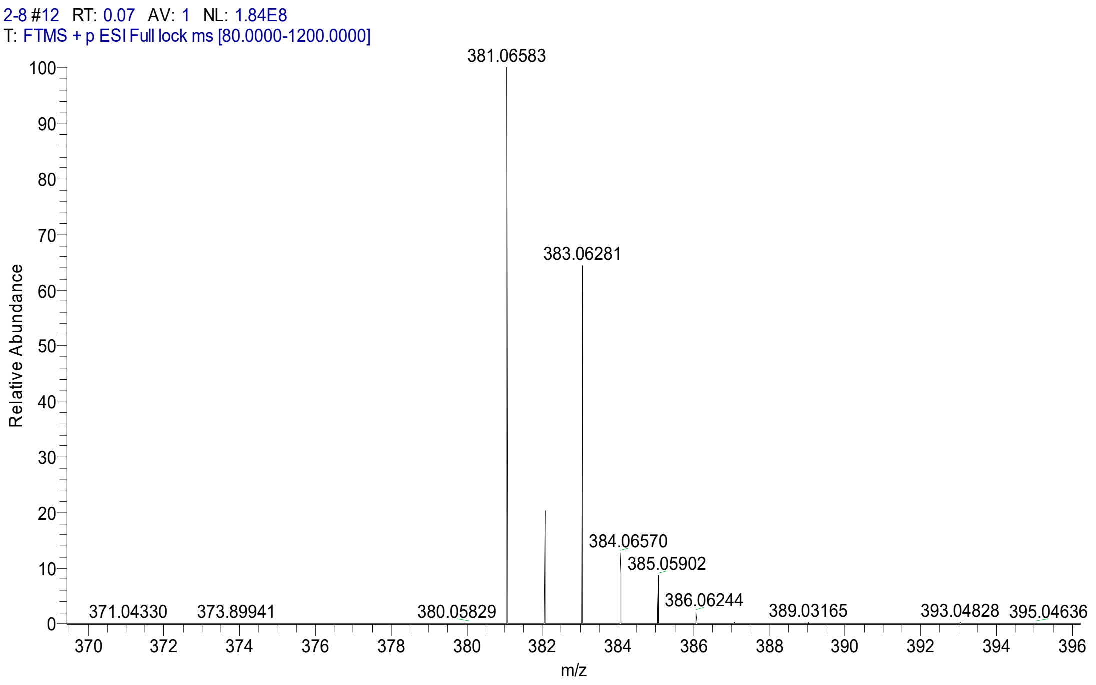

Figure. 5-1 Mass spectral data of compound 3e

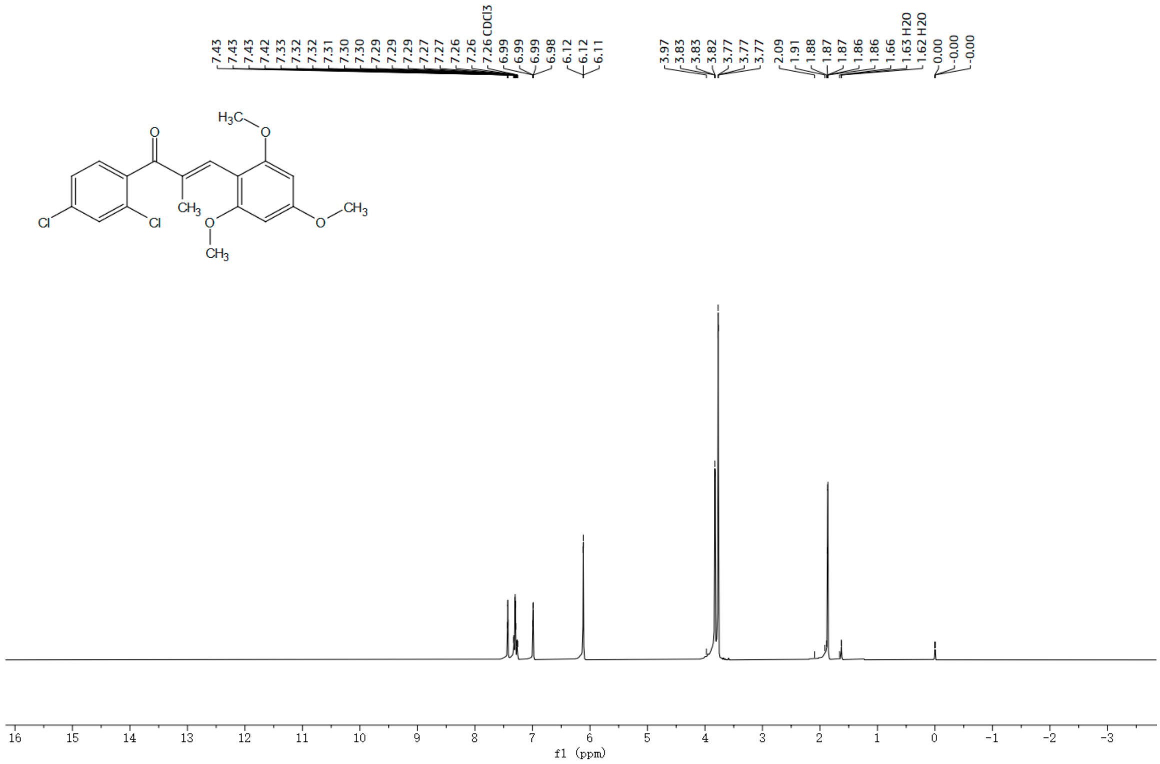

Figure. 5-2 ^{1}H NMR of compound 3e

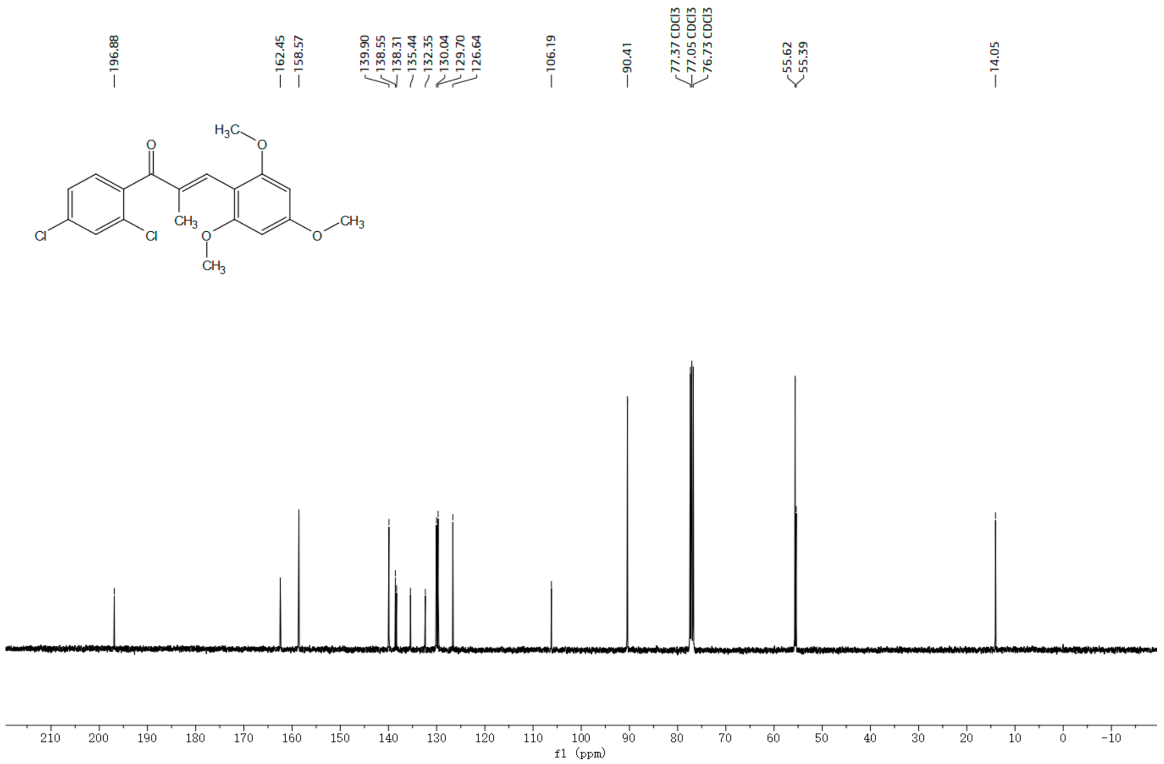

Figure. 5-3 ^{13}C NMR compound 3e

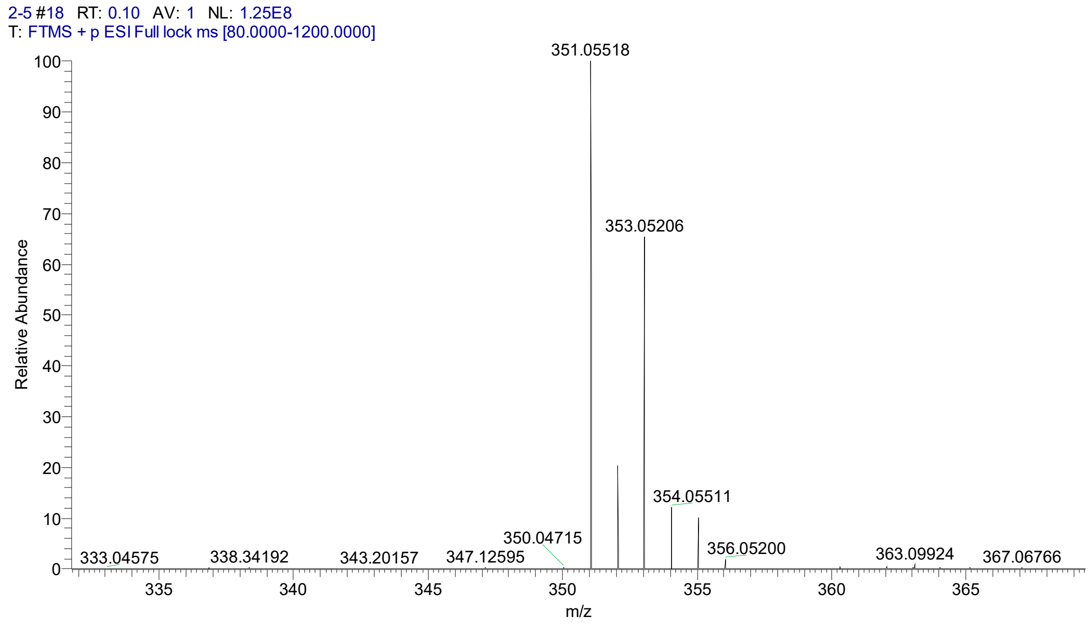

Figure. 6-1 Mass spectral data of compound 3f

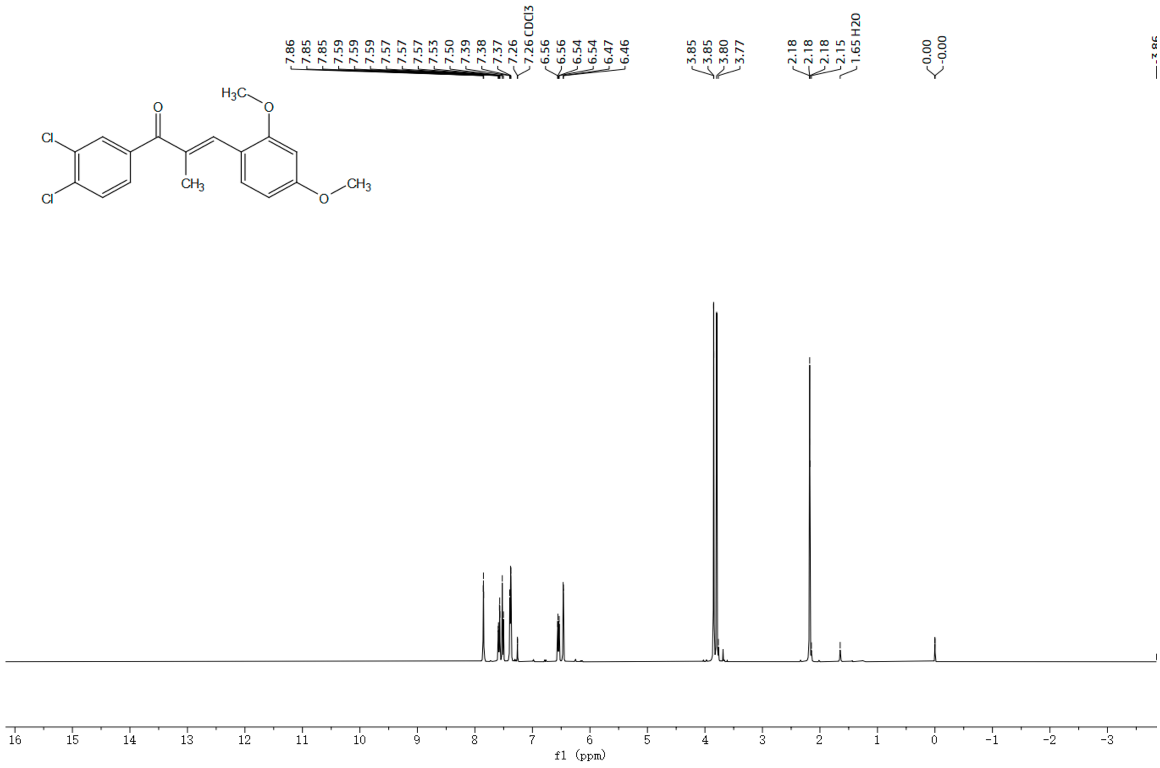

Figure. 6-2 ^{1}H NMR of compound 3f

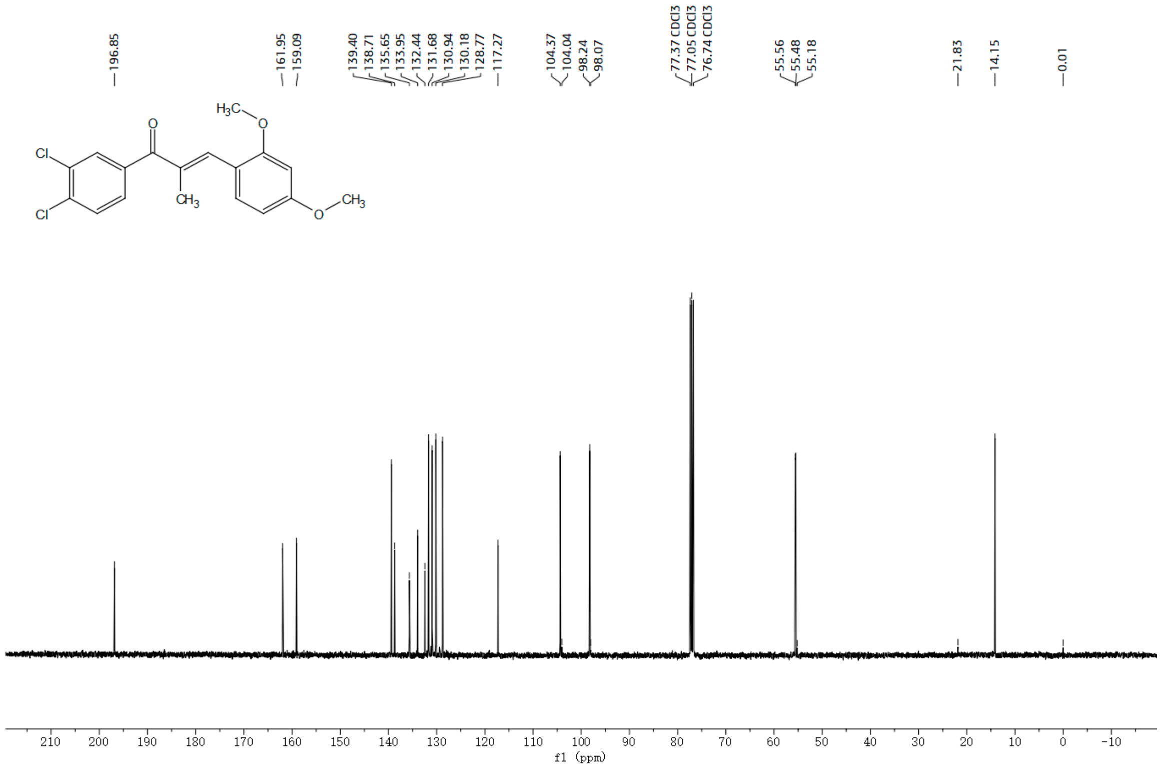

Figure. 6-3 ^{13}C NMR compound 3f

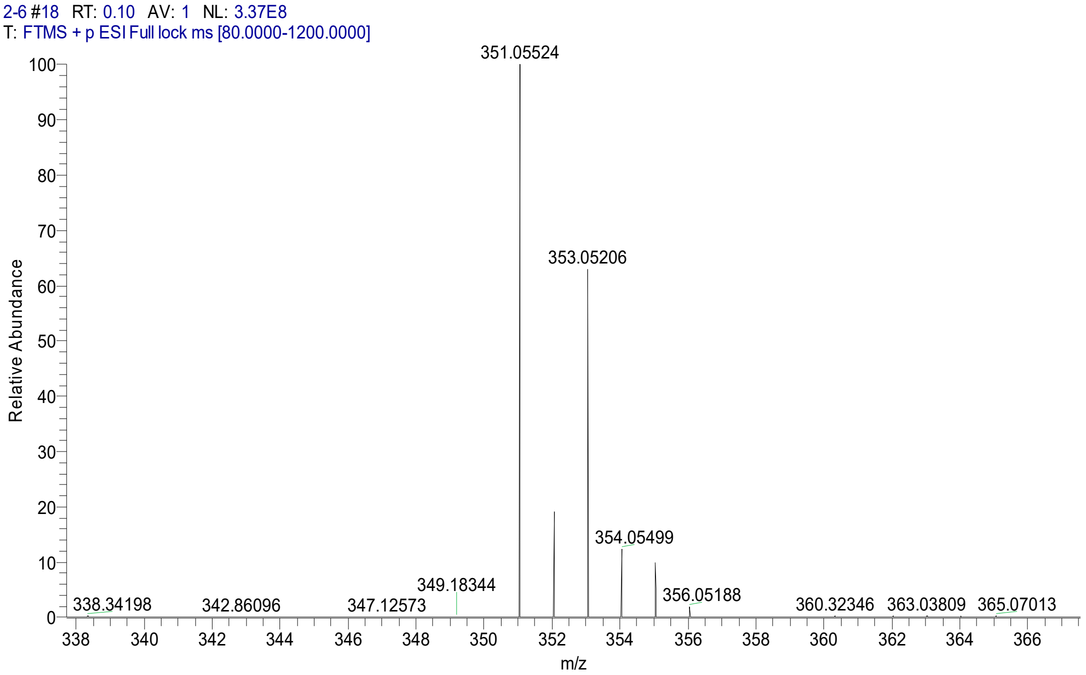

Figure. 7-1 Mass spectral data of compound 3g

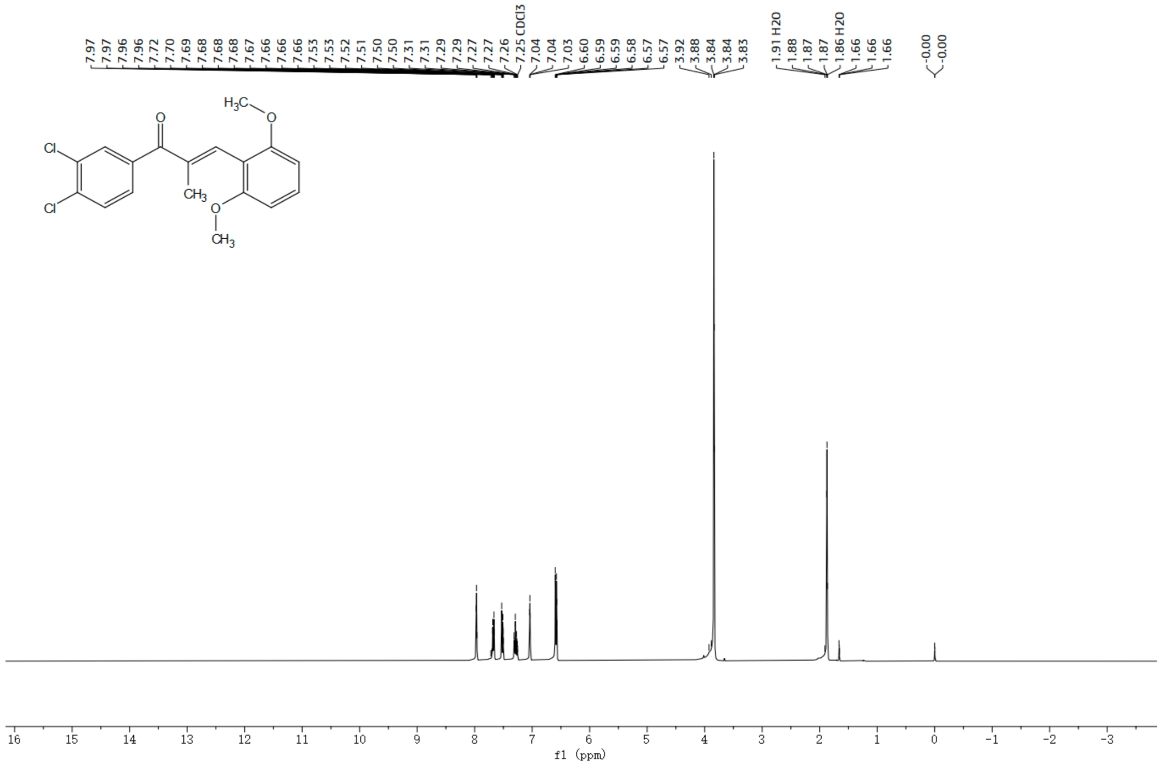

Figure. 7-2 ^{1}H NMR of compound 3g

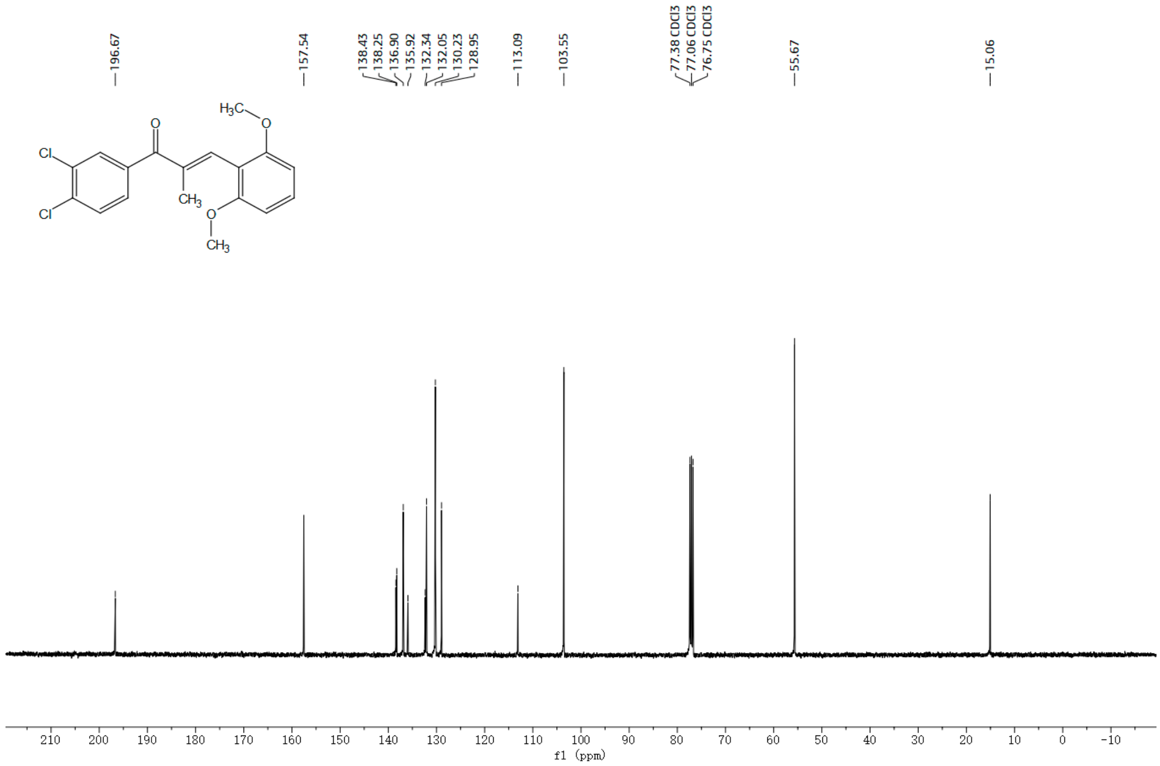

Figure. 7-3 ^{13}C NMR compound 3g

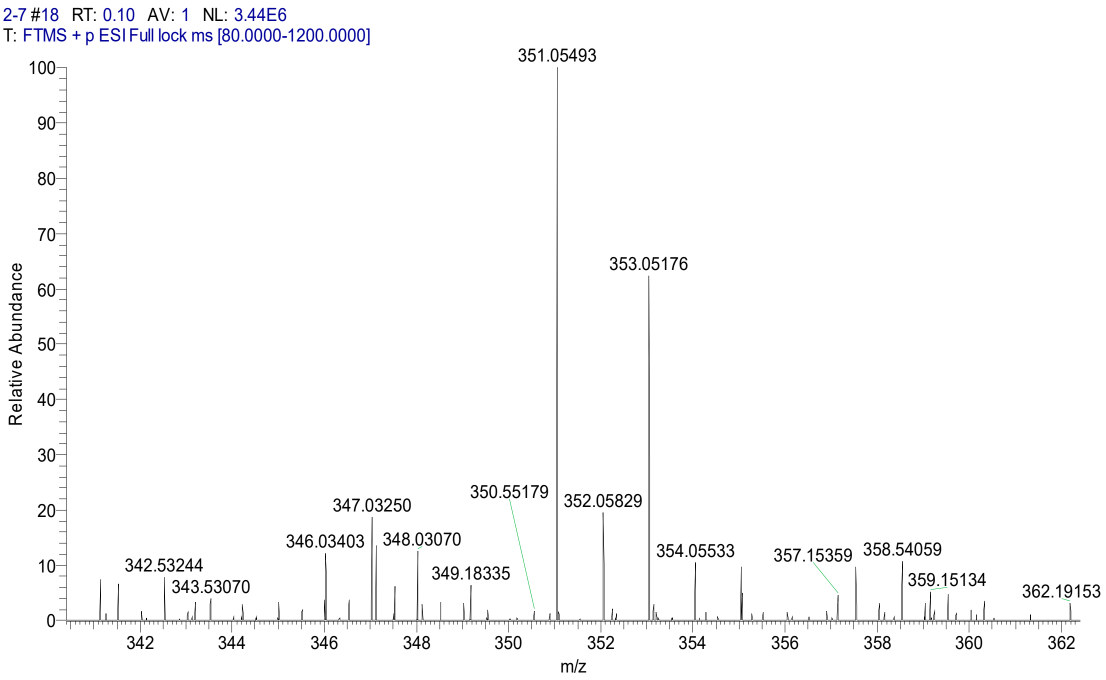

Figure. 8-1 Mass spectral data of compound 3h

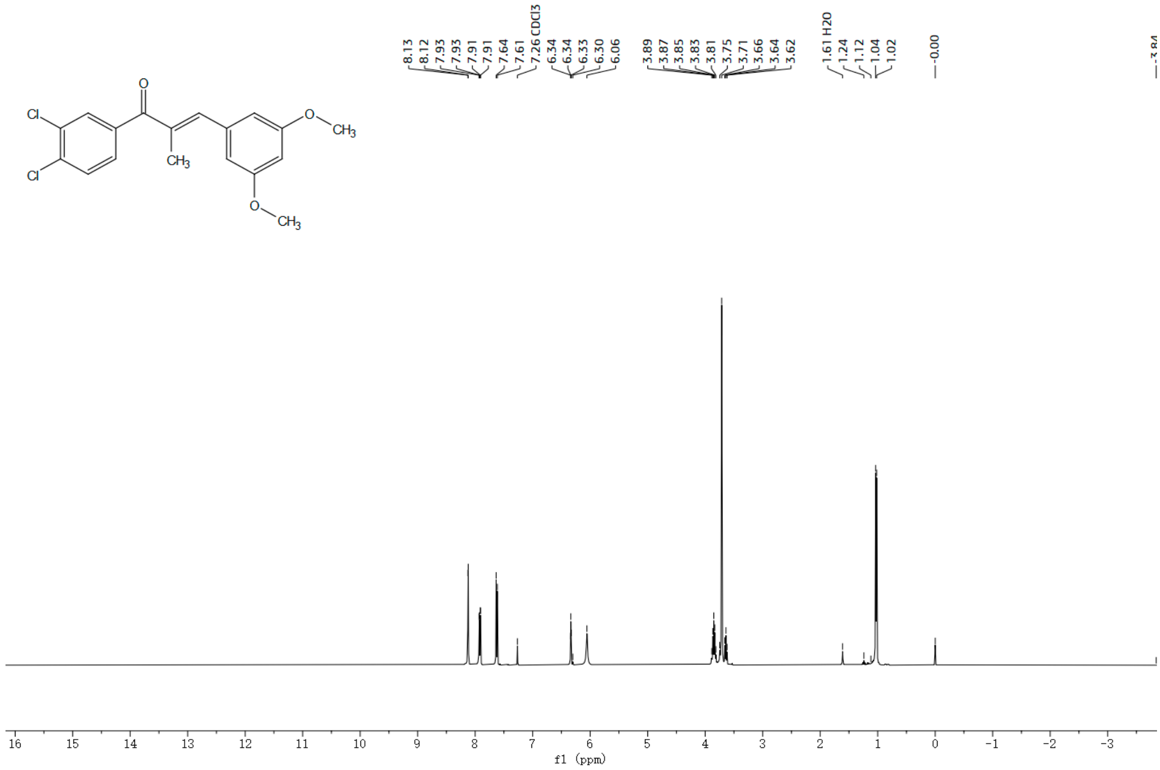

Figure. 8-2 ^{1}H NMR of compound 3h

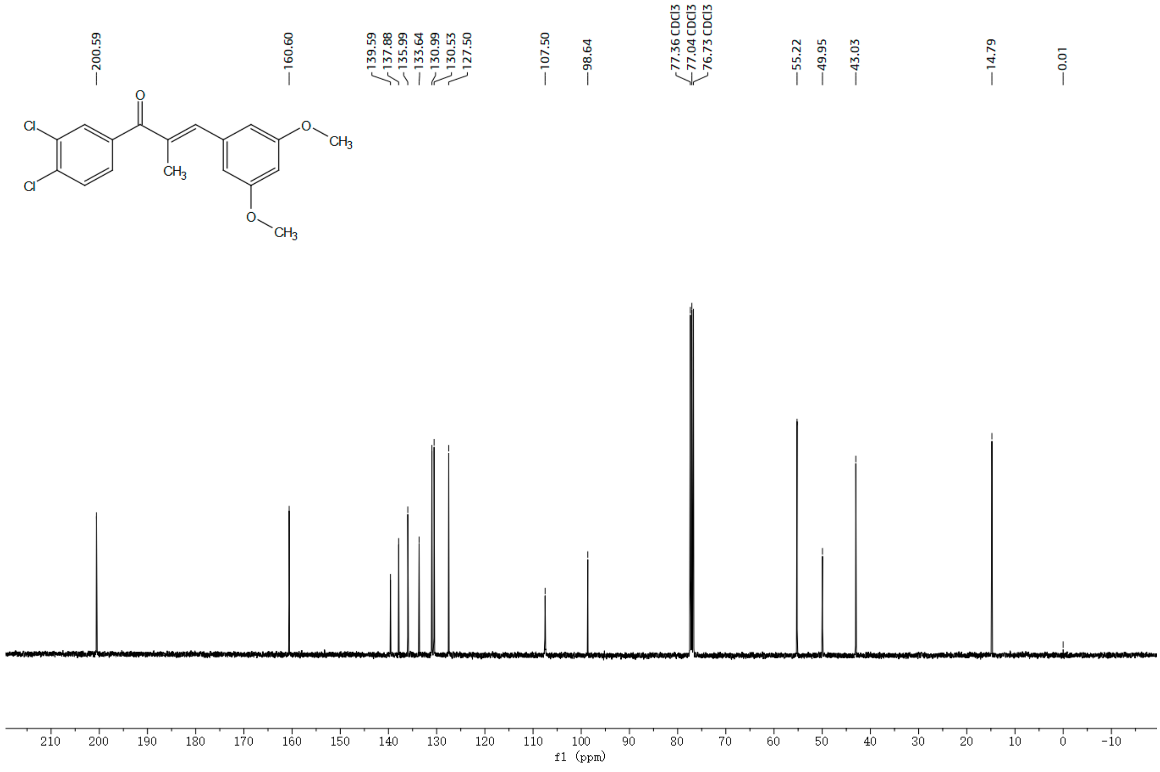

Figure. 8-3 ^{13}C NMR compound 3h

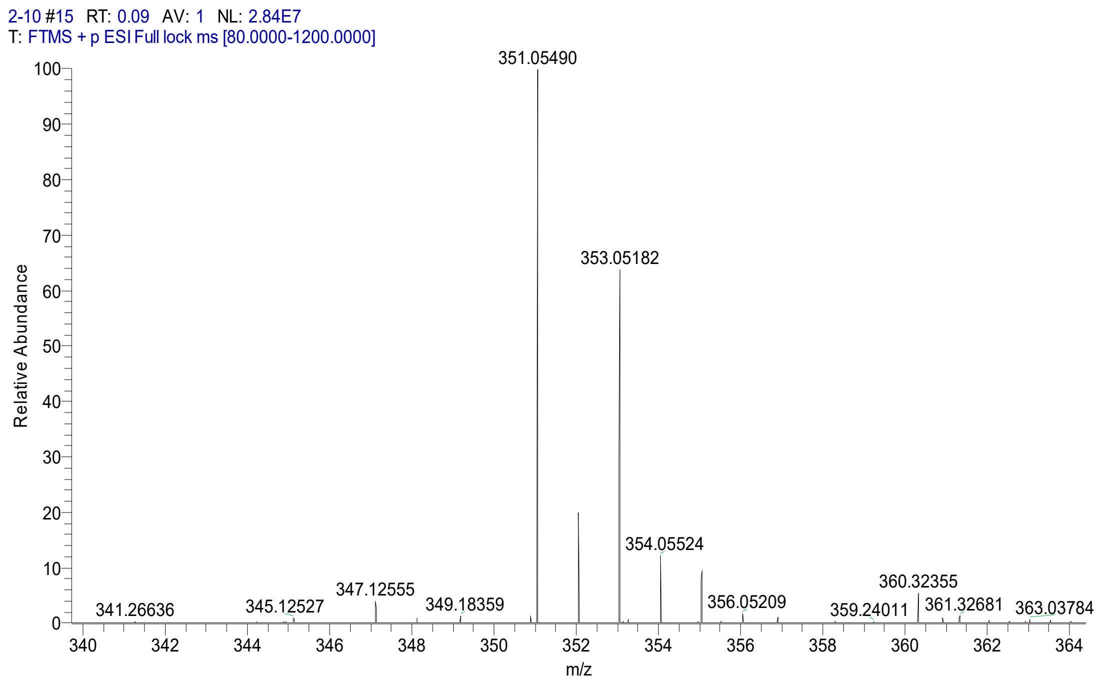

Figure. 9-1 Mass spectral data of compound 3i

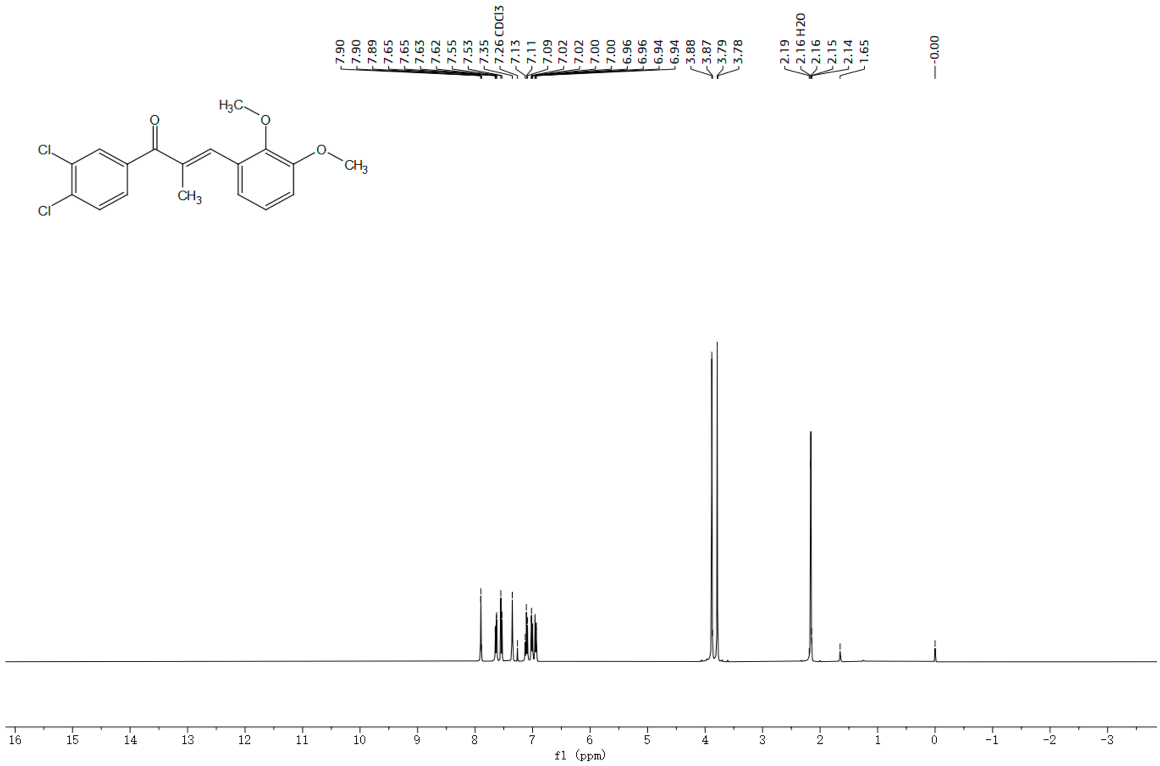

Figure. 9-2 ^{1}H NMR of compound 3i

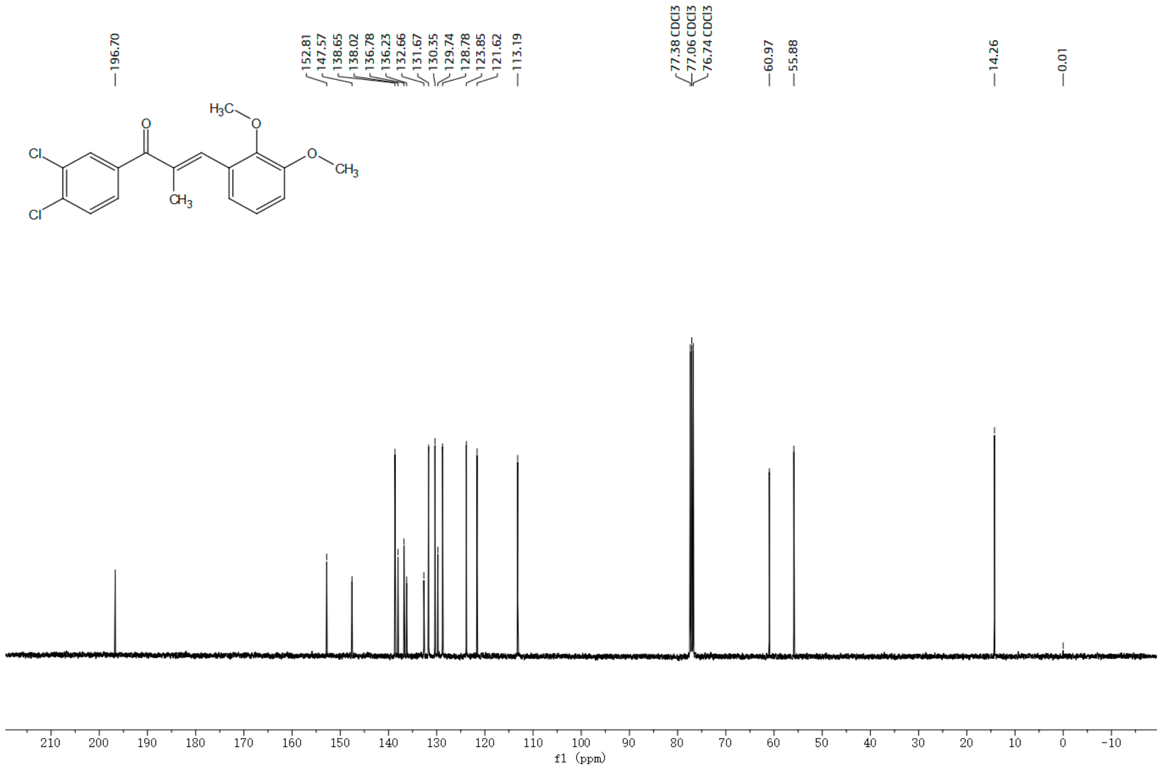

Figure. 9-3 ^{13}C NMR compound 3i

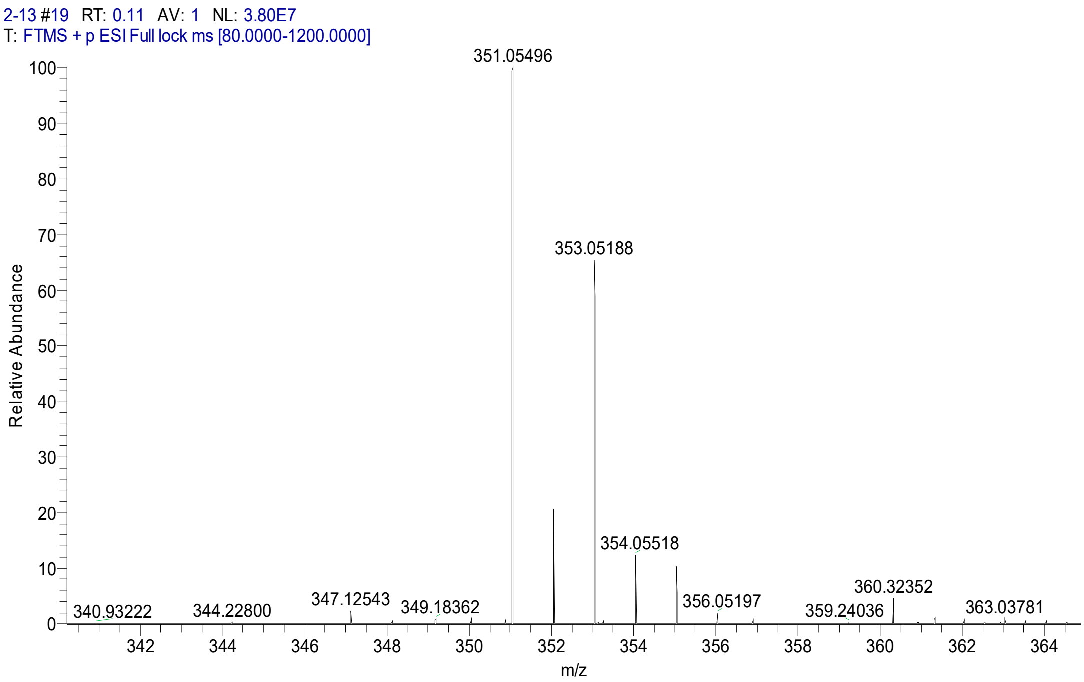

Figure. 10-1 Mass spectral data of compound 3j

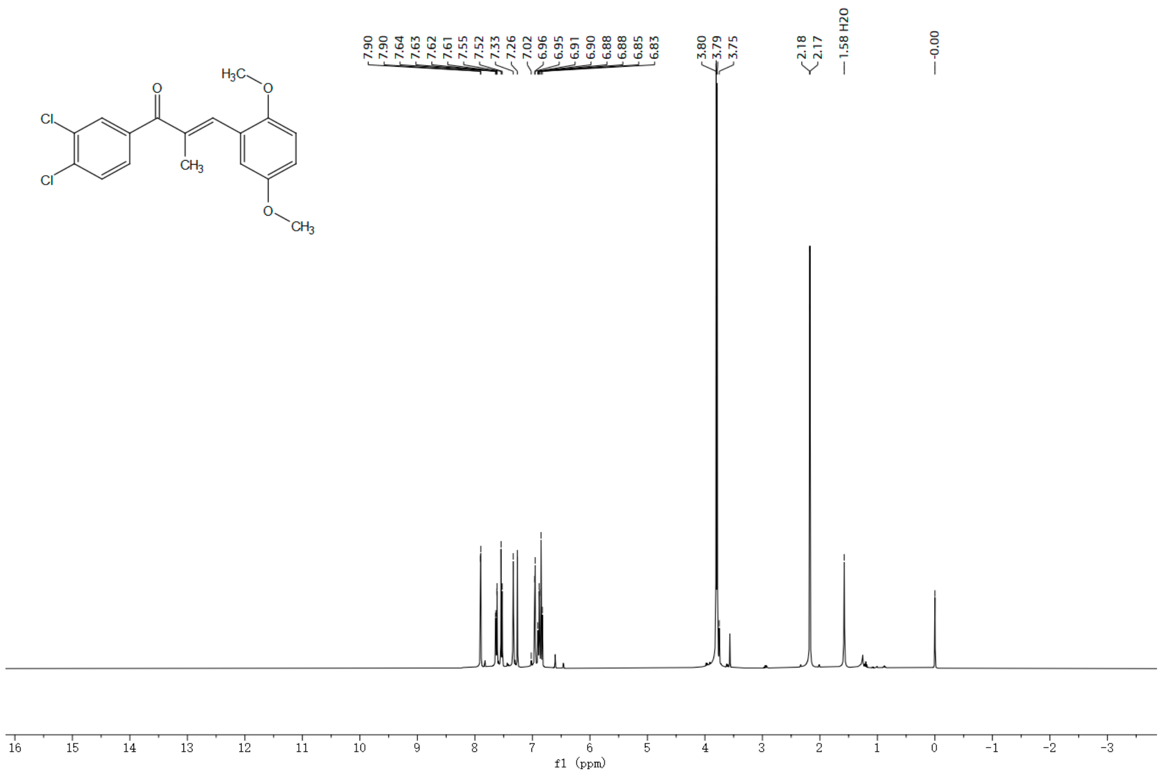

Figure. 10-2 ^{1}H NMR of compound 3j

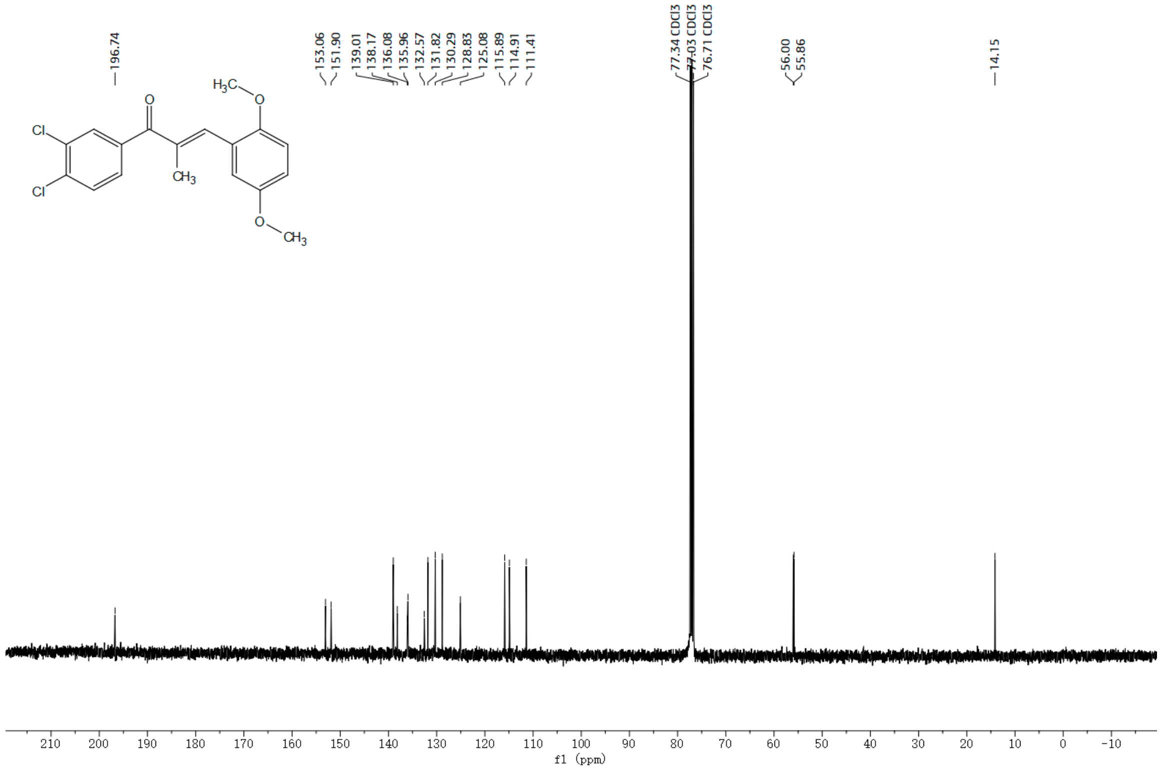

Figure. 10-3 ^{13}C NMR compound 3j

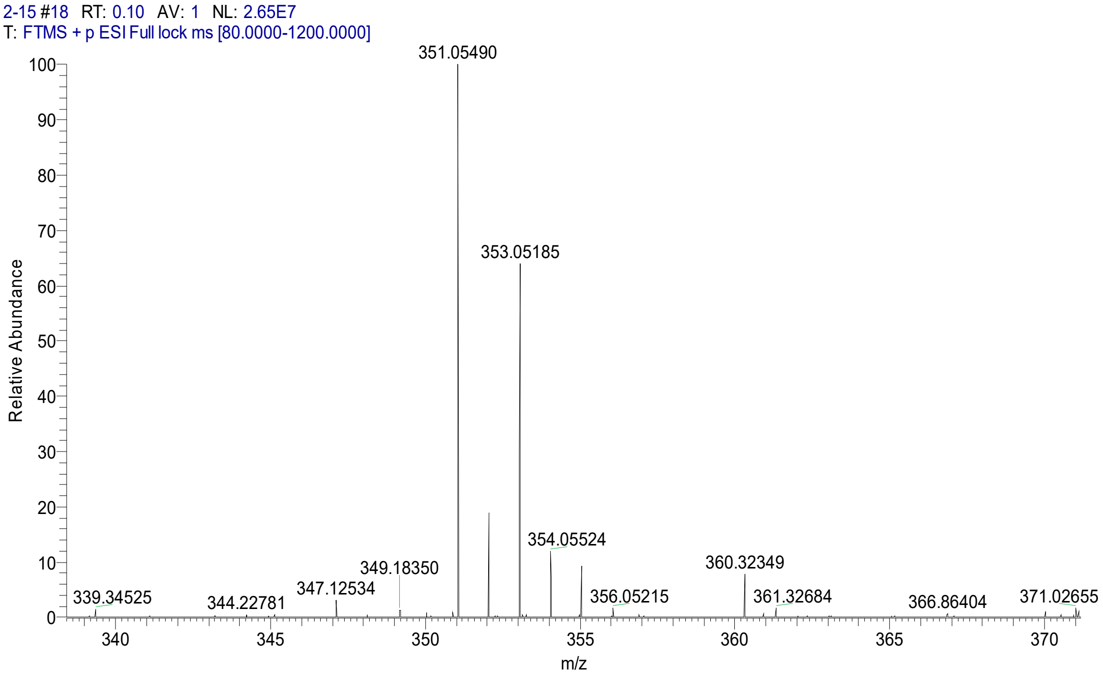

Figure. 11-1 Mass spectral data of compound 3k

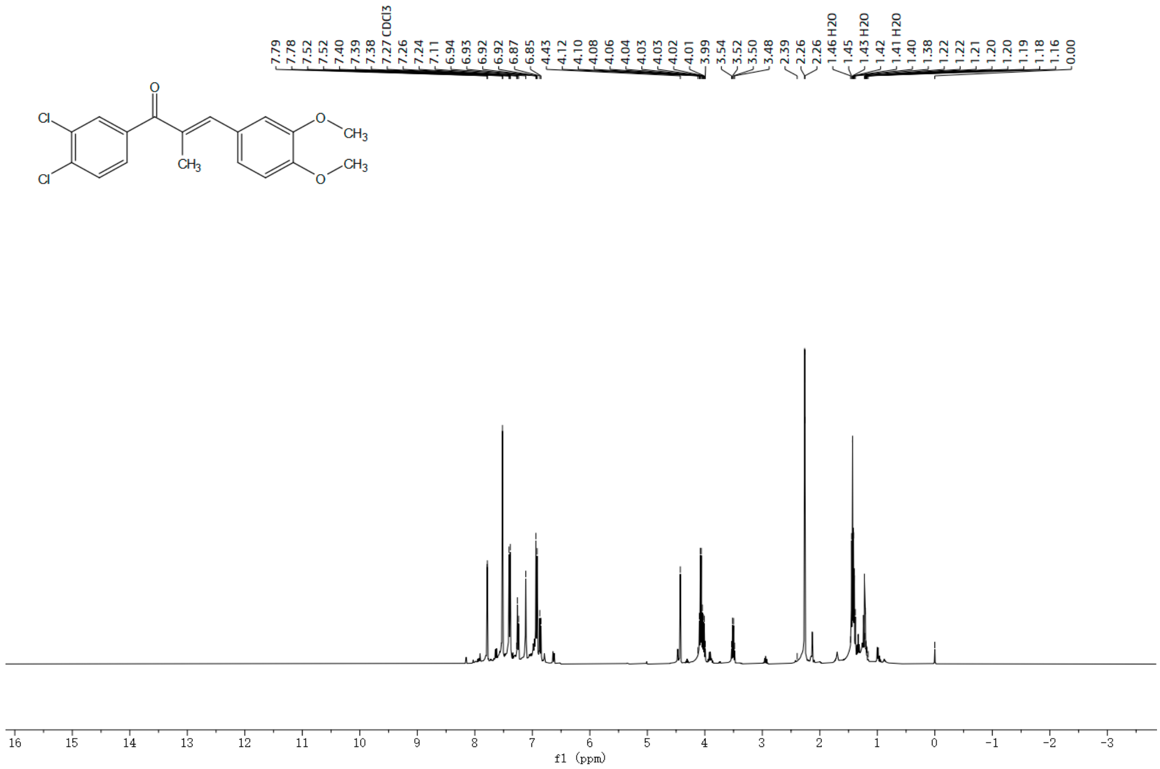

Figure. 11-2 ^{1}H NMR of compound 3k

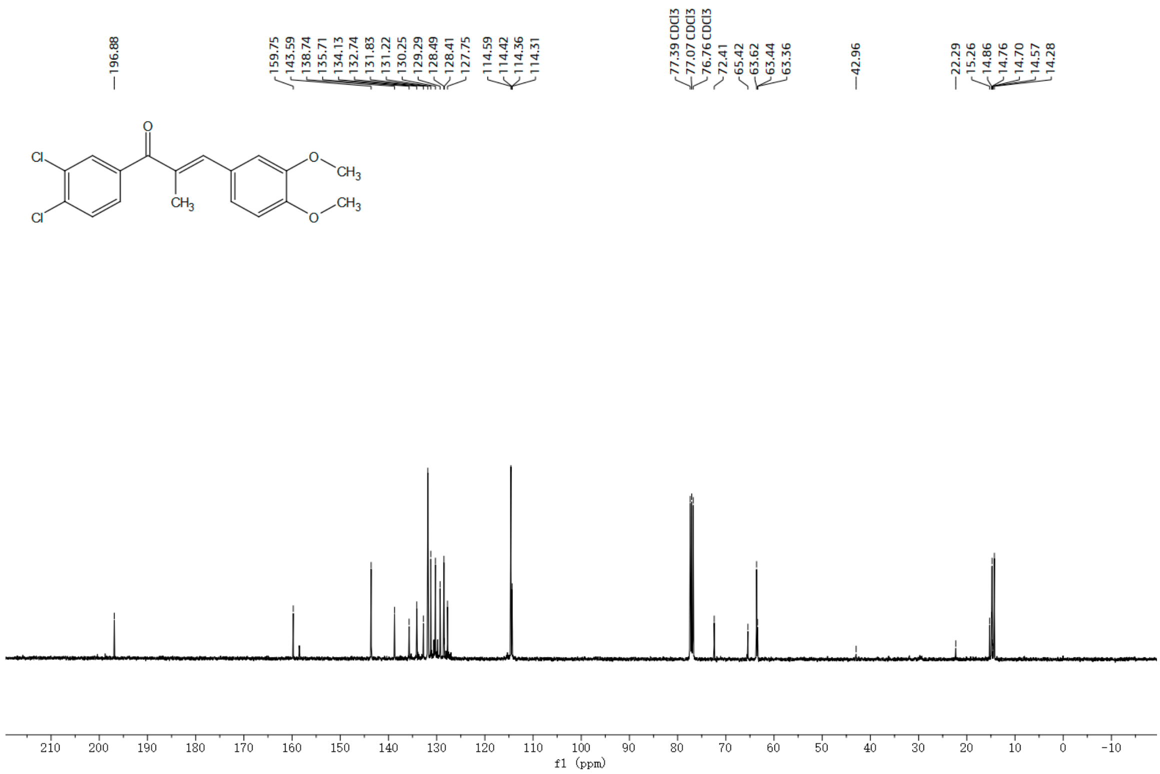

Figure. 11-3 ^{13}C NMR compound 3k

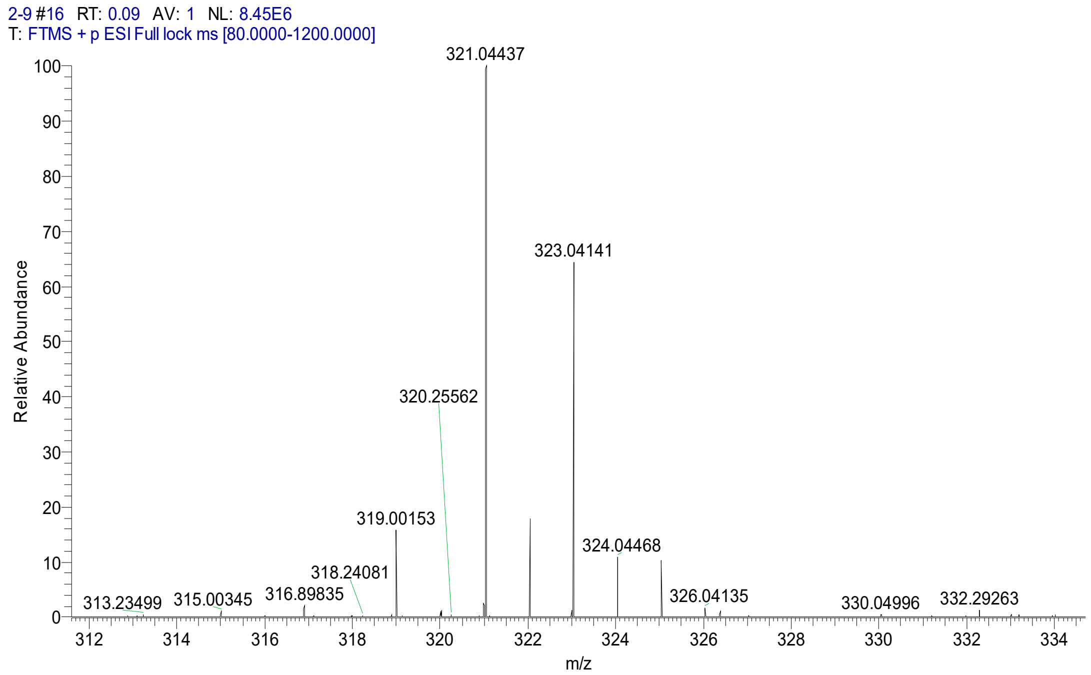

Figure. 12-1 Mass spectral data of compound 3l

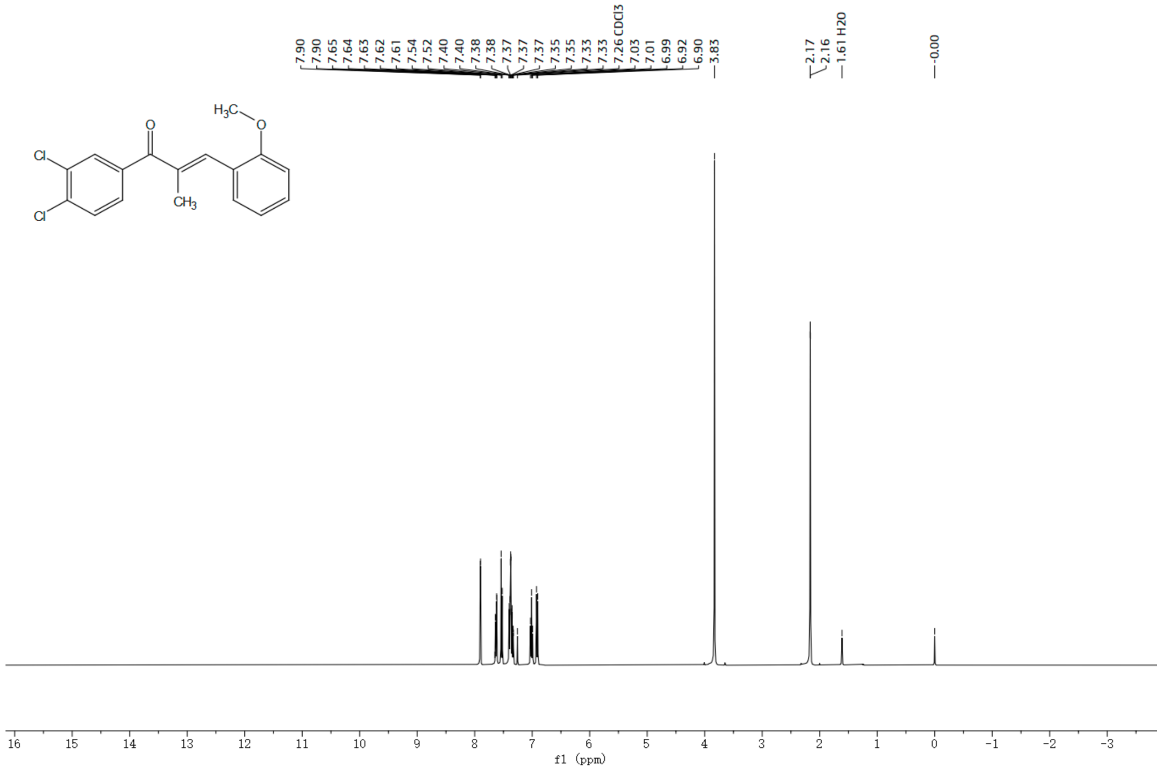

Figure. 12-2 ^{1}H NMR of compound 3l

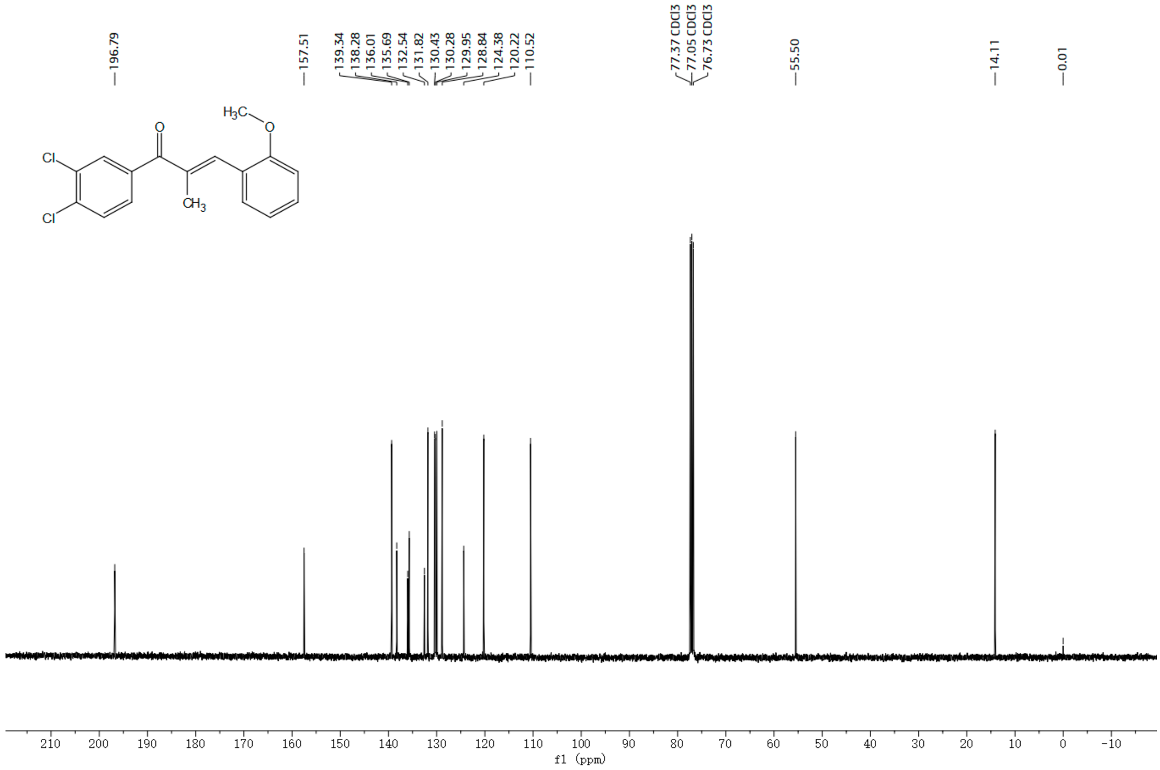

Figure. 12-3 ^{13}C NMR compound 3l

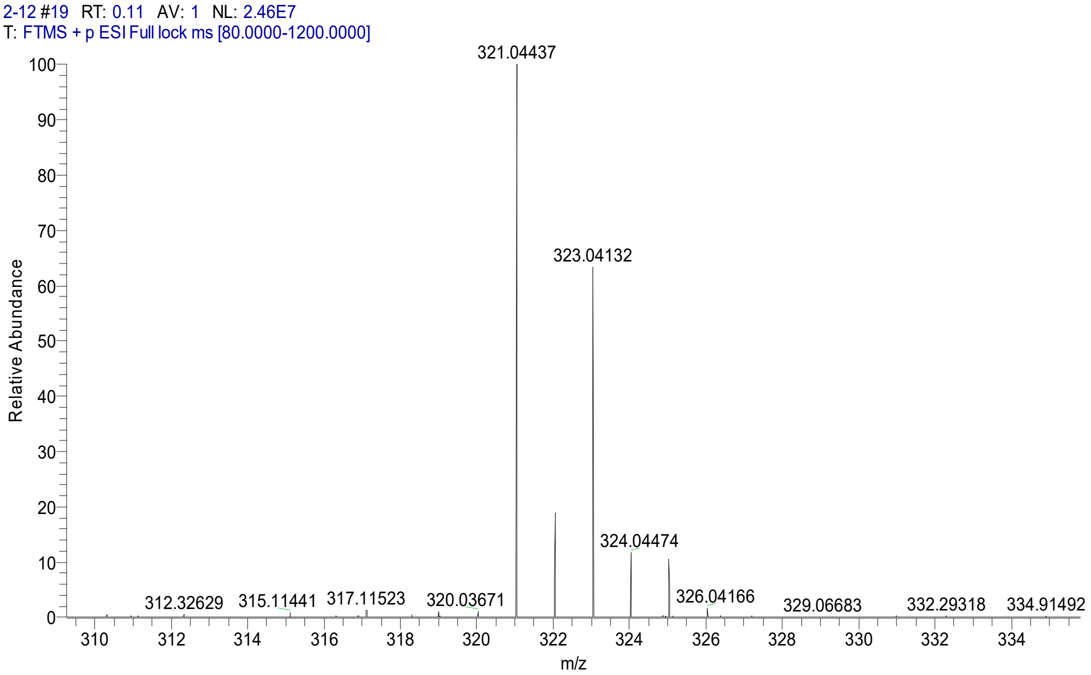

Figure. 13-1 Mass spectral data of compound 3m

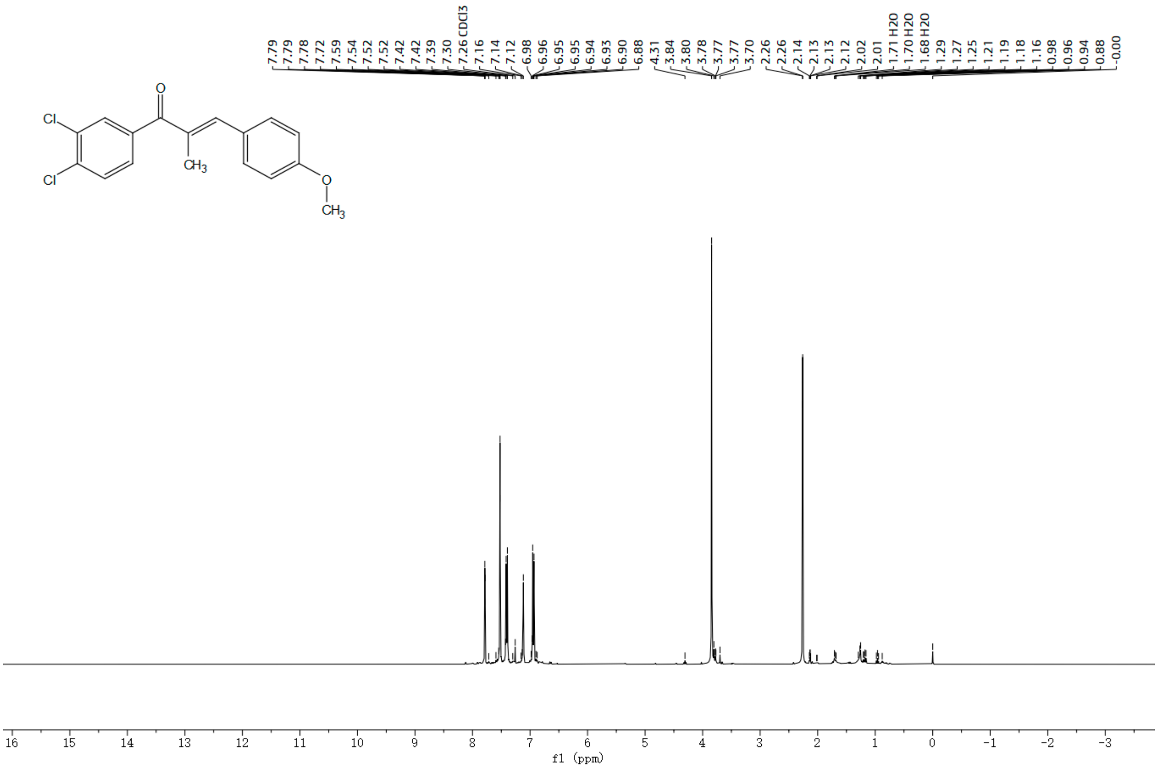

Figure. 13-2 ^{1}H NMR of compound 3m

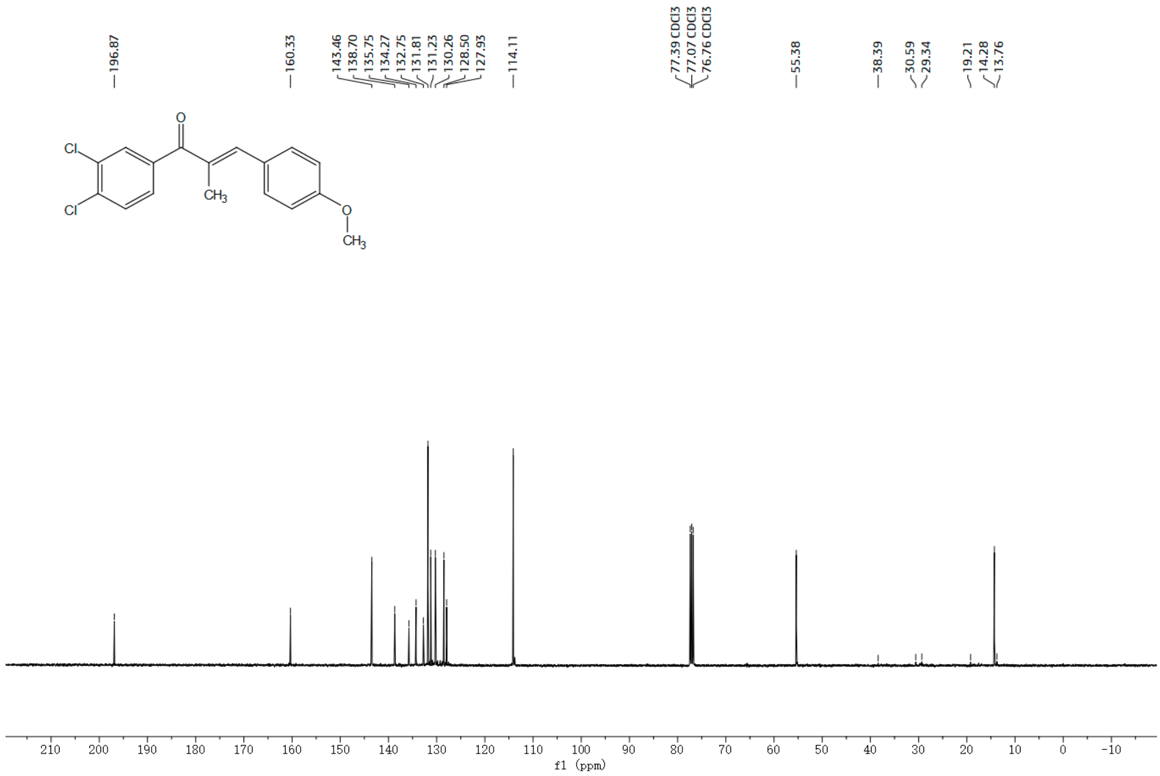

Figure. 13-3 ^{13}C NMR compound 3m

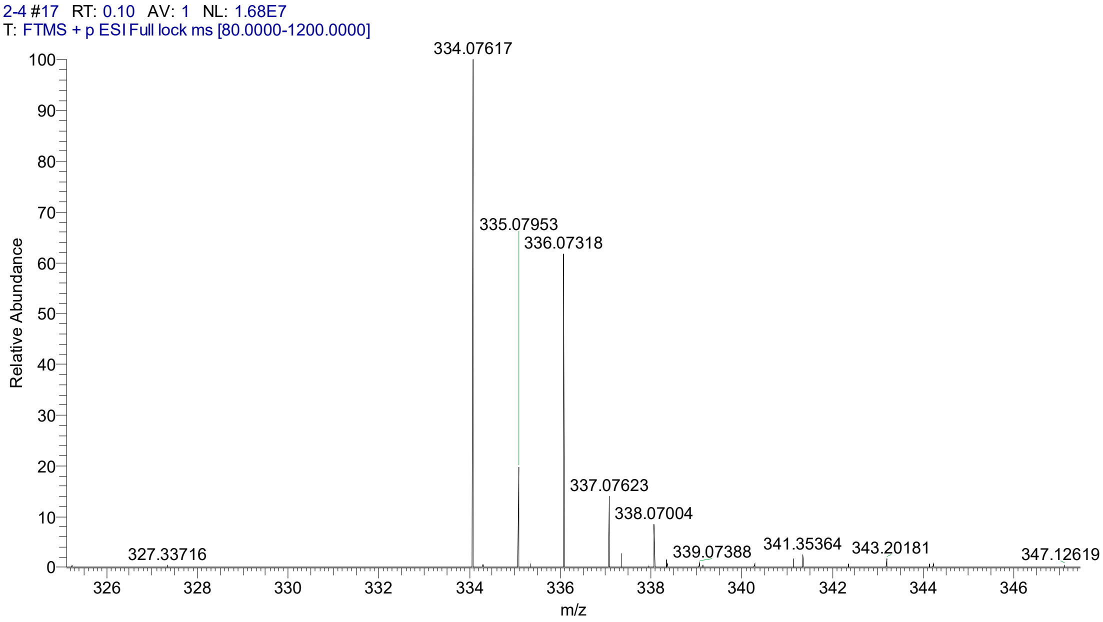

Figure. 14-1 Mass spectral data of compound 3n

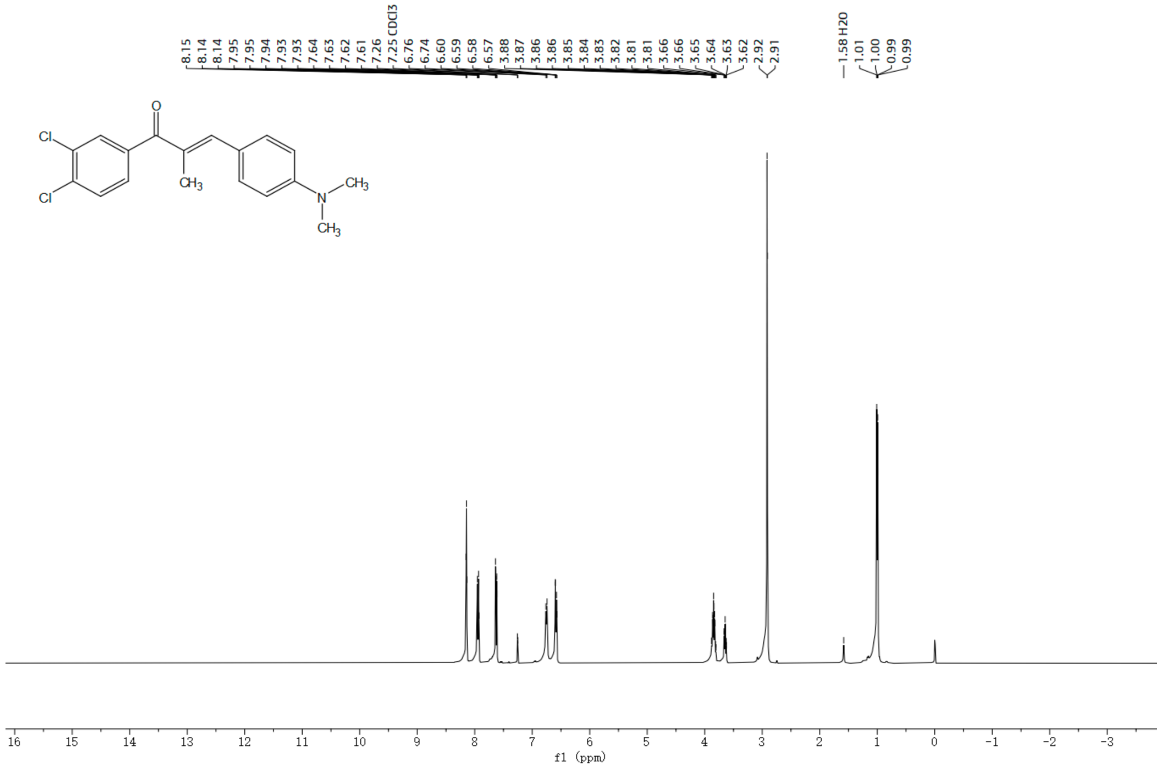

Figure. 14-2 ^{1}H NMR of compound 3n

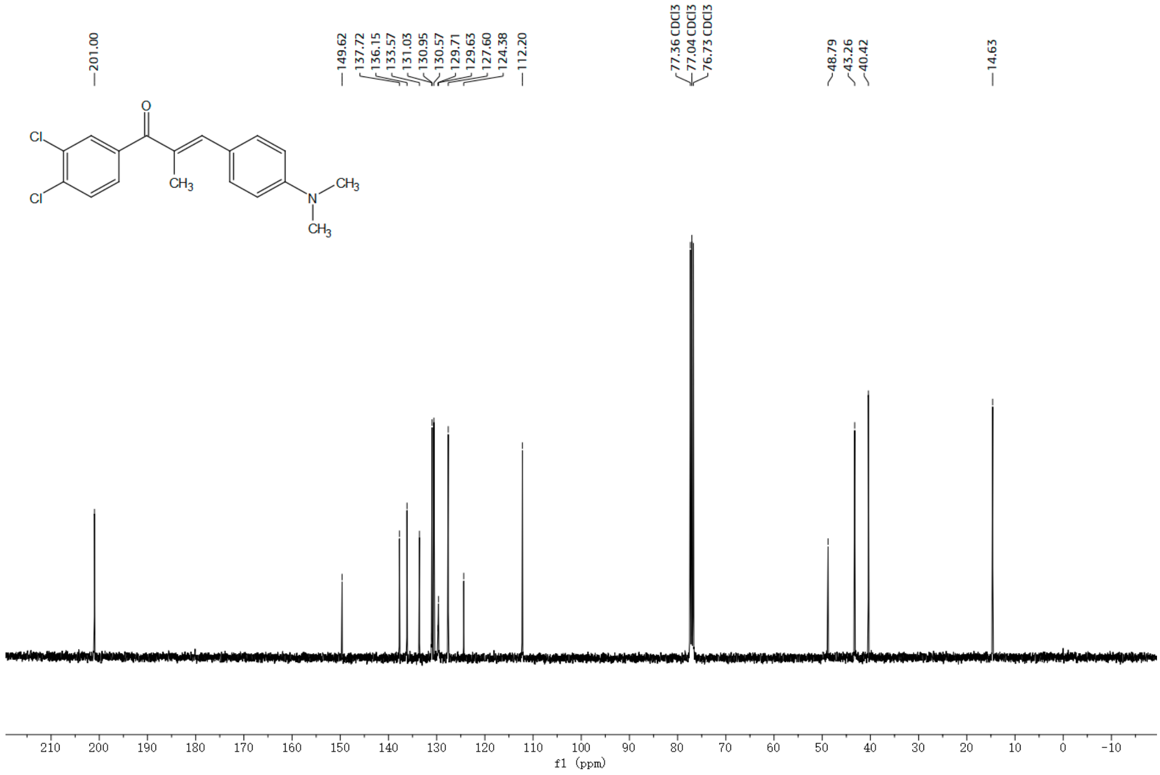

Figure. 14-3 ^{13}C NMR compound 3n

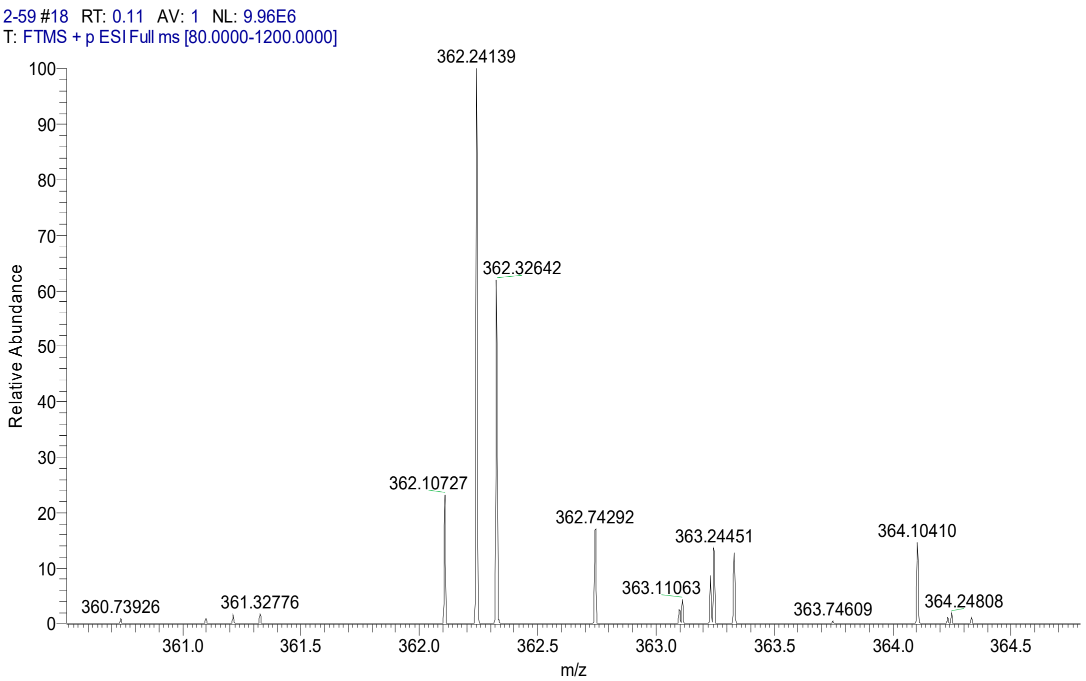

Figure. 15-1 Mass spectral data of compound 3o

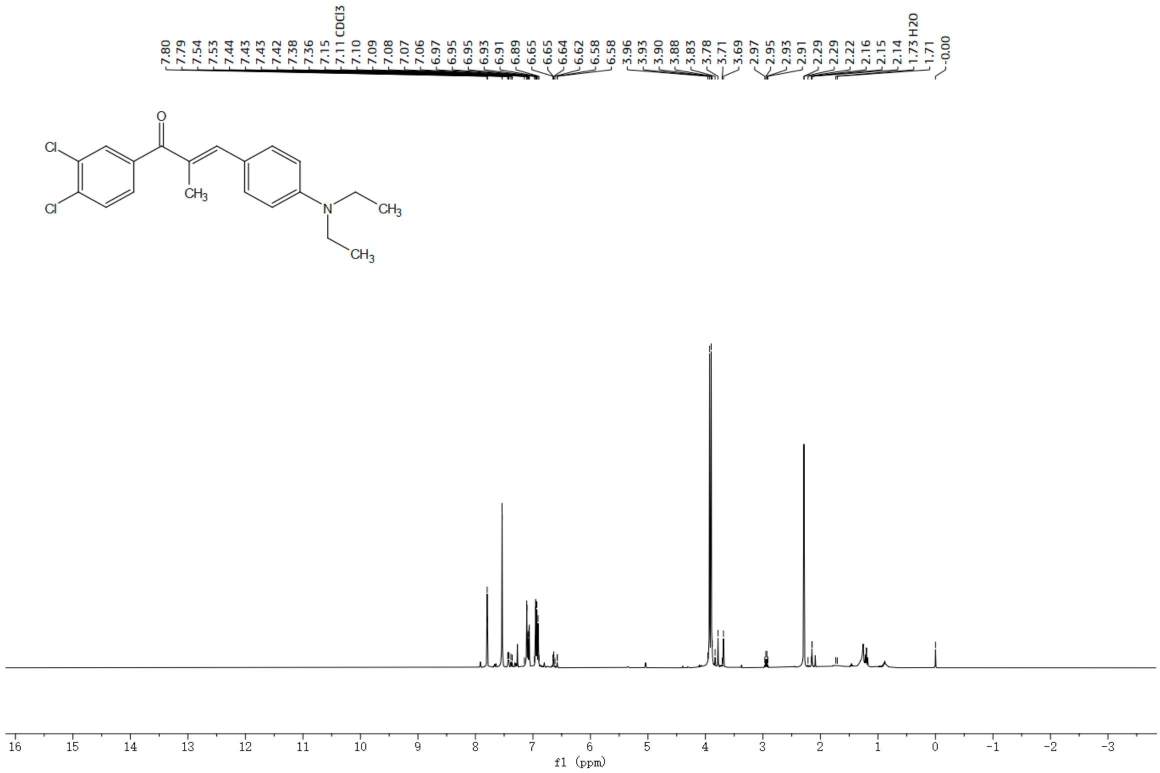

Figure. 15-2 ^{1}H NMR of compound 3o

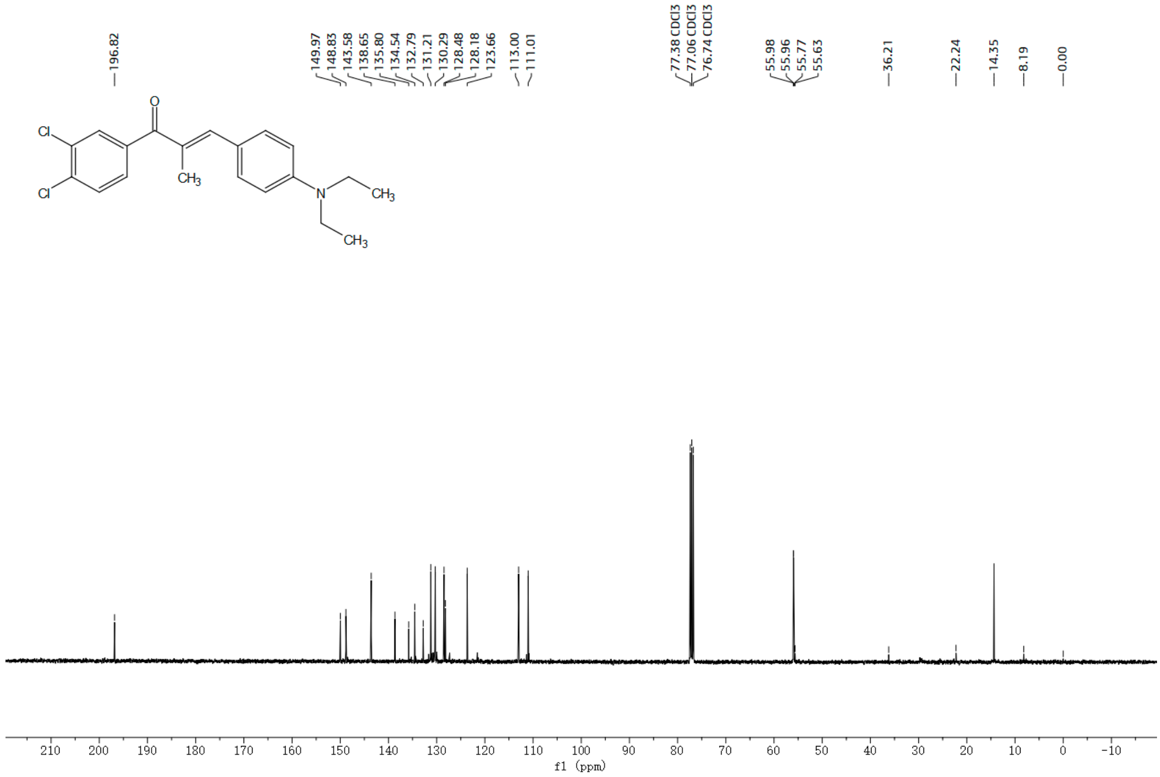

Figure. 15-3 ^{13}C NMR compound 3o

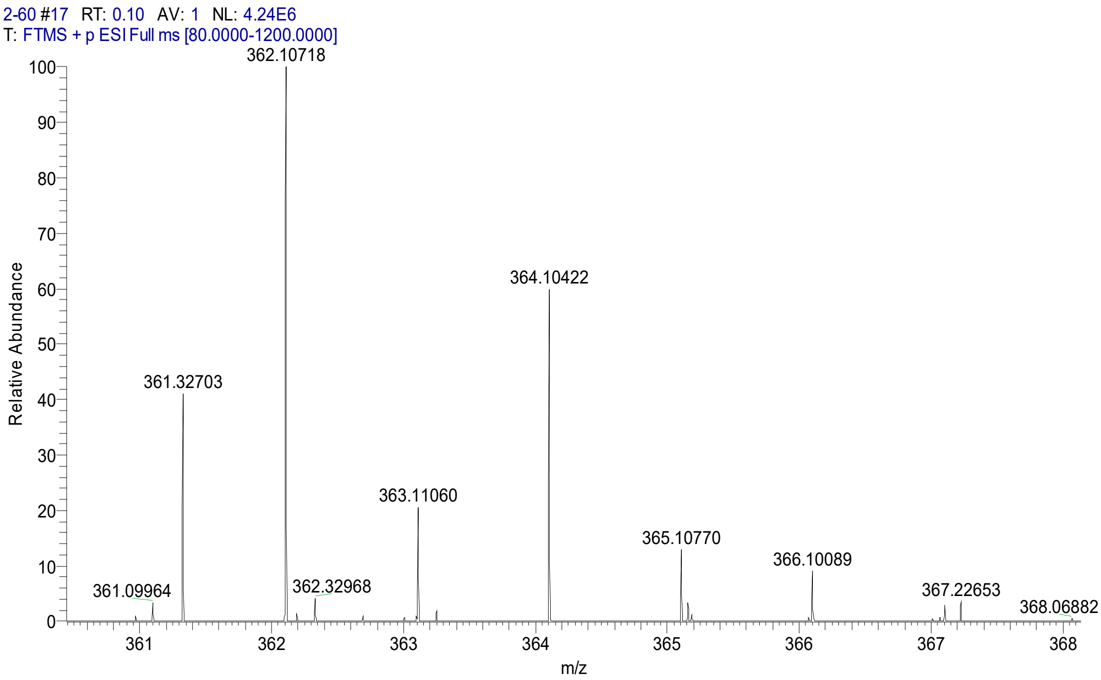

Figure. 16-1 Mass spectral data of compound 3p

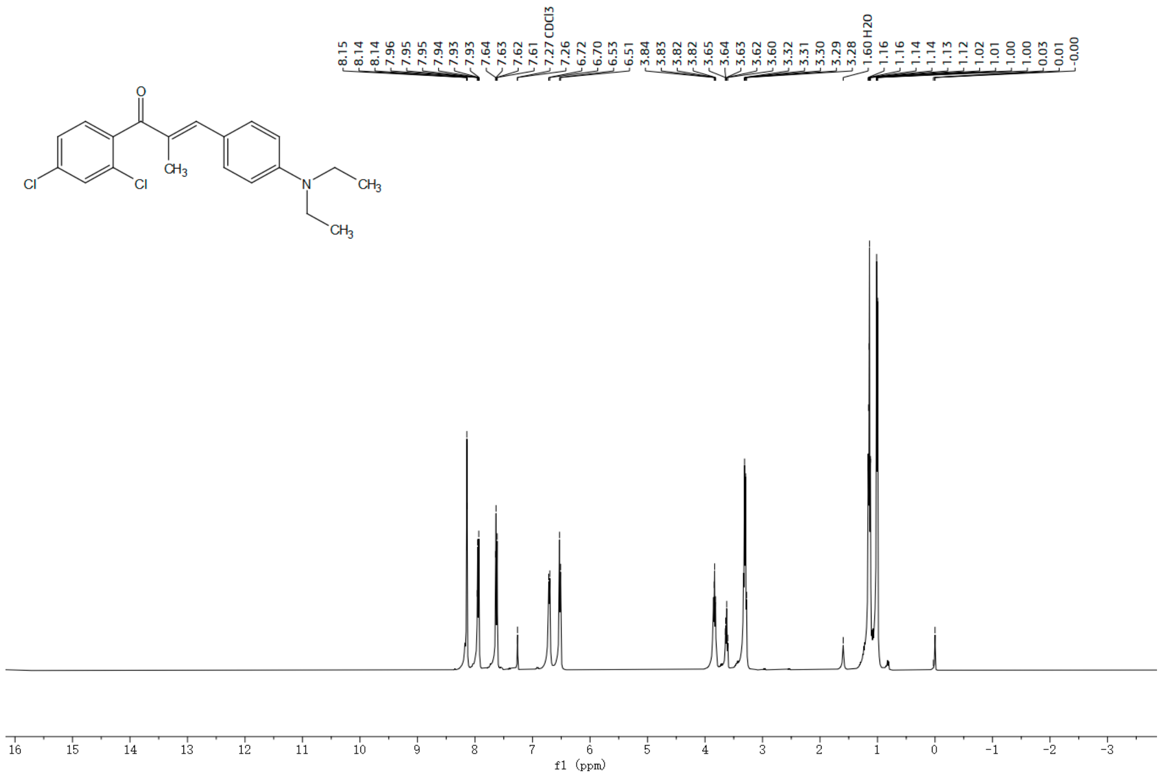

Figure. 16-2 ^{1}H NMR of compound 3p

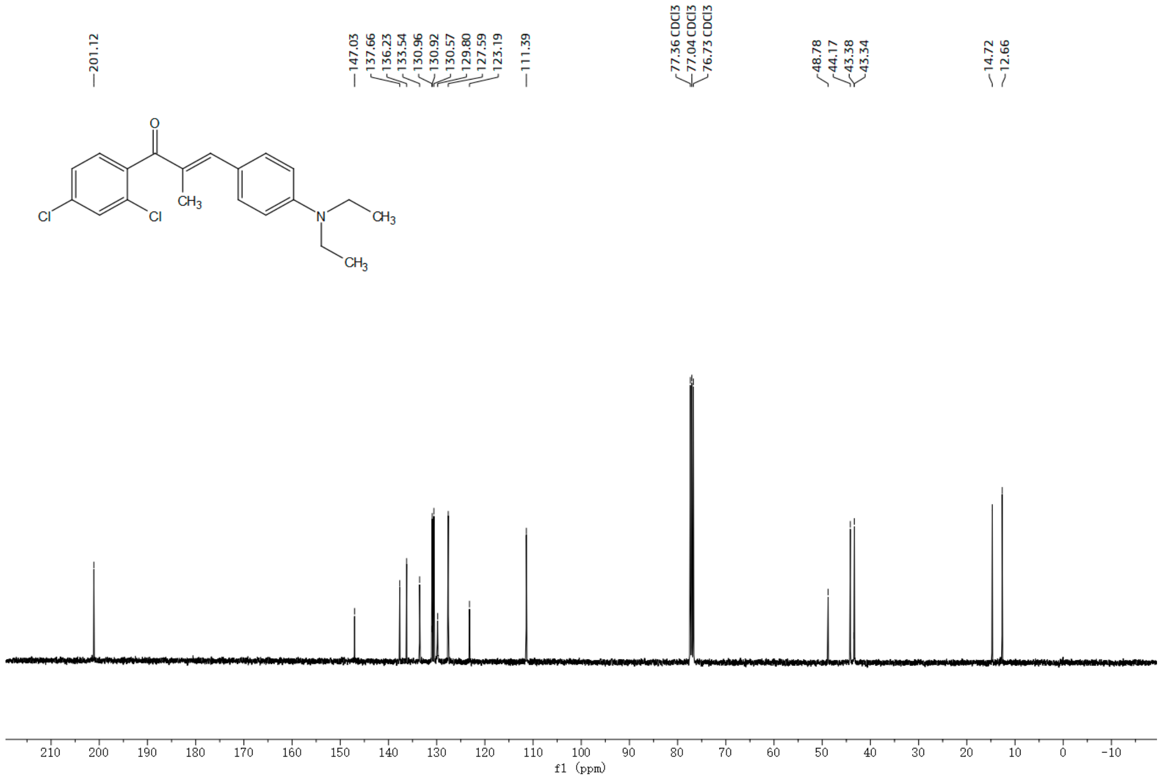

Figure. 16-3 ^{13}C NMR compound 3p
